# Identification of a type 1 diabetes–associated T cell receptor repertoire signature from the human peripheral blood

**DOI:** 10.1126/sciadv.adx7448

**Published:** 2026-02-13

**Authors:** Puneet Rawat, Melanie R. Shapiro, Leeana D. Peters, Michael Widrich, Koshlan Mayer-Blackwell, Keshav Motwani, Milena Pavlović, Ghadi al Hajj, Amanda L. Posgai, Chakravarthi Kanduri, Giulio Isacchini, Maria Chernigovskaya, Lonneke Scheffer, Kartik Motwani, Leandro Octavio Balzano-Nogueira, Camryn M. Pettenger-Willey, Sebastiaan Valkiers, Laura M. Jacobsen, Michael J. Haller, Desmond A. Schatz, Clive H. Wasserfall, Ryan O. Emerson, Andrew J. Fiore-Gartland, Mark A. Atkinson, Günter Klambauer, Geir Kjetil Sandve, Victor Greiff, Todd M. Brusko

**Affiliations:** ^1^ Department of Immunology, University of Oslo, Oslo, Norway.; ^2^ Department of Pathology, Immunology, and Laboratory Medicine, Diabetes Institute, College of Medicine, University of Florida, Gainesville, FL, USA.; ^3^ Institute for Machine Learning, Johannes Kepler University Linz, Austria.; ^4^ Fred Hutchinson Cancer Center, Seattle, WA, USA.; ^5^ Department of Biostatistics, University of Washington, Seattle, WA, USA.; ^6^ Department of Informatics, University of Oslo, Oslo, Norway.; ^7^ UiO:RealArt Convergence Environment, University of Oslo, Oslo, Norway.; ^8^ Imprint Labs LLC, New York, NY, USA.; ^9^ Center for Vaccine Innovation, La Jolla Institute for Immunology, La Jolla, USA.; ^10^ Whitman College, Walla Walla, WA, USA.; ^11^ University of Antwerp, Antwerp, Belgium.; ^12^ Department of Pediatrics, College of Medicine, University of Florida, Gainesville, FL, USA.; ^13^ Independent Researcher, Seattle, WA, USA.; ^14^ LIT AI Lab, Johannes Kepler University Linz, Linz, Austria.; ^15^ Department of Biochemistry and Molecular Biology, College of Medicine, University of Florida, Gainesville, FL, USA.

## Abstract

Type 1 diabetes (T1D) is a T cell–mediated disease with a strong immunogenetic human leukocyte antigen (HLA) dependence. HLA allelic influence on the T cell receptor (TCR) repertoire shapes thymic selection and controls activation of diabetogenic clones yet remains largely unresolved in T1D. We sequenced the circulating TCRβ chain repertoire from 2250 HLA-typed participants across three cross-sectional cohorts, including individuals with T1D and healthy related and unrelated controls. We found that HLA risk alleles show higher restriction of TCR repertoires in individuals with T1D. We leveraged deep learning to identify T1D-associated TCR subsequence motifs that were also observed in independent TCR cohorts residing in pancreas-draining lymph nodes of individuals with T1D. Collectively, our data demonstrate T1D-related TCR motif enrichment based on genetic risk, offering a potential metric for autoreactivity and groundwork for TCR-based diagnostics and therapeutics.

## Teaser

Large-scale TCR repertoire profiling from peripheral blood reveals type 1 diabetes signatures linked to high-risk HLA alleles.

## INTRODUCTION

Antigen-specific recognition of both foreign and autoantigens is enabled by T cell receptors (TCRs). Elucidating the TCR sequences implicated in type 1 diabetes (T1D) is crucial for advancing our understanding of disease pathogenesis and facilitating the development of reliable biomarkers (*1*, *2*). Human leukocyte antigen (HLA) loci shape the TCR repertoire (*3*–*5*), and HLA risk alleles have been shown to influence TCR clonal sequences in various autoimmune diseases, including rheumatoid arthritis (RA), primary sclerosing cholangitis (PSC), T1D, and celiac disease (CD) (*6*–*8*). In T1D, HLA class II loci account for most of genetic risk (*9*, *10*), with *DR3* and *DR4* alleles conferring the highest risk (*11*). Seropositivity for islet autoantibodies in combination with genetic risk can provide population-level estimates of the rate of T1D progression, with longitudinal studies illustrating the influence of HLA on type of initial seroconversion [e.g., insulin autoantibody (IAA) versus GAD autoantibody (GADA) first] and progression to multiple autoantibodies (*12*). The development of T cell biomarkers could enhance the monitoring of disease progression and improve current predictive methods for presymptomatic disease detection (*13*). However, efforts to develop T1D biomarkers have been constrained by a reliance on preexisting knowledge of autoimmune targets and cellular mechanisms in T1D (*14*). There remains a critical need for immunogenetic studies that explore the link between TCRs and HLA in the context of T1D and for the identification of TCR-based biomarkers (*15*).

Analyses of the adaptive immune receptor repertoire (AIRR) in infectious and autoimmune disease contexts have demonstrated that the TCR repertoire may be used to develop novel diagnostics (*16*–*23*). Longitudinal studies of healthy adult cohorts showed repertoire stability, indicating the robustness of TCR-based signatures for identifying sustained disease repertoire perturbations (*24*). However, autoimmunity-associated immune receptor signals, in contrast to those associated with infection and cancer, were initially found to be small or nearly undetectable when considering global repertoire metrics (*16*, *25*–*28*). Therefore, analytical techniques, such as machine learning (ML) platforms, have been used to detect even the smallest shifts in signals within adaptive immune repertoires. For example, repertoire alterations have been documented in response to cytomegalovirus (CMV) (*20*, *29*), cancer (e.g., lymphoma and tumor-infiltrating lymphocytes) (*30*, *31*), therapeutic responses to checkpoint inhibitors in melanoma (*32*), as well as systemic autoimmune diseases including multiple sclerosis (*33*), systemic lupus erythematosus (SLE) (*34*), and RA (*19*, *35*) using statistical analyses and ML approaches on AIRR sequencing data with reasonable accuracy [area under the receiver operating curve (AUROC) > 0.75]. ML methods have demonstrated the capability to classify immune repertoires for a given clinical status and to recover immune signals associated with specific clinical conditions (*36*–*38*).

While previous reports had indicated that islet autoantigen-reactive cells are present at similar frequencies in the peripheral blood of control and T1D study participants (*39*–*43*), others have observed increased expansion of antigen-specific clonotypes in T1D, indicating a disease signature in the periphery (*44*). Most of islet-antigen–specific complementarity-determining region 3 beta chain (CDR3β) sequences were found to be private (*2*) or observed at the individual level, demonstrating a need for subsequence (motif)–based biomarkers.

We sought to identify a T1D-associated T cell repertoire signature from bulk peripheral blood mononuclear cells (PBMCs) to provide a translationally relevant biomarker. To this end, we sequenced 2250 TCRβ repertoires across the natural history of T1D to (i) investigate the existence of HLA-restricted, T1D-associated CDR3β sequences and motifs; (ii) assess the feasibility to classify individuals as having T1D based on the TCR repertoire; and (iii) identify T1D-associated signatures within TCR repertoires. Our study reveals that the TCR repertoire lacks shared public clones across clinical groups. However, a substantial enrichment of TCR repertoires at the subsequence level was observed, both with and without accounting for genetic risk factors. These findings suggest that TCR repertoire analysis may offer valuable insights into autoreactivity, potentially serving as a basis for developing TCR-based diagnostic tools and therapeutic strategies.

## RESULTS

### Overview of the dataset and reproducibility assessment

**Fig. 1.**
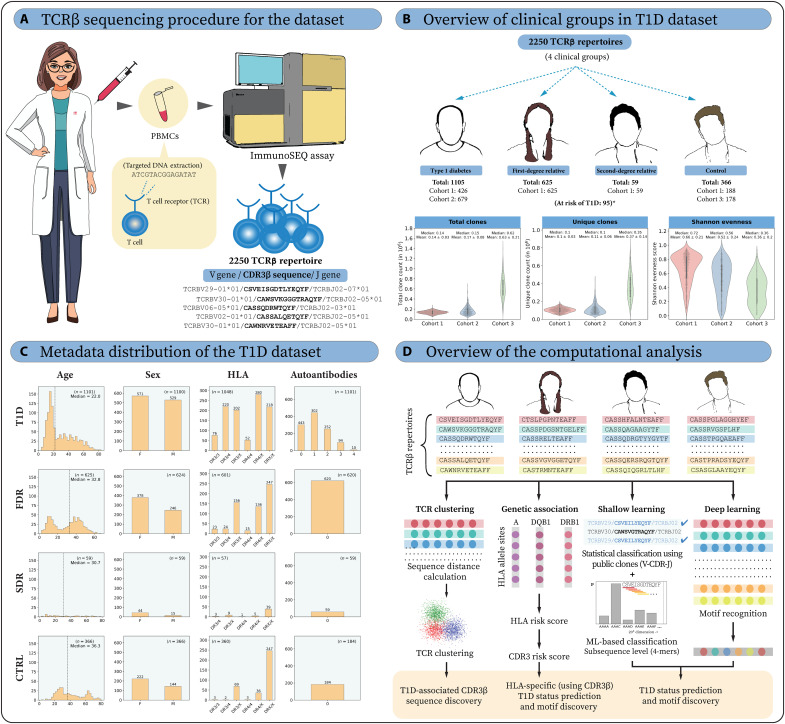
Overview of T1D TCRβ cohorts and the study. (**A**) DNA was isolated from PBMCs to conduct TCRβ chain sequencing. (**B**) The dataset of 2250 TCRβ repertoires contains four clinical groups: T1D (T1D), first-degree relatives (FDR), second-degree relatives (SDR), and non-related controls (CTRL) (*excluding 95 individuals without diabetes who have an increased risk of developing T1D, i.e., AAb^+^), which were sequenced in three different cohorts (see Methods for cohort-specific sequencing details). The unique number of clones, total number of clones (unique clones and their clonal frequency), and Shannon evenness are shown for each cohort. Mean and median values are presented above each violin plot. (**C**) The distribution of age (dotted line shows median value), sex ratio, high-risk human leukocyte antigen (HLA) and number of autoantibodies (IAA, GADA, ZnT8A, and IA-2A) present (for cohorts 1 and 2) are shown for the four clinical groups across all three cohorts. (**D**) In this study, we leveraged available HLA information to conduct HLA and V-gene–restricted TCR clustering and to elucidate the extent to which high-risk HLAs restrict CDR3β sequences. The influence of HLA-mediated T1D risk was translated into TCR repertoire–based T1D risk and identification of CDR3β motifs. Furthermore, we applied ML/DL techniques to classify T1D and healthy related and unrelated controls. These computational approaches were also used to identify T1D-associated TCRβ sequence motifs.

To study T1D-associated TCR repertoire alterations, we immunosequenced the rearranged CDR3 TCRβ region in bulk PBMCs of three different cross-sectional cohorts (Fig. 1). Cohort 1 contains 1393 repertoires (103,176 ± 26,850 unique CDR3β sequences) distributed across the natural history of T1D from the University of Florida Diabetes Institute (UFDI) biobank. These include individuals diagnosed with T1D (*n* = 426; 30.5%), first-degree relatives of individuals with T1D (FDR; *n* = 625; 45%), second-degree relatives of individuals with T1D (SDR; *n* = 59; 4%), unrelated healthy control individuals (CTRL; *n* = 188; 13.5%), and islet autoantibody–positive individuals without diabetes who have increased risk of developing T1D (AAb^+^; *n* = 95; 7%). Although statistically significant differences in unique CDR3β sequences were observed between clinical groups (<0.05), the difference in mean, median, and SD was minimal (fig. S1). Cohorts 2 and 3, respectively, contain 679 T1D repertoires (112,637 ± 55,091 unique CDR3β sequences) and 178 deep sequenced CTRL repertoires (366,271 ± 143,197 unique CDR3β sequences), the latter sequenced with a different protocol than cohort 1 (see Methods). We obtained high reproducibility and adequate sequencing depth to capture the clonal diversity of TCRs using technical replicates (fig. S2).

In addition to TCRβ sequencing, the dataset has extensive metadata in the form of whole-genome [>978,000 single-nucleotide polymorphism (SNP)] data derived from a custom Affymetrix array known as the UFDIchip (*45*), clinical data (HbA1c, C-peptide, gender, age, and T1D duration), and serological data {islet AAb number and specificity [IAA, GADA, zinc transporter 8 (ZnT8A), and insulinoma-associated protein 2 (IA-2A)]; (*46*)} (fig. S1). We used precision genotyping (*45*) and HLA imputation (*47*) techniques to obtain four-digit classical alleles for the major histocompatibility complex (MHC) class I and II genes. Specifically, we obtained four-digit HLA for 1332 (95.6%) individuals, including all AAb^+^ repertoires in cohort 1, 645 (95%) individuals in cohort 2, and 178 (100%) individuals in cohort 3. Most of the primary analyses in this work were based on cohort 1 as it is the largest cohort among the three and also contains all clinical statuses. Cohorts 2 and 3 served as test cohorts for ML analysis unless specified otherwise.

### Repertoire-level similarity and diversity do not differ between T1D clinical groups

**Fig. 2.**
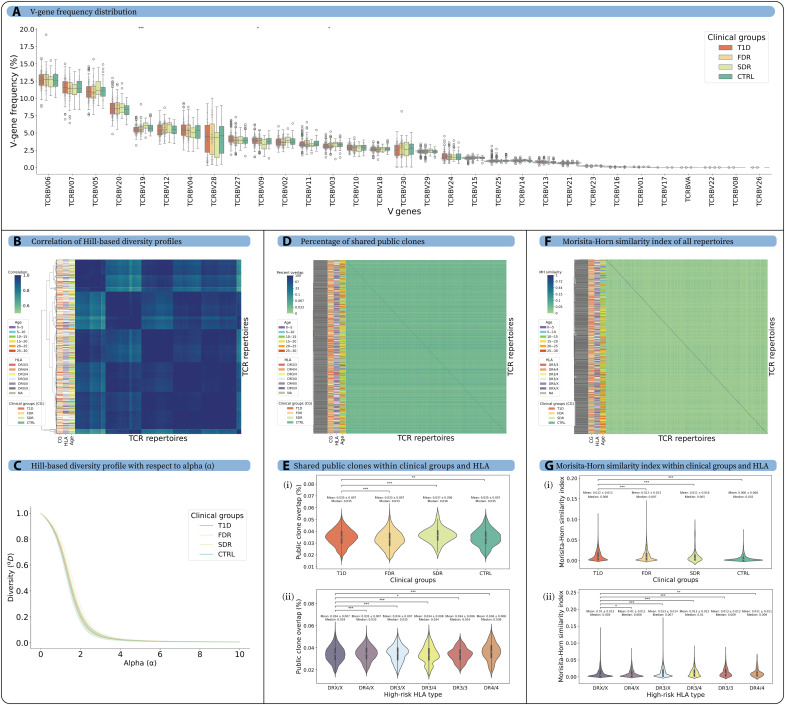
The V-gene distribution, diversity, and clonal overlap of the TCRβ repertoires were similar across the T1D, FDR, SDR, and CTRL groups. The analysis was performed on individuals in cohort 1, younger than 30 years of age, to minimize age-based confounding factors. (**A**) The V-gene distribution was similar across TCRβ repertoires of different clinical groups. The frequency of each V gene was averaged for all repertoires in each clinical group, and difference in V-gene distribution was tested for all clinical groups. (**B**) Hill-based diversity profiles were calculated for alpha (α) value range [0, 10] and step size of 0.2, and Shannon evenness was used for the undefined value of α = 1 for each repertoire. The heatmap shows the Pearson correlation of Hill-based diversity profiles for each pair of repertoires (see Methods). (**C**) The average Hill-based diversity profile for each α value of TCRβ repertoires calculated for different clinical groups. (**D**) The heatmap represents the percentage of public clones shared between each pair of repertoires. There was very low overlap among pairs of repertoires (maximum public clone overlap of 0.08%). (**E**) The percentage of public clone overlap (i) among the clinical groups and (ii) among the high-risk HLA types (*DR3/DR4*). (**F**) The Morisita-Horn (MH) similarity index calculated for each pair of repertoire shows low overlap among TCRβ repertoires (maximum MH similarity index of 0.34), and no clustering was observed on the basis of age, clinical group, or high-risk HLA. (**G**) The MH similarity index (i) within the clinical groups and (ii) within the high-risk HLA types. A universal color scheme was used to show T1D, FDR, SDR, and CTRL in red, yellow, light green, and dark green, respectively. *P* values were described as * for [0.01, 0.05], ** for [0.001, 0.01], and *** for <0.001. Only significant *P* values were displayed.

Repertoire-level similarity and diversity analyses are widely used for cross-individual comparisons of AIRRs (*18*, *25*, *48*–*51*). We assessed immune repertoire diversity and similarity across clinical groups within cohort 1 and restricted our analysis to the 779 individuals under age 30 to mitigate potential age-related confounding factors (*52*). We assessed *TRBV*-gene usage (Fig. 2A) as previous studies in other autoimmune diseases (e.g., SLE and RA) revealed distinct V-gene usage patterns that may be associated with different disease states (*19*, *53*). We observed a small number of statistically significant differences in V-gene usage; however, the absolute differences were biologically insubstantial. For example, we observed a significant difference in *TRBV19* (*P* = 1.14 × 10^−4^) gene usage across clinical groups. Yet, the difference in average frequency of V genes in T1D (5.49 ± 0.58) and CTRL (5.75 ± 0.66) repertoires was minimal, in line with previous reports on other T1D cohorts (*41*).

To compare repertoires on the sequence level, we defined public clones as identical CDR3β sequences shared among two repertoires, ignoring the V- and J-gene information. To compare clonal expansion, we calculated the diversity of the immune repertoires using Hill-based diversity profiles (*48*). The α-parameterized diversity profile consolidates many previously established diversity indices [SR, ^α=0^*D* (*54*, *55*); Shannon, ^α=1^*D* (*56*, *57*); Simpson’s, ^α=2^*D* (*58*, *59*); and Berger-Parker, ^α=∞^*D* (*54*, *60*)]. We calculated the correlation between the diversity profile of each repertoire and clustered them according to clinical groups, conventional high-risk HLA types (*DR3* and *DR4*), and age (Fig. 2, B, C). However, the diversity profile did not cluster on the basis of these parameters.

Furthermore, we calculated the similarity between repertories on the basis of two metrics: (i) percentage of shared public clones for all repertoire pairs with respect to the total number of clones present in the smaller repertoire (based on clonal overlap) (*61*) and (ii) Morisita-Horn (MH) index (based on clonal overlap and clonal frequency). Neither the public clone percentage nor the MH index clustered on the basis of clinical status, age, or high-risk HLA types (*DR3* and *DR4*) (Fig. 2, D and F). Although the observations were statistically significant across clinical groups, the differences in median values were minor and lacked biological relevance for percentage of public clones (median values range from 0.033 to 0.036%) and MH index (median values range from 0.002 to 0.008). Similarly, conventional high-risk HLA alleles were not associated with biologically relevant differences in percentages of public clones (median values range from 0.034 to 0.036) and MH index (median values range from 0.005 to 0.01) (Fig. 2, E and G). In summary, repertoire diversity and similarity analyses did not reveal biologically meaningful differences between clinical groups or individuals with different HLA genetic risk factors.

### Inflammatory disease–associated CDR3β sequences are overrepresented in T1D repertoires

Because no discernible repertoire-level similarity or diversity differences were observed across clinical groups without TCR-level antigen information, we next investigated whether incorporating publicly available information on T1D and other disease-associated CDR3β sequences from the McPAS and VDJdb databases (table S1) would show differences between clinical groups (*62*–*64*). Using McPAS data, we found that T1D repertoires had a slightly higher percentage of T1D-associated CDR3β sequences (0.096 ± 0.047) compared to other clinical groups (FDR, 0.093 ± 0.073; SDR, 0.09 ± 0.061; and CTRL, 0.094 ± 0.043, with the Kruskal-Wallis test *P* value of 0.0036; fig. S3A and table S2). However, these T1D-associated CDR3β sequences were not sufficient to cluster the repertoires by clinical group, high-risk HLA, or age (fig. S3B). Similarly, we observed a significant overrepresentation of CD and influenza-associated CDR3β sequences in T1D repertoires (fig. S3A and table S2) (*65*–*67*). T1D and CD share genetic drivers, the most notable being shared high-risk HLA (*DQ2.5*/*DQ8*) (*68*). Clinical guidelines emphasize vaccination for individuals with T1D, and the overrepresentation of influenza-associated CDR3β sequences might reflect increased vaccination compliance at the population level. Using VDJdb data, we observed a significant enrichment in hepatitis C virus (HCV)–, HIV-, and severe acute respiratory syndrome coronavirus 2 (SARS-CoV-2)–associated sequences in T1D repertoires compared to those in other clinical groups (fig. S3C and table S2). Despite the absence of a direct genetic link, several case reports have shown potential associations of T1D with HCV (*69*, *70*), HIV (*71*, *72*), and SARS-CoV-2 (*73*). The SARS-CoV-2–specific CDR3β sequences observed in our cohort likely represent potential cross-reactive CDR3β sequences with other human coronaviruses as all participant samples were collected before the COVID-19 pandemic.

The association of CMV with T1D remains unclear, with prior reports suggesting both a lower (*74*) and higher prevalence (*75*) of T1D in individuals with CMV. A total of 2118 CMV-associated CDR3β sequences in McPAS observed no significant association with T1D status in our study. We further used a collection of 25,508 unique CDR3β sequences associated with CMV exposure curated from the literature (*76*) and observed a significant reduction in the presence of CMV-associated CDR3β sequences in the T1D repertories compared to those in other groups in cohort 1 (fig. S3D). Our observation supports findings of a recent study performed on a larger cohort (*74*), suggesting that early childhood CMV infection may decelerate the progression to clinical T1D. However, the limited availability and poor validation of disease-specific sequences and the minimal strength of observed associations highlight the need for a more comprehensive analysis using advanced methodologies (*77*). Furthermore, beyond CMV, we identified potential links between T1D progression and several other diseases, including CD, influenza, HCV, HIV, and SARS-CoV-2.

### HLA risk alleles demonstrate higher restriction of TCR repertoire diversity in individuals with T1D

#### Limited T1D association of HLA-associated public TCRs

**Fig. 3.**
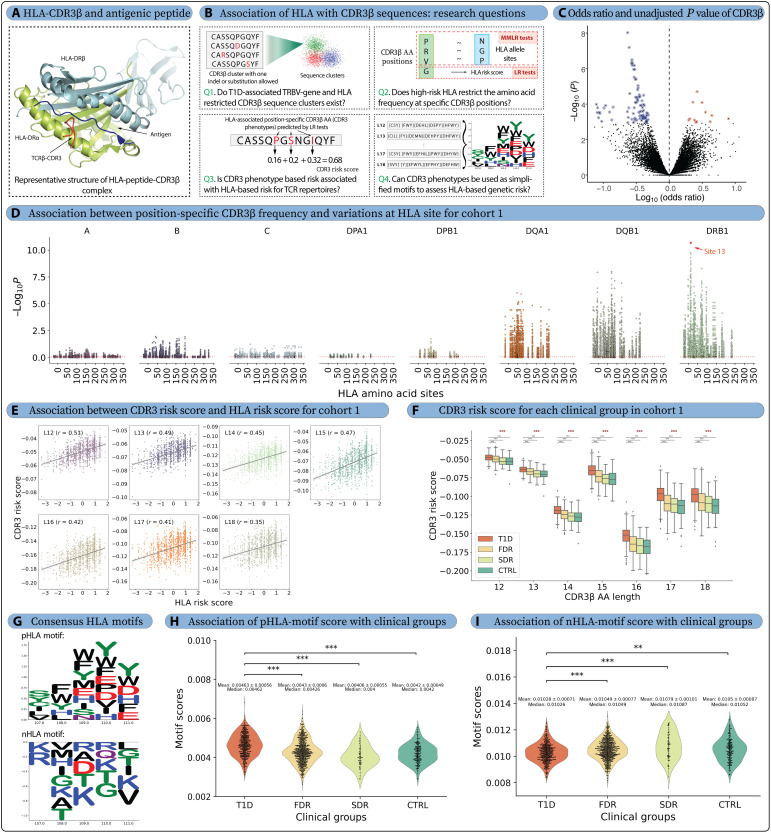
High-risk HLA alleles restrict the amino acid frequency of CDR3β sequences. (**A**) A representative complex structure of the human CDR3β region, influenza HA antigen peptide, and MHC class II molecule, *HLA-DR4* (Protein Data Bank ID: 1J8H), showcasing the potential impact of HLA on CDR3β sequences. (**B**) This study addressed four key questions related to HLA restriction: Q1 finds the T1D-associated *TRBV*-gene and HLA-restricted full-length CDR3β sequences that are overrepresented or depleted in the TCR repertoires, Q2 investigates the restriction of CDR3β amino acid frequencies by HLA leading to identification of HLA-associated position-specific CDR3β amino acids (CDR3 phenotypes), Q3 calculates the risk score for each repertoire on the basis of CDR3 phenotypes, and Q4 identifies the positively and negatively associated HLA motifs from CDR3 phenotypes. (**C**) We observed few TRBV-gene family– and HLA-associated CDR3β sequences depleted (blue) or overrepresented (red) in the T1D repertoires versus FDR, SDR, and CTRL in cohort 1 (Q1). (**D**) The multivariate analysis of variance (MANOVA) test *P* values from the MMLR analysis for the 1242 repertoires (cohort 1) showed association between CDR3 positions and HLA sites, where variation in CDR3β amino acid frequency at each CDR3β position was plotted for each HLA site containing mutation(s) (Q2). (**E**) A significant correlation was observed between T1D-associated HLA risk scores and CDR3 risk scores calculated for each repertoire in cohort 1 (Q3). (**F**) CDR3 risk score was also higher for the T1D clinical group and observed the expected trend where T1D > FDR > SDR > CTRL (Q3). (**G**) The positively (pHLA motif) and negatively (nHLA motif) associated HLA motif obtained from the CDR3 phenotypes, where (**H**) pHLA motif showed higher presence in T1D repertoires and (**I**) nHLA motif showed expected opposite trend with higher presence in CTRL repertoires. *P* values were described as * for [0.01, 0.05], ** for [0.001, 0.01], and *** for <0.001, and no stars plotted for nonsignificant values. AA, amino acid.

Several autoimmune diseases, including T1D, RA, CD, PSC, and SLE, have a substantial component of genetic risk driven by HLA class II genotype (*8*, *11*, *78*–*83*). Given the potential implications of high-risk HLA alleles in modulating thymic selection of autoreactive clones (*84*, *85*) or in promoting expansion of these clones in the periphery (*86*) (Fig. 3A), several computational models and statistical methods were recently developed to predict the association between the TCRs and HLA (*4*, *5*, *87*). Thus, in an attempt to control for HLA differences among clinical groups and probe for T1D-specific associations of TCRβ receptor features, we first identified strongly HLA-associated TCRβ receptor features within cohort 2 with the intent of testing the prevalence and enrichment of those features with T1D status in cohort 1 (see Q1, Fig. 3B).

We tabulated the publicity of all unique cohort 2 TCRs across the cohort 2 repertoires, defining detection as an exact or near-exact match (one CDR3β amino acid variation) and computed the odds ratio (OR) of detection in participants with and without each common HLA allele. A TCR feature was assigned to the HLA allele with the lowest *P* value (Fisher’s exact test). To limit the number of hypotheses tested in the next stage, we filtered TCR features with *P* values < 1 × 10^−8^ and detected in at least 5% of HLA-matched individuals and not more than 10% of HLA-mismatched individuals. This yielded 20,037 unique V-gene family–CDR3β centroids assigned to either MHC class I allele (*n* = 7255) or MHC class II (*n* = 12,782) allele(s) (fig. S4 and table S3). To determine whether these features were associated with T1D status, we then searched for these features in cohort 1 among participants with the hypothesized restricting HLA allele (fig. S5). After correcting for multiple hypothesis testing, 39 *HLA-DRB*1*03–associated TCRs were significantly underrepresented (false discovery rate of <0.2) among the T1D group (Fig. 3C and fig. S6, A and C). Twelve strongly HLA-associated (B*40:01, B*15:01, DQA1*01:02, DQA1*05:01, and DQB1*02:01) TCR features were also overrepresented (false discovery rate of <0.2) in the HLA-matched T1D repertoires versus controls (fig. S6B).

The TCRβ feature most underrepresented in cohort 1 T1D repertoires was *TRBV7* CASSLSLAGSNNEQFF, with it or its near-exact neighbor detected in 31.5% (80 of 254) control and 9.3% (18 of 192) T1D repertoires of persons expressing the DRB1*03:01 and/or DQA*05:01:DQB*02:01. Many of the other TCR features, strongly underrepresented in T1D repertoires (blue circles in Fig. 3C), also had a similar CDR3β sequence (one to three mutation distance from *TRBV7* CASSLSLAGSNNEQFF) as shown in motifs (fig. S6C). Two of these sequences, *V07*,CASSLSLAGTYNEQFF and *V07*,CASSLSLAGAYNEQFF, were previously identified as TCRβ sequences associated with DRB1*03:01 status by statistical testing in a healthy population (*3*). These features were observed in repertoires across all age groups among controls; however, because the populations without diabetes (FDR/SDR/CTRL) are older than the T1D-diagnosed group, we cannot exclude the possibility that the TCR features most depleted in cohort 1 T1D repertoires may reflect age-dependent acquisition of T cell memory due to recurrent exposures and vaccinations. Overall, despite identifying many TCRs associated with known T1D-associated HLA risk alleles, we observed little definitive signal of HLA-restricted TCRs enriched in repertoires on the basis of T1D clinical status after conditioning for individuals with the relevant allele.

#### Amino acid variation in HLA risk alleles restricts the position-wise amino acid frequency of the TCR repertoire

*A statistical framework to assess HLA-based TCR repertoire restriction.* Investigations into the genetic basis of T1D have increasingly highlighted the HLA region as pivotal in shaping immune tolerance and susceptibility (*85*, *88*). A previous study on 18,832 T1D case-control samples identified three–amino acid sites in *HLA-DQβ1* (site 57) and *HLA-DRβ1* (sites 13 and 71) as the main drivers of T1D risk (*79*). These three positions together explained 90% of the T1D-specific phenotypic variance in the *HLA-DRB1–HLA-DQA1–HLA-DQB1* locus and 80% of the variance explained by the entire MHC region. Ishigaki *et al.* (*7*) further observed that the polymorphisms in HLA alleles alter the T cell repertoire. The variation of amino acid in the above three HLA sites (in *DQβ1* and *DRβ1*) and associated log-odds scores were used to calculate the genetic risk of T1D from HLA in our cohorts, termed the HLA risk score (*79*) (see Methods). A TCR sequence-based disease-associated risk score (termed CDR3 risk score) was also established, which quantifies the association of high-risk HLA alleles and positional amino acid variation in CDR3β sequences (*7*). We computed CDR3 risk scores for all TCR sequences in cohort 1 containing all clinical groups and tested our observations in cohorts 2 and 3 comprising T1D and CTRL individuals, respectively, to (i) examine the association of CDR3β sequences with HLA allele-based genetic risk; (ii) identify the high-risk HLA-associated CDR3β amino acid, use them to calculate a repertoire-level CDR3 risk score, and assess whether this score exhibits an association with clinical groups; and (iii) identify CDR3β sequence motifs that encapsulate HLA-based T1D risk within TCR repertoires (see Q2 to Q4, Fig. 3B).

In keeping with previous work (*7*), we refer to amino acid locations in HLA as “sites” and amino acid locations within CDR3β as “positions.” Across eight HLA genes, we identified 398 HLA sites that exhibited amino acid variation (fig. S7A). The observed variations in the HLA sequences were distributed almost equally between HLA class I genes (218 variations, 54.8%) and HLA class II genes (180 variations, 45.2%). For the computation of a CDR3 risk score, the most frequent CDR3β lengths, 12 to 18 (denoted as L12 to L18), were considered for all HLA-CDR3β association analyses (fig. S7F). To compute the CDR3 risk score, the amino acid frequencies were calculated for each position of the CDR3β sequences, where seven–amino acid positions were present for L12 with increments of one position up to L18, due to removal of *TRBV*- and J-gene encoded regions, leading to a total of 70 CDR3β positions (fig. S7B). We divided the cohort 1 repertoires into four subsets for analysis: (i) total cohort 1 (all clinical statuses, *n* = 1242), (ii) T1D only (*n* = 402, 32.4%), (iii) FDR only (*n* = 601, 48.4%), and (iv) CTRL only (*n* = 182, 14.6%). SDRs (*n* = 4.6%) were excluded because of low sample size and absence of statistically significant T1D-associated CDR3β amino acid.

*HLA sites restrict the position-specific amino acid diversity of CDR3β sequences.* We first validated that CDR3β sequence diversity was restricted as a function of HLA sites in cohort 1, as observed in a previous study by Ishigaki *et al.* (*7*). We obtained amino acid variants for each HLA site from HLA genotyping. To quantify the association between each HLA site and CDR3β position, multivariate multiple linear regression (MMLR) was used, where a multidimensional vector of CDR3β position–specific amino acid frequency (response variable) was used to predict the association with multidimensional vector containing polymorphisms at each HLA site (explanatory variable) (fig. S7C) (*7*). To test interindividual variance in CDR3 amino acid frequencies explained by the HLA genotype, we subsequently evaluated the MMLR model using a multivariate analysis of variance (MANOVA) (Fig. 3D). A total of 27,860 MMLR-MANOVA tests were performed (398 HLA sites × 70 CDR3β positions), and 13,313 significant associations (47.8% of total tests; false discovery rate of <0.05) were observed after false discovery rate correction using the Benjamini-Hochberg adjustment (table S4). There were 5022 (37.7%) significant associations from class I HLA types and 8291 (62.3%) from class II HLA types. The number of significant associations was more than two times higher than previously reported (*7*) as our dataset was also almost double in size. The lowest *P* value was observed between *HLA-*DRB1** site 13 (*P* = 3.1 × 10^−214^), located within the T1D risk-associated peptide binding groove (*79*), and the CDR3β position 111 for L15. Position 109 in L13 (fifth position in our analysis; *P* = 4.7 × 10^−180^) was reported as the lowest *P* value in a previous study (*7*). Both CDR3β positions were in the antigen-binding region. Separately, we performed MMLR analysis on each T1D, FDR, and CTRL repertoire subset to observe similar HLA-based CDR3β restriction in each clinical group (figs. S8 and S9 and Supplementary Note). Together, (i) we confirmed the observation (*7*) that amino acid polymorphisms in HLA restrict the amino acid positional frequencies of the CDR3β sequences and (ii) extended these observations to T1D, FDR, and CTRL.

*HLA risk allele–linked CDR3 risk score is highest in individuals with T1D.* Building on observed associations between variation in HLA sites and amino acid frequencies within the CDR3β sequences, we next investigated the influence of HLA risk score on position-specific CDR3β amino acids (hereafter referred to as CDR3 phenotypes) in TCR repertoires. We first calculated the HLA risk score for each repertoire in cohort 1 (see Methods, fig. S7D, and table S5). To identify CDR3 phenotypes associated with the HLA risk score, we conducted a total of 1400 (70 CDR3β positions × 20–amino acid frequencies at each position) linear regression (LR) tests with HLA risk score (fig. S7E and table S6). There were a total of 529 CDR3 phenotypes with significant association with T1D risk for cohort 1 (*P* ≤ 0.05) (fig. S8). The correlation coefficients from these LR tests were treated as the effect sizes for the respective CDR3 phenotypes, which were further used to calculate the CDR3 risk score by summing up the effect sizes of each CDR3 phenotype for each CDR3β sequence and calculating the average value for the whole repertoire. The CDR3 risk score reflects HLA-based T1D risk in the TCR repertoire, and this relationship was validated by correlating it with the HLA risk score across different CDR3β sequence lengths [correlation coefficient (*r*) = 0.35 to 0.51, Fig. 3E]. The HLA-associated CDR3 phenotypes (amino acids) exhibit both positive and negative effect sizes. CDR3 risk scores based solely on positive or negative effect sizes showed comparable correlations (*r* = 0.27 to 0.49) to the combined CDR3 risk score (fig. S8). Additionally, average CDR3 risk score was highest for the T1D repertoires followed by FDR, SDR, and CTRL repertoires, respectively, across different lengths (Fig. 3F). A similar observation was also exhibited by classical high-risk HLA allele types, where the presence of at least one *DR3* or *DR4* allele had higher CDR3 risk score compared to other non-T1D risk allele types (*DRX/X*) (fig. S10). We further validated the robustness of CDR3 phenotypes and their effect sizes (see Supplementary Note, fig. S11, and table S7). CDR3 phenotypes from cohort 1 were also applied to cohorts 2 and 3, where T1D repertoires in cohort 2 showed significantly higher CDR3 risk scores than CTRL repertoires in cohort 3, across all CDR3β lengths (fig. S12). The significant correlation with HLA risk score and association with clinical groups were also validated for CDR3 risk score, across each subset of cohort 1 (figs. S8 and S9). To summarize, we identified 529 HLA risk score–associated CDR3 phenotypes that were used to calculate the CDR3 risk score exclusively on the basis of TCR repertoires. A robust association between CDR3 risk score, HLA risk score, and clinical groups validates the role of HLA-mediated restriction in shaping TCR repertoires (*7*).

*HLA-associated CDR3β motifs provide a simplified representation of high-risk HLA-based CDR3 restriction.* We identified a consistent recurring pattern in charged and aromatic amino acid in both positively and negatively associated CDR3 phenotypes of different CDR3β length (see Methods, fig. S8A, and table S8). The pattern was used to derive a simplified representation of the HLA-associated T1D signature within TCR repertoires as positively associated HLA motif (pHLA motif) and negatively associated HLA motif (nHLA motif) (Fig. 3G). We further used respective HLA motifs to calculate an HLA-motif score for each repertoire on the basis of the total number of CDR3β sequences containing these motifs. The pHLA-motif score was significantly higher for the T1D (0.00463 ± 0.00056, *P* < 0.001) versus FDR, SDR, and CTRL groups (values ranging from 0.00406 to 0.00430) (Fig. 3H and fig. S13). A similar trend was observed for the individuals with T1D in cohort 2 (0.00451 ± 0.00057) and CTRL in cohort 3 (0.00404 ± 0.00053). On the other hand, nHLA-motif scores were lower for T1D (0.01028 ± 0.00071) repertoires compared to those for other clinical groups (values ranging from 0.01049 to 0.01079) (Fig. 3I and fig. S14). Again, a similar trend was observed for the individuals with T1D present in cohort 2 (0.0103 ± 0.0008) and CTRL in cohort 3 (0.011 ± 0.0008).

The expected trends for pHLA-motif score (T1D > FDR > SDR > CTRL) and nHLA-motif score (T1D < FDR < SDR < CTRL) were observed, even when at least one HLA allele was non-risk (*DRX*) and when no islet autoantibody presence was detected (figs. S13 and S14). However, the differences observed for the nHLA-motif score were less pronounced. pHLA-motif score showed an expected negative correlation with T1D duration and age of individual with T1D (Pearson correlation ranging from −0.3 to −0.44), likely due to a reduction in β-cell antigens (*89*). However, the nHLA-motif score had a low and inconsistent correlation (Pearson correlation ranging from −0.12 to 0.11) with T1D duration and individual’s age. In conclusion, the distinct pHLA- and nHLA-motif scores reflect the influence of HLA-associated T1D risk on TCR repertoires, suggesting these motifs as potential markers of HLA-driven susceptibility and protection in T1D.

#### The presence of heterozygous HLA alleles may restrict TCR diversity

A recent murine study reported that the presence of heterozygous MHC class II alleles constrains the diversity of the TCR repertoire (*90*). To investigate this observation in humans, we tested the hypothesis in our dataset. First, we calculated the generation probability (*P*gen) using IGoR, a tool that models the V(D)J recombination process to estimate the likelihood of generating a specific TCR or B cell receptor sequence (*91*). *P*gen reflects the probability of a sequence being produced purely by the random recombination of V-, D-, and J-gene segments, along with nucleotide insertions and deletions. Subsequently, we calculated the postselection probability (*P*post) using soNNia, which distinguishes sequences that have undergone selection (*92*). In our study, *P*gen and *P*post were used as proxies to assess TCR repertoire diversity.

Our analysis revealed a weak trend in cohort 1, where increased HLA heterozygosity appeared to be associated with reduced TCR diversity, as indicated by both *P*gen and *P*post (fig. S15, A and B). This trend was not observed in cohorts 2 and 3, potentially because of HLA biases arising from the presence of only T1D repertoires in significantly larger cohort 2 and fewer CTRL repertoires in cohort 3, respectively. Additionally, we observed that frequencies of certain V genes (e.g., *TRBV28*, *TRBV4-3*, *TRBV32*, and *TRBV3-1*) clustered into a distinct group (fig. S15C). We used *TRBV28* as a case study and observed V-gene frequencies in three clusters: low frequency (*f* < 0.015), midrange frequency (0.015 ≤ *f* ≤ 0.04), and high frequency (*f* > 0.04). Although a direct association between V-gene frequency clusters and specific clinical groups was not identified, a potential link with certain HLA alleles was observed (fig. S15D). However, these V-gene frequency clusters could be attributed to confounding factors, such as age, ethnicity, infection history, or sequencing artifacts, which fall outside the scope of the current study. Our observations also contradict a previous study on 666 individuals, indicating HLA (class I)–heterozygous individuals present a broader immunopeptidome for recognition by cytotoxic T cells (*93*).

### ML-enabled classification of T1D status using TCR repertoires

The classification of immune repertoire for disease status prediction is based on the principle that disease signatures (identical or similar) are shared across individuals affected with the same disease (*18*). However, there are several approaches to identifying disease signatures (*94*). In our study, we used previously used HLA risk scores (*79*) and three different ML/deep learning (DL) approaches, namely, (i) public clone (V-CDR3-J)–based shallow ML, (ii) *k*-mer–based shallow ML, and (iii) attention-based DL, to classify the T1D repertoires. Across all ML approaches, T1D status was considered the positive class and the remaining clinical groups [FDR, SDR, and CTRL, together termed “no diabetes” (ND)] were considered non-T1D or negative class for training, tuning, and testing within cohort 1. The combined set of cohorts 2 and 3 (only T1D and only CTRL repertoires, respectively) was used as a held-out test set. Because of class imbalance, we used AUROC as the primary metric for evaluating predictive performance. Moreover, a confounder adjustment procedure was applied for deep repertoire classification (DeepRC) and *k*-mer–based logistic regression (LogReg) models through a series of steps aimed at adjusting the impact of age on the classification of individuals based on their T1D status (see Methods).

#### HLA-based classification serves as a strong baseline model for predicting T1D status

**Fig. 4.**
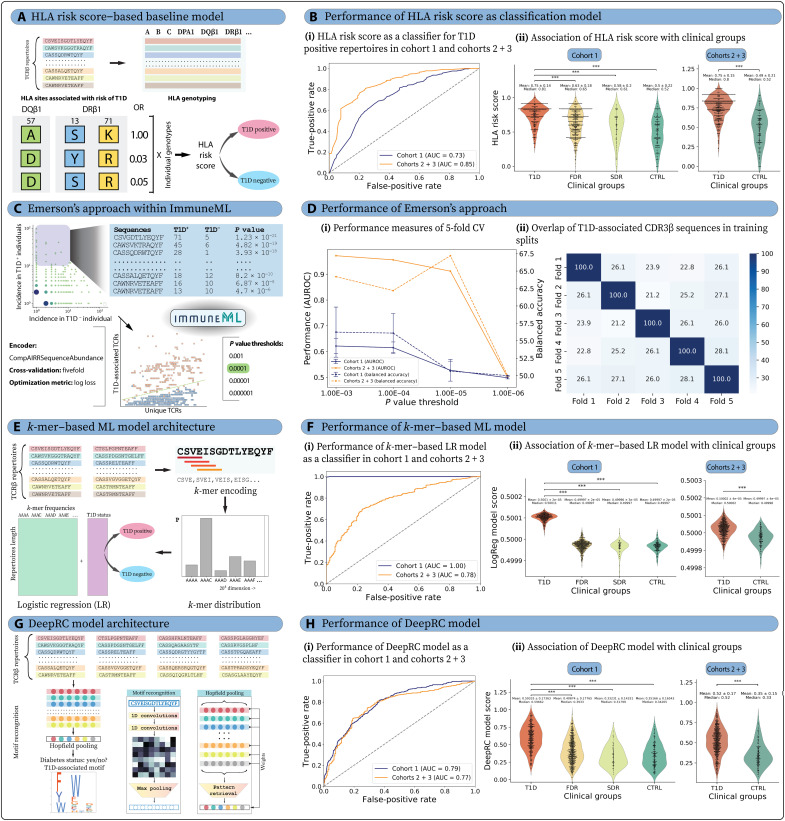
The application of different repertoire classification methods to classify T1D and non-T1D repertoires shows varying levels of prediction performance. (**A**) Schematic representation of T1D status classification using the HLA risk score (baseline model). (**B**) HLA risk score was used for classification in cohort 1 and cohorts 2 and 3, where (i) AUROC was 0.73 for cohort 1 and 0.85 for cohorts 2 and 3 and (ii) HLA risk score was higher for T1D clinical group with expected trend of T1D > FDR > SDR > CTRL. (**C**) The immuneML platform was used to replicate the approach used by Emerson *et al.* (*20*). (**D**) The model was trained on cohort 1 on the basis of log-loss calculations and fivefold CV and tested on cohorts 2 and 3, where (i) AUROC was substantially lower for cohort 1 compared to cohorts 2 and 3 for all *P* value thresholds and (ii) overlap of identified T1D-associated sequences across different splits was low (21 to 28%). (**E**) Schematic representation of repertoire classification using *k*-mer frequency as a feature in ML models. (**F**) The LogReg prediction showed overfitting in cohort 1, where (i) AUROC of cohort 1 was 1.0 and reduced to 0.775 for cohorts 2 and 3. (ii) LogReg prediction score was significantly higher in T1D, and almost no difference was observed between FDR, SDR, and CTRL in cohort 1. (**G**) Schematic representation of the DL approach (DeepRC) used for T1D disease status classification and DeepRC-motif identification. (**H**) DeepRC was more reliable compared to the LogReg model. (i) The AUROC values were similar for both cohort 1 (0.79) and cohorts 2 and 3 (0.77), and (ii) DeepRC predictions also observed the expected trend of T1D > FDR > CTRL with SDR having the least score. In all violin plots, *P* values were described as * for [0.01, 0.05], ** for [0.001, 0.01], and *** for <0.001, and no stars plotted for nonsignificant values.

We first used classical high-risk HLA alleles, *DR3* and *DR4*, to assess T1D status classification. As expected, we found that the presence of high-risk HLA alleles (*DR3* and/or *DR4*) alone is insufficient to accurately predict the clinical status of T1D (see Supplementary Note and fig. S16A), reflective of the complex, multifactorial nature of this disease (*95*). However, homozygosity for either *DR3* or *DR4* is a strong predictor of T1D status (91.4% in cohort 1 and 97.2% in cohort 2). We also used HLA risk score (*79*) to classify the T1D status (Fig. 4A). The AUROC curve was 0.73 for cohort 1 and 0.85 for the held-out test set (cohorts 2 and 3) (Fig. 4B). The maximum balanced accuracy was ~67% for both training and held-out test datasets.

We further investigated the performance of the CDR3 risk score, which was essentially an effect of high-risk HLA on the TCR repertoire. As the CDR3 risk score was calculated for different CDR3β lengths, we obtained AUROC values between 0.65 and 0.75 (maximum balanced accuracy ranging from 50.4 to 65.8%) on cohort 1 and 0.66 to 0.80 (maximum balanced accuracy ranging from 52 to 72.7%) on cohorts 2 and 3 (fig. S17). The pHLA and nHLA motifs can be considered a simplified output of a genetic association study and, therefore, tested for their classification performance. The pHLA motif obtained AUROC of 0.68 (maximum balanced accuracy of 64.8%) on cohort 1 and 0.73 (maximum balanced accuracy of 68.6%) on cohorts 2 and 3, whereas nHLA motifs expectedly had comparatively lower AUROC of 0.59 (maximum balanced accuracy of 50.2%) on cohort 1 and 0.73 (maximum balanced accuracy of 50%) on cohorts 2 and 3 (fig. S17). The performance metrics for the aforementioned classifiers are also summarized in table S9.

#### The presence of public clones is insufficient for the classification of T1D status

Emerson and colleagues (*20*) developed a statistical classification framework that could diagnose CMV status from peripheral blood TCRβ sequences. Their approach identified the statistically significant enrichment of TCRβ sequences in CMV^+^ compared to CMV^−^ individuals. We applied this approach on the T1D dataset using our open-source ImmuneML (*96*) platform (Fig. 4C). The model performance was tested on fivefold cross-validation (CV), where log-loss value was predicted to be the least at *P* value threshold of 0.0001 (log loss of 0.417 for training and 0.4 for test; fig. S18 and table S10). The maximum balanced accuracy and AUROC values of the model at the optimal *P* value threshold were low for the training cohort 1 (average AUROC of 0.615 and maximum balanced accuracy of 56.1% across five splits) (Fig. 4D). Testing the model on cohorts 2 and 3 showed relatively high AUROC (0.956) and maximum balanced accuracy (62.2%). However, increased performance in the test dataset was likely due to the sequencing depth bias between cohorts 2 and 3 because test cohort precision (100%) was notably higher than recall (24.4%) (fig. S18). A total of 140 TCR clones were predicted to be associated with T1D (table S11). A low overlap, ranging from 21 to 28%, was observed among T1D-associated TCRβ sequences across each CV split (Fig. 4D). In summary, the statistical classification approach based on public clones was insufficient to classify repertoires, due to the low prevalence of public clones.

#### k-mer–based LogReg failed to differentiate clinical groups

In the *k*-mer–based repertoire classification approach, a sliding window of 4-mers was used for each CDR3β sequence, and the occurrence of *k*-mers was calculated for each immune repertoire (see Methods). The *k*-mer frequency matrix was used as a feature in LogReg-based ML models. The final model was obtained by averaging the output of five folds into a single model after sigmoidal activation (Fig. 4E). There can be several confounding factors related to T1D affecting the TCR repertoires (*97*). The dataset was age corrected by applying sample weights to avoid more prominent age-related confounding factors (see Supplementary Note) (*98*). The LogReg model was fitted on the training dataset (cohort 1) and yielded an AUROC of 0.78 on the held-out test dataset (cohorts 2 and 3) (Fig. 4F). The model showed maximum balanced accuracy of 73.25% on the held-out test dataset with a sensitivity of 70.1% and specificity of 76.4% (table S9). We also observed that the prediction score obtained from the LogReg model did not demonstrate any association with the clinical groups, high-risk HLA types (*DR3* and/or *DR4*), or islet autoantibody status (fig. S19). Contrary to common observation, LogReg scores exhibited a positive correlation (*r* = 0.12) with T1D duration, whereas participant’s age had a negative correlation with LogReg score across all clinical groups (*r* = −0.45 to −0.5) except for T1D (*r* = 0.15). It is important to note that we observed overfitting on the training dataset even after regularizing the coefficients (see Methods). We subsequently used a more sophisticated DL model to enhance the classification accuracy of immune repertoires.

#### Interpretable DL-based multiple-instance learning achieves comparable performance to HLA-associated TCR features in differentiating clinical groups

Substantial developments have been made in applying DL methods for the classification of immune repertoires based on disease status (*20*, *36*, *99*–*101*). The DeepRC model is one such modular and customizable method particularly suited for large-scale multiple-instance learning problems, including immune repertoire classification (Fig. 4G) (*36*). The DeepRC model was trained using a fivefold CV, where three splits were respectively designated for training, one for tuning, and the remaining one for testing, in a recursive manner. The best-performing model for each CV fold was selected on the basis of the AUROC of the tuning set, resulting in five selected models. The AUROC of these five best-performing models ranged from 0.69 to 0.76 (table S9). We further trained an ensemble LogReg model to combine the predictions of the five best models, which improved the AUROC to 0.79 (maximum balanced accuracy of 72.5%). The ensemble model was considered the final DeepRC model and applied to the held-out test set, resulting in the AUROC of 0.77 (maximum balanced accuracy of 72.9%) (Fig. 4H).

The DeepRC model exhibited performance comparable to the LogReg model on the held-out test set and observed association with clinical groups, high-risk HLA types (*DR3/DR4*), autoantibody status, and age in cohort 1 (fig. S20). Notably, DeepRC predictions based solely on the TCR repertoire demonstrated improved classification performance compared to HLA-associated TCR features (such as CDR3 risk score and pHLA- and nHLA-motif scores). However, its performance was lower when compared to HLA-based genetic risk (HLA risk score). Of note, DeepRC’s predictions showed a positive correlation with T1D duration (mean = 8.03 ± 8.84 years; fig. S1) while exhibiting a negative correlation with the age of individuals with T1D. Additionally, DeepRC allowed for the extraction of a simplified sequence motif representation from the trained model, facilitating a biologically meaningful interpretation of the DL model, as discussed in the section below.

#### T1D-associated CDR3β motifs simplify the DL model with minimal performance trade-off while maintaining key trends

Interpretability of the above-presented DL model is a challenging task; however, DeepRC (*36*) supports different methods of interpretability, via the attention values and contribution analysis method known as integrated gradients (IGs) (*102*). We extracted a low-complexity DeepRC motif to identify a biological signature of T1D (see Methods) and calculated the DeepRC-motif score by normalizing the number of motif-containing sequences with the total number of CDR3β sequences. The DeepRC motif achieved an AUROC of around 0.7 on both the training dataset (maximum balanced accuracy of 65.67%) and held-out test dataset (maximum balanced accuracy of 66.48%) (table S9). The DeepRC motif exhibited trends analogous to those of the DeepRC model; however, T1D duration showed a modest negative correlation (*r* = −0.13) with the DeepRC-motif score (fig. S21). Together, DeepRC provided a low-complexity alternative in the form of a motif, which demonstrated an association with clinical status, despite being derived solely from TCR repertoires and without HLA information. It is noteworthy that the DeepRC motif is a gapped motif (*103*, *104*), which may exhibit a higher rate of false positives in identifying T1D-associated CDR3β sequences, as ~17% of the CDR3β sequences in a repertoire contained a DeepRC motif, compared to only ~0.4% for the pHLA motif.

The identification of pHLA motifs by aggregating position-specific amino acids from CDR3β sequences of varying lengths presents considerable challenges. Nevertheless, only ~0.4% of the repertoires contained these motifs. To enhance specificity, we obtained refined consensus HLA-associated CDR3β sequences by identifying the CDR3β sequences that contain both pHLA motif and DeepRC motif, termed “consensus motif.” This approach reduced the proportion of HLA-associated CDR3β sequences to ~0.25%, without reducing the performance compared to pHLA motifs. Additionally, the AUROC of the consensus motif (0.74) was marginally improved compared to that of the pHLA-motif (0.73) (fig. S22). Expectedly, the consensus-motif score was higher in T1D donors and increased alongside the number of autoantibodies and risk HLA (fig. S22). It also showed a negative correlation with both age of the individual with T1D and T1D duration (fig. S22). Together, the consensus motif provides moderate accuracy for classifying T1D status based on peripheral blood CDR3β sequences.

### T1D-associated TCR motifs as predictive markers in independent cohorts

#### T1D genetic risk loci are associated with TCR motifs in peripheral blood and pancreatic lymph nodes

Despite substantial progress in defining genetic loci that contribute to T1D risk via genome-wide association studies (*105*), our understanding of the impact of such variants on immune function remains limited. We have shown that high-risk HLA alleles restrict the TCR repertoires, allowing for potentially autoreactive TCR presence in genetically predisposed individuals (Fig. 3). Thus, we hypothesized that T1D risk variants, particularly in the HLA region, may be associated with increased frequency of the pHLA motif and DeepRC motif. To address this, we performed microarray-based precision medicine genotyping (*45*) of 716 unrelated living participants from cohort 1 (ND, *n* = 489; and T1D, *n* = 227).

**Fig. 5.**
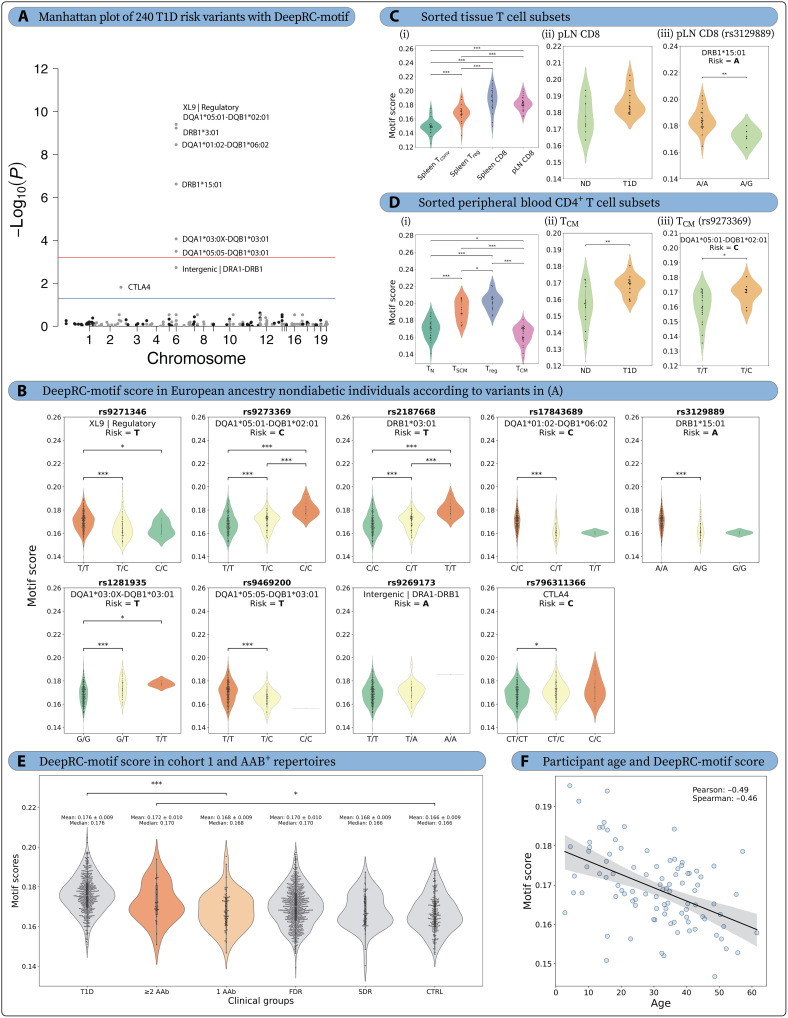
DeepRC-motifs are enriched in carriers of T1D risk genetics in bulk and sorted central memory CD4^+^ T cells from peripheral blood, pancreatic lymph node CD8^+^ T cells, as well as autoantibody-positive, nondiabetic (AAB^+^) peripheral blood repertoires. (**A**) Manhattan plot of 240 T1D risk variants versus DeepRC-motif score in bulk peripheral blood. LR assuming additive genotypic effect with age, sex, T1D status, predicted probability of CMV infection, and 10 multidimensional scaling components as covariates. Benjamini-Hochberg false discovery rate threshold at *P* = 5.73 × 10^−4^ (red line) and a conventional threshold of *P* = 0.05 (blue line). (**B**) Violin plots of DeepRC-motif score in European ancestry nondiabetic (ND) individuals, according to variants in (A). (**C**) DeepRC-motif score across sorted T cell subsets in spleen and pancreatic lymph node (pLN). (i) Mixed-effects analysis with Tukey’s multiple comparisons test. (ii) pLN CD8^+^ T cell DeepRC-motif score according to T1D status. (iii) pLN CD8^+^ T cell DeepRC-motif score according to DRB1*15:01 tag SNP. (**D**) DeepRC-motif score in sorted peripheral blood CD4^+^ T cell subsets. (i) Mixed-effects analysis with Tukey’s multiple comparisons test. (ii) Central memory CD4^+^ T cell DeepRC-motif score according to T1D status. (iii) Central memory CD4^+^ T cell DeepRC-motif score according to DQA1*05:01-DQB1*02:01 tag SNP. (**E**) AAb^+^ individuals (*n* = 95) were stratified by autoantibody count: single autoantibody (“1 AAb,” *n* = 65) and multiple autoantibodies (“≥2 AAb,” *n* = 30). Comparison of DeepRC-motif scores for the 1 AAb and ≥2 AAb subgroups against all other clinical groups in cohort 1. (**F**) Scatter Plot demonstrating a strong negative correlation between DeepRC-motif score and participant age in the AAb^+^ group of cohort 1. In all violin plots, *P* values were described as * for [0.01, 0.05], ** for [0.001, 0.01], and *** for <0.001, and no stars plotted for nonsignificant values. T_N_, naive T cell; T_SCM_, stem cell memory T cell; T_reg_, regulatory T cell; T_conv_, conventional T cell; T_CM_, central memory T cell.

To investigate which T1D risk variants contributed to the association with the enriched TCRβ motifs, we performed quantitative trait locus (QTL) analysis. HLA-DQA1*05:01-DQB1*02:01 (*P* = 3.91 × 10^−10^), HLA-DRB1*0301 (*P* = 5.83 × 10^−10^), HLA-DQA1*01:02-DQB1*06:02 (*P* = 3.47 × 10^−9^), HLA-DRB1*15:01 (*P* = 2.38 × 10^−7^), HLA-DQA1*03:0X-DQB1*03:01 (*P* = 8.5 × 10^−5^), and HLA-DQA1*05:05-DQB1*03:01 (*P* = 3.23 × 10^−4^) haplotypes were significantly associated with DeepRC-motif frequency independent of disease status such that risk haplotype correlated with an increased motif score (Fig. 5, A and B). The risk alleles of a T1D-associated variant tagging the *XL9* super enhancer (*P* = 3.91 × 10^−10^), known to regulate *HLA-DRB1* and *HLA-DQA1* expression (*106*), and an intergenic *HLA-DRA1-DRB1* variant (*P* = 1.85 × 10^−3^) were likewise associated with increased peripheral blood DeepRC-motif score (Fig. 5, A and B). An intergenic deletion nearby *CTLA4* carrying risk for T1D that is in linkage with reduced *CTLA4* expression QTL (eQTL) (*107*, *108*) was also weakly associated with increased DeepRC-motif score (*P* = 0.015) (Fig. 5, A and B). Analysis of the pHLA-motif revealed enrichment in risk allele-carrying individuals for HLA-DRB1*0301 (*P* = 0.018), HLA-DQA1*05:01-DQB1*02:01 (*P* = 0.018), and HLA-DQA1*05:05-DQB1*03:01 (*P* = 0.021) (fig. S23, A and B) similar to that observed for the DeepRC motif. The nHLA-motif frequency was decreased in those with the T1D risk haplotypes HLA-DQA1*02:01-DQB1*02:02 (*P* = 9.14 × 10^−8^), HLA-DQA1*03:0X-DQB1*03:02 (*P* = 9.85 × 10^−6^), and *HLA-DR4* (*P* = 1.1 × 10^−5^) (fig. S24, A and B). Thus, variants affecting HLA class II type in addition to HLA class II and *CTLA4* expression levels may influence the frequency of T1D-enriched TCRβ motifs in blood.

Observations in peripheral blood were tested in TCRβ-sequenced sorted conventional CD4^+^CD127^+^ T (T_conv_) cells, CD4^+^CD127^−^CD25^+^ regulatory T (T_reg_) cells, and CD8^+^ T cells from pancreatic lymph nodes (pLNs) as well as spleen from 27 Network for Pancreatic Organ donors with Diabetes (nPOD) donors [data previously published (*42*): ND, *n* = 7; T1D, *n* = 15; T2D, *n* = 3; and other diabetes, *n* = 2] to understand matters of cell type and tissue specificity. DeepRC- and pHLA-motif scores were significantly increased in CD8^+^ T cells in the spleen and pLN as compared to T_conv_ and T_reg_ cells in the spleen in all participants (Fig. 5C and fig. S23C). T_reg_ cell DeepRC- and pHLA-motif scores were also higher than those of T_conv_ cells in the spleen (Fig. 5C and fig. S23C). nHLA-motif score was enriched in splenic T_conv_ and T_reg_ cells as compared to that in spleen and pLN CD8^+^ T cells (fig. S24C). Individuals with T1D showed trends for increased DeepRC-motif (*P* = 0.095, Fig. 5C) and pHLA-motif (*P* = 0.067, fig. S23C) cores and increased nHLA-motif (*P* = 0.025, fig. S24C) score in pLN CD8^+^ T cells as compared to those with ND. Similar to the peripheral blood findings, HLA-DRB1*15:01 (*P* = 0.004) was also significantly associated with decreased DeepRC-motif frequency in pLN CD8^+^ T cells. nHLA-motif score was decreased in HLA-DQA1*02:01-DQB1*02:02 pLN CD8^+^ T cells (*P* = 0.226, fig. S24C), in contrast to the increased frequency in peripheral blood (fig. S24A). These data support the notion that HLA class II–mediated protection from T1D may limit the frequency of potentially autoreactive TCRβ motifs at the site of autoimmunity.

Similar analysis was performed in TCRβ-sequenced sorted CD45RO^−^CD27^+^CCR7^+^CD95^−^ naive (T_N_), CD45RO^−^CD27^+^CCR7^+^CD95^+^ stem cell memory (T_SCM_), CD25^hi^CD127^lo/−^ T_reg_, and CD45RO^+^CD27^+^ central memory (T_CM_) CD4^+^ T cell subsets from peripheral blood of 28 living study participants [data previously published (*41*): ND, *n* = 14; and T1D, *n* = 14]. DeepRC- and pHLA-motif scores were significantly higher in T_SCM_ and T_reg_ cells than those in T_N_ and T_CM_ cells (Fig. 5D and fig. S23D). nHLA-motif score was increased in T_reg_ cell as compared to that in T_N_ and T_CM_ cells, while T_SCM_ cell also showed higher frequency than T_CM_ cell (fig. S24D). Despite showing a lower DeepRC-motif score compared to other cell subsets, T_CM_ cell DeepRC-motif score was significantly enriched in T1D as compared to that in ND individuals (*P* = 0.0047, Fig. 5D), which can partially be accounted for by increased motif score in HLA-DQA1*05:01-DQB1*02:01–carrying individuals (*P* = 0.028, Fig. 5D). HLA-DQA1*05:01-DQB1*02:01 was also associated with enrichment of the pHLA-motif score in T_reg_ (*P* = 0.031) and T_CM_ (*P* = 0.027) cells (fig. S23D). Together, these data suggest that T1D HLA risk genetics may confer selection pressure for T1D-enriched TCRβ motifs in peripheral blood, particularly in CD4^+^ T_CM_ and T_reg_ cells.

#### Islet autoantibody–positive individuals without clinical T1D exhibit intermediate motif scores between T1D and control repertoires

To investigate whether T1D-associated TCR motifs arise before clinical disease onset, we examined the 95 autoantibody-positive, nondiabetic (AAb^+^) repertoires from cohort 1 that had not been included in prior analyses. This dataset includes 65 (68.4%) individuals with a single autoantibody (1 AAb) and 30 (31.6%) individuals with two or more autoantibodies (≥2 AAb; *n* = 17 with two, *n* = 9 with three, and *n* = 4 with four autoantibodies), the latter of which represents preclinical stage 1 to 2 T1D (*109*).

The frequencies of all three motifs (DeepRC, pHLA, and nHLA) in the AAb^+^ repertoires were intermediate between T1D and control groups of cohort 1, following the expected trends: T1D-associated DeepRC (Fig. 5E) and pHLA (fig. S25A) motifs were highest in the T1D group, whereas the protective nHLA motif was most abundant in controls (fig. S25C). Notably, DeepRC- and pHLA-motif scores also showed strong negative correlations with age (Pearson’s *r* = −0.49 and −0.50, respectively; Fig. 5F and fig. S25B), indicating that, despite the presence of islet autoimmunity, older AAb^+^ individuals do not exhibit elevated T1D-risk TCR signatures, consistent with the notion of a more aggressive disease pathogenesis in younger individuals (*110*–*112*). In contrast, the protective nHLA-motif displayed a weak positive correlation with age among AAb^+^ persons (*r* = 0.12, fig. S25D). These observations support the hypothesis that T1D-associated TCR signatures emerge in the TCR repertoire before clinical onset and coincide with the emergence of autoantibodies in affected individuals.

## DISCUSSION

The adaptive immune repertoire is shaped by a myriad of factors, including aging, environmental exposures, and genetics. T1D natural history studies identified islet autoantibody seropositivity as early autoimmune signals in genetically predisposed populations (*113*, *114*). Yet there is a need to improve disease prediction and monitoring through the validation of additional cellular biomarkers. It has been proposed that the TCR repertoire, as a record of response to foreign and self-antigens, could potentially distinguish a T1D-specific signal from repertoire shifts caused by pathogen exposures and vaccinations. In this study, we have comprehensively assessed the HLA-based TCR repertoire restriction, TCR repertoire classification, and immune signal identification on a cross-sectional dataset of 2250 TCR repertoires spanning various stages through the natural history of T1D. We initially observed that repertoire-level diversity, similarity, and V-gene usage do not differentiate T1D donors from their relatives without diabetes or unrelated controls. However, mapping known T1D antigen–specific clones (*63*) in our dataset revealed an enrichment of T1D-associated sequences in T1D repertoires. This finding aligns with recent data that demonstrated the stable enrichment of CDR3β sequences matching known preproinsulin-reactive clones during disease progression in a genetically at-risk cohort, thereby providing support for T1D-reactive sequences as a biomarker in T1D (*40*). Despite this, we also found T1D-associated sequences frequent in non-T1D donors. This is in accordance with previous reports suggesting phenotypic rather than frequency differences in circulating autoreactive cells in T1D (*115*) and may also be affected by an incomplete understanding of antigen reactivities and receptor sequences in T1D, altogether complicating the utility of a priori sequences and necessitating a more comprehensive analysis of our bulk repertoire data.

We next examined how high-risk HLA molecules, which account for most of the genetic risk in T1D (*11*, *79*), influence T1D-associated TCR features. Clustering of public TCRs yielded more significantly underrepresented hits associated with HLA risk allele HLA-DRB1*0301 in T1D, while we identified overrepresented TCR features associated with risk *HLA-DQ* molecules DQA1*0501 and DQB1*0201 in T1D. The observation of a predominance of statistically significant underrepresented features is intriguing given previous data suggesting that persistent low-grade viral infections are associated with autoimmunity development (*116*) or that certain infections may be protective (*74*, *117*). Alternatively, our observations could reflect deficits in regulatory subsets in T1D; however, while differences in diversity have been shown for other circulating T cell subsets in T1D, this is not the case for T_reg_ cells (*41*). Thus, further investigation of the identity and phenotypes of T cells expressing these differentially abundant sequences is warranted.

We further analyzed the *HLA-DQβ1* and *HLA-DRβ1* variants associated with high risk for T1D (*79*) to examine their effect on TCR repertoire restriction (*7*). We identified several TCR features, including a repertoire-level CDR3 risk score and subsequence-level CDR3 phenotypes, which indicate HLA-based T1D susceptibility imparted solely by TCR repertoires. Additionally, we identified HLA-associated CDR3β motifs by aggregating CDR3 phenotypes, which were positively (pHLA) and negatively (nHLA) associated with T1D. pHLA motifs were enriched in T1D donors and exhibited an increased frequency of aromatic, negatively charged and hydrophobic residues, particularly in the CDR3β middle and C terminal region, a feature of self-reactivity (*83*, *118*, *119*). Consensus-motif scores (which contain both the pHLA motif and DeepRC motif) exhibited a negative correlation with disease duration and age of the participant, potentially indicative of a waning autoimmune response associated with concomitant loss of β cell antigens (*89*). To test the potential for HLA alleles to influence the diversity of the TCR repertoire, we modeled T cell selection by calculating the probability of generation and selection of TCR sequences. In accordance with a recent murine study (*90*), we observed, although weakly, that heterozygosity limits repertoire diversity. This runs contrary to some observations that the highest risk HLA *DQ2*/*DQ8* genotype allows for transdimer formation, thus potentially allowing for presentation of a more diverse pool of antigens (*120*) or permitting cross-reactivity (*121*). Moreover, recent work suggests that HLA-DM mediated editing is reduced for *HLA-DQ2* and *HLA-DQ8* pMHC molecules (*122*). While it is thought that the lack of DM editing would favor presentation of peptides with more rapid dissociation rates (*123*) and thus allow for broader peptide presentation, it is still unclear how lower versus higher stability of pMHC complexes affects the diversity of the repertoire (*124*). These analyses exemplify the multifaceted influence of HLA and the complexity of immune regulation in T1D while providing further insights into the interplay between genetic risk and TCR repertoire diversity.

Considering that most prior analyses have focused on HLA as a primary determinant of T1D risk, we applied several ML strategies to identify features indicative of T1D status independent of HLA information. Although the performance of HLA-dependent TCR features (pHLA and nHLA motifs; AUROC of ~0.73) was lower than the HLA risk score (AUROC of 0.85), the classification of T1D status without explicitly utilizing HLA allele information achieved notable performance with the *k*-mer–based LogReg strategy (AUROC of 0.78) and DeepRC (AUROC of 0.77). While the *k*-mer–based LogReg model exhibited overfitting on the training dataset, the DeepRC model proved more interpretable and capable of identifying the input sequence with the highest contribution to the T1D prediction per repertoire. Sequences identified from the DeepRC model were subsequently analyzed to derive a T1D-associated motif. We found that motif scores were increased in T1D donors and correlated with the number of islet autoantibodies and risk HLA, collectively providing support for the DeepRC motif as being involved in T1D pathogenesis. Similar to our pHLA motif, we once again found a negative association of DeepRC-motif score with T1D duration. A caveat for our analysis is the potential for false positives and nonspecific sequences included in our ML motif. The frequency of CDR3β sequences containing the DeepRC motif was ~17%, whereas the estimated precursor frequency of T1D antigen–specific T cells is around 1 to 10 per million cells (*125*), closer to the frequency of sequences having the pHLA motif. The DeepRC motif was identified without incorporating HLA information and may, therefore, capture TCR sequences not restricted by high-risk HLA alleles. These motifs could reflect cross-reactivity (*126*), bystander activation, or other non–HLA-restricted immune features, offering modest but potentially complementary insights beyond HLA-restricted TCRs that may be relevant to the progression of T1D. Thus, further investigation is needed to examine the epitope reactivity of sequences containing this motif. Moreover, we expect incorporation of additional information such as paired TCRαβ sequences and cell phenotype features (e.g., activated effector memory states) to further improve classification accuracy.

We found T1D risk alleles, primarily within the HLA region, to control the frequency of the DeepRC motif. Notably, risk genotypes at *HLA-DQ* and *HLA-DR* loci, as well as regulatory element *XL9*, were associated with a higher motif score. We recently reported that high-risk HLA alleles contributed to increased HLA expression on circulating monocytes (*127*). Our motif score eQTL (*107*) results provide further support for the notion that enhanced HLA class II expression modulated by risk alleles could promote the activation and expansion of lower-affinity autoreactive clones in T1D. The only eQTL hit outside of the HLA region passing the significance threshold was located within *CTLA4* and was previously linked with reduced CTLA4 expression (*128*). Notably, other T1D risk-conferring variants within *CTLA4* are known to negatively affect *CTLA4* expression and function (*128*) and thus are thought to result in reduced TCR activation threshold or impaired regulatory signaling.

*HLA-DQ*–restricted proinsulin and HLA-DR–restricted GAD-specific T cells have been identified within the insulitic lesion (*129*) or peripheral blood (*43*) as well as the pLN and spleen of T1D donors (*42*). To explore the tissue relevance of the identified DeepRC motif and the cell-type localization of motif-bearing sequences, we interrogated the motif in our previously published sorted bulk-sequenced nPOD tissue dataset (*42*). We found that motif-bearing sequences were increased in CD8^+^ T cells and T_reg_ cells in the spleen and pLN, with a higher motif score in pLN CD8^+^ T cells from T1D donors, which were modulated by genotype at protective HLA class II loci. The observation of HLA class II loci modulating CD8^+^ T cell phenotype likely indicates an indirect effect, whereby risk alleles promote inflammatory CD4^+^ T cell phenotype or alter T_reg_ cell phenotype to permit this enrichment (*130*), although the mechanisms controlling this need further defining. Accordingly, in a separate validation cohort (*41*) consisting of a sorted CD4^+^ T cell subset from the PBMCs of T1D and CTRL individuals, we identified an enrichment in motif score among CD4^+^ T_CM_ cells in T1D donors, which was affected by *HLA-DQ* risk genotype. CD4^+^ T_CM_ cells have the capacity to produce cytokines such as interleukin-2 (IL-2) and IL-21, which drive CD8^+^ T cell proliferation and effector phenotype (*131*). Notably, alterations in the peripheral blood CD4^+^ T_CM_ cell compartment, specifically involving increases in T follicular helper (T_FH_) cells, have been reported in T1D and before overt disease (*132*, *133*). An altered T_FH_-like signature is detectable as early as infancy in genetically at-risk children who later progressed to T1D (*134*), implicating genetics as a driving factor in enhancing helper and effector T cell function in T1D. Thus, we have identified a replicable TCRβ motif that encodes T1D status, is relevant in circulation as well as disease relevant tissue, and is controlled by T1D risk loci.

One key aspect of our study is that HLA-based TCR restriction analysis did not account for clinical group information, while FDR and SDR repertoires were classified as ND in the ML models. Nevertheless, most analyses, including the DeepRC model, HLA and CDR3 risk scores, and DeepRC- and pHLA-motif scores, revealed a trend where the scores were highest for T1D repertoires and lowest for CTRL, following the expected pattern: T1D > FDR > CTRL. Furthermore, both the DeepRC motif and pHLA motif were overrepresented in individuals with genetic risk for T1D across several independent cohorts, including bulk and sorted CD4^+^ T_CM_ cells from peripheral blood as well as pLN CD8^+^ T cells. The AAb^+^ peripheral blood repertoires displayed intermediate frequencies of all three motifs (DeepRC, pHLA, and nHLA) between T1D and control groups, again following the expected trends of T1D > AAb^+^ > CTRL for DeepRC and pHLA motifs and CTRL > AAb^+^ > T1D for the nHLA motif.

Notably, we observed a limited presence of public TCRs in our dataset compared to those in previous studies focused on other autoimmune diseases (*20*, *40*, *135*). This discrepancy may be attributed to high variability in confounding factors, including age, T1D duration, genetic background, and ethnicity of the individuals. A large longitudinal HLA-stratified cohort could potentially enhance the identification of T1D-specific public TCRs. Moreover, as the number of studies examining TCR repertoire in T1D increases, ongoing efforts to curate repertoire data from donors at varying risk for T1D (*15*) will facilitate the analysis of TCR groups shared across large numbers of donors and shaped by ongoing immune pressures, such as pathogen exposure and autoimmunity (*76*, *136*). Identifying groups of public T1D-associated TCRs would present opportunities for disease monitoring and the development of targeted therapeutics. In this study, we integrated two complementary assessments: (i) the influence of genetic risk on TCR repertoires with HLA-based restriction analysis and (ii) influences of T1D-associated genetic and environmental factors (*137*) on TCR repertoires through ML-based methodologies. This integrative framework underscores the central role of HLA in T1D susceptibility and highlights TCR motifs associated with disease risk.

### Limitations of this study

As with all AIRR studies, sequencing depth remains a critical concern, particularly for autoimmune diseases such as T1D, where immune signals in peripheral blood may be relatively low (*25*, *26*). While the repertoires in the current study maintain an average of unique CDR3β sequence count of >100,000 (cohorts 1 and 2), deep sequencing in cohort 3 yielded nearly a 3.5-fold increase in unique CDR3β sequences. Although analysis of replicates demonstrated a high MH index and strong Pearson correlation between deep and shallow sequencing data, the loss of additional unique CDR3β sequences at lower sequencing depth may affect the identification of rare, T1D-specific signals. We have made efforts to mitigate the effects of potential confounding factors, including differences in sequencing depth/protocol and age across the three cohorts evaluated herein. However, we acknowledge that other variables (e.g., sex, demographics, clone count, and prior infections) may still affect the generalizability of our models (*16*). Additionally, mitigating overfitting in future studies would also require larger datasets and improved regularization schemes for models to ensure that learnt signals more strictly reflect the underlying phenotypes of interest, rather than a combination of desired and distracting signals. Furthermore, repetition in data collection centers introduced duplicate repertoires with different donor identification numbers into the dataset. While we applied a stringent MH similarity threshold to exclude duplicates, there remains a possibility that some duplicate repertoires were retained in the dataset.

Islet autoantibody seroconversion peaks early in childhood, at around 2 years of age (*138*). As this represents a critical period of exposure to pathogens and vaccinations, it is extremely important to understand the longitudinal stability of the TCR repertoires in children to effectively identify TCR-based signatures and monitor their shifts over time in diseases such as T1D. A more extensive longitudinal sampling could also provide insights into how other diseases or infections influence the TCR repertoire at specific time points. Additionally, a larger cohort of HLA-matched individuals would be ideal for identifying HLA-specific signals associated with T1D. While we were not able to effectively classify T1D using a small number of public clones in our dataset, it is possible that a dataset comprising multiple autoantibody-positive (in stage 2 and recent-onset stage 3) individuals with age and HLA-matched controls may allow extraction of disease signals.

The HLA risk score is derived from three HLA positions with the strongest association with T1D risk (*79*). A more comprehensive HLA risk score incorporating additional HLA positions could improve T1D risk assessment. HLA risk score is fixed and thus cannot serve as a biomarker for T1D disease progression, diagnosis, or assessing the effects of T1D treatment. It is also essential to acknowledge the limitations associated with derivation of HLA motifs. HLA motifs were constructed by aggregating position-specific amino acid of different CDR3β lengths, which complicates the interpretation of positional dependence. Furthermore, motifs derived from aggregated positional information do not permit the retrieval of amino acid–specific positional dependencies and are primarily suited to produce ungapped motifs. Similarly, the DeepRC-derived motifs had higher proportions within each repertoire, suggesting a substantial rate of false-positive T1D-associated CDR3β sequences and emphasizing the need for more effective motif extraction methods (*139*). A comprehensive study is also warranted to examine the role of underrepresented protective motifs within the T1D cohort, potentially enhancing T1D risk prediction.

The identification of disease-associated signals represents an inherent challenge in T1D research and reflects the complexity of the disease rather than a limitation of the current analysis. A future strategy toward identifying disease-relevant biomarkers may benefit from combining PBMC-based sorting with tissue-based, spatially resolved, and cell population–specific sampling. That being said, we showed that the T1D-associated TCR signals identified were also present in entirely different datasets (*39*, *140*), tracking with HLA risk status. Additionally, T1D antigen–specific TCRs were also observed in CTRL individuals, warranting further studies on low-affinity, cross-reactive signals and physiological autoreactive processes (*141*). Biomarker specificity could be further enhanced by incorporating paired alpha and β chain information (*142*). Future studies aimed at identifying disease-specific antigens recognized by T1D-associated TCRs through experimental functional validation methods, such as tetramer staining or antigen-specific activation assays, could provide valuable insights into the underlying disease mechanisms and further refine disease-associated TCR motifs. To circumvent large-scale experimental screening for antigen specificity (which is now unfeasible), there is also a need for artificial intelligence–based deorphanization of TCR epitopes (*23*, *143*–*148*).

There is growing interest in harnessing the immune history encoded within TCR repertoires to develop TCR-based diagnostics and therapeutics for future clinical applications, including immunization (*149*, *150*), viral infections (*20*, *151*), and autoimmune diseases (*2*, *152*). We conducted a large-scale, comprehensive analysis of T1D-associated TCR repertoire alterations by immunosequencing the CDR3β regions and genotyping HLA using sampling amenable to young individuals for screening efforts. The HLA-associated T1D risk was leveraged to extract robust HLA-mediated TCR features, including repertoire-level CDR3 risk score and CDR3β subsequence-level HLA motifs, to classify T1D status. Additionally, we predicted T1D status directly from TCR repertoires using ML and DL approaches. DeepRC performed comparably to HLA-mediated TCR features and identified a simplified DeepRC motif from TCR repertoires alone, without needing HLA information. These potential motif-based biomarkers, derived from cross-sectional samples, reflect the natural history and pathogenesis of T1D, and show associations with HLA, disease duration, and islet autoantibody status. These findings not only support future longitudinal studies but also introduce disease-associated motifs that can be tracked in response to clinical interventions aimed at halting disease progression, such as T cell–targeting therapies like teplizumab (anti-CD3, Tzield), anti-thymocyte globulin, alefacept [LFA3–immunoglobulin (Ig) fusion protein], and abatacept (*CTLA4*-Ig fusion protein) (*153*). As studies expand in organ donor tissues from individuals with T1D, this work is expected to provide a valuable reference dataset for identifying antigen reactivities and convergent or public receptor sequences enriched in the pLN and pancreas.

## METHODS

### Study participants and sample collection

Study participants or their legal guardians provided written informed consent, with pediatric and adolescent participants also providing assent before enrollment. Cohort 1 comprised individuals from outpatient clinics at the University of Florida (UF; Gainesville, FL), Nemours Children’s Hospital (Orlando, FL), and Emory University in accordance with Institutional Review Board (IRB)–approved protocols at each site. All samples and their associated data and metadata were deidentified in accordance with UF IRB201400703. Peripheral blood samples were collected into sodium heparin–coated and serum separator vacutainer tubes by venipuncture from nonfasted individuals (i.e., unknown prandial state or time of day) and then shipped or rested overnight before processing at the UFDI. At the time of blood draw, participants were generally healthy with no reported malignancy or infection, and sample collection occurred between 2010 and 2018 before the COVID-19 pandemic. Cohort 2 included individuals with T1D from the T1D Exchange Clinical Network (*154*, *155*), and cohort 3 included controls sequenced by Adaptive Biotechnologies.

### DNA isolation and TCRβ sequencing

Genomic DNA (gDNA) was isolated from PBMCs of 2250 individuals. *TRB* (TCRβ) CDR3 region sequencing was performed via Adaptive Biotechnologies immunosequencing assay. Briefly, gDNA was amplified using bias-controlled multiplex PCR before sequencing (*156*, *157*). Cohort 1, containing 1393 repertoires, comprising 188 CTRL, 59 SDR, 625 FDR, and 426 T1D repertoires, was sequenced using hsTCRB_v4 Service at shallow sequencing depth (*156*, *157*). There were 95 additional autoantibody-positive non-T1D repertoires (AAb^+^) in cohort 1, which were excluded to avoid potential bias in the study. Cohort 2, containing 679 T1D repertoires, was sequenced using hsTCRB_v4b Service at shallow depth. Cohort 3, containing 178 CTRL repertoires, was sequenced using hsTCRB_v4b Service at deep sequencing depth. The shallow and deep technical replicates of cohort 1 were studied separately and were not part of the abovementioned cohort.

### Genotyping

Individuals from cohorts 1 (*n* = 1242), 2 (*n* = 645), and 3 (*n* = 178) were genotyped using the UFDIchip custom microarray, with processing on an Affymetrix GeneTitan instrument and a BioMek FX dual arm robotic workstation (*45*). The UFDIchip includes >9000 markers from the Axiom Precision Medicine Research Array (Thermo Fisher Scientific) covering the HLA region. Raw data were converted to genotype calls using Axiom Analysis Suite software (v3.0, Thermo Fisher Scientific) “Best Practices Workflow” with “Human.legacy.v5” settings. Four-digit HLA genotypes were imputed using Axiom HLA Analysis software (v1.2.0.38) (*158*). Imputation results were used for analysis if probability scores of >0.7. HLA class II haplotypes considered to carry T1D risk were defined as DR3 (HLA-DRB1*03:01–HLA-DQA1*05:01–HLA-DQB1*02:01) and *DR4* (HLA-DRB1*04:01/02/04/05/08–HLA-DQA1*03:01–HLA-DQB1*03:02/04) (*11*). Individuals were grouped on the basis of HLA class II risk diplotypes, with non-risk haplotypes designated as “*DRX*,” resulting in the following groups: *DRX/X*, *DR3/X*, *DR3/3*, *DR4/X*, *DR4/4*, or *DR3/4*. SNP2HLA (v1.0.3) was used for HLA amino acid polymorphism imputation from genotyping data (*47*)*.*

### Islet autoantibody measurement

GADA, IA-2A, and ZnT8A were measured in serum using commercial enzyme-linked immunosorbent assay kits, which have consistently demonstrated high sensitivity and specificity in the Islet Autoantibody Standardization Program (IASP) workshops (*159*). IAA was measured from serum by chemiluminescence assay, as recently reported and evaluated by the IASP (*160*).

### Dataset preprocessing

First, we filtered the out-of-frame sequences from each repertoire and selected repertoire, which contains a minimum of 50,000 distinct sequences. In this study, we defined a clone or clonotype as a unique CDR3β sequence, unless specified otherwise. The clone counts for duplicate CDR3β amino acid sequences were merged. Additionally, V/D/J-gene information from different columns was consolidated to address unresolved V/D/J-gene annotations. Repertoires with a potential common donor origin were excluded by applying an MH similarity threshold of less than 0.40, based on thresholds established in previous studies on replicate repertoires (*161*, *162*).

### Statistical analysis

All cluster heatmaps were generated using the Unweighted Pair Group Method with Arithmetic mean (UPGMA) clustering method and Euclidean distance matrix (*163*). The *P* values for the multiple testing were performed using the Kruskal-Wallis test. Similarly, *P* values for pairwise testing were calculated using two tailed Mann-Whitney *U* tests. *P* values were described as * for [0.01, 0.05], ** for [0.001, 0.01], and *** for <0.001, and no stars plotted for nonsignificant values. All *P* values were adjusted for multiple testing using the Benjamini-Hochberg method.

### Repertoire-level similarity and diversity analysis

#### V-gene usage analysis

The V-gene distribution within each immune repertoire was determined by assessing the frequency of occurrence for each V gene. This frequency was obtained by dividing the count of a specific V gene by the total number of unique clones present in the repertoire. The V-gene frequencies were calculated without considering the clonal frequency.

#### Hill-based diversity profile analysis

The diversity of the TCR repertoires was calculated using Hill-based diversity profile, which is based on Rényi’s definition of generalized entropy (*164*–*166*). It is defined as

$${\;}^{\alpha }D\left(f\right)={\left(\sum_{i=1}^{n}f_{i}^{\alpha }\right)}^{\frac{1}{1-\alpha }}$$ (1)

where f is the clonal frequency distribution, with $f_{i}$ being the frequency of each clone and n being the total number of clones. The α values represent weights, which means that, as α increases, higher frequency clones are weighted more. The α-parameterized diversity generates a diversity index profile for a given array of α values. The diversity profile is not defined for α = 1. However, diversity tends toward Shannon entropy when α tends to 1 (based on L’Hospital’s rule). We calculated the diversity profile of each repertoire on the basis of α value ranging [1, 10] with step size of 0.2. The above analyses were performed on individuals with age less than 30 in cohort 1 to avoid confounding factors due to age (*52*).

#### Public clone analysis

Please note that the definition of public clones varies across analyses. In the HLA-conditional T1D-association testing using tcrdist3, public clones are defined as TCRs sharing the same V-gene family and having exact or near-exact (single–amino acid variation) CDR3β sequences to reduce the search space and merge the highly similar sequences (Fig. 3C). In contrast, in the statistical classification framework, public clones are defined on the basis of an exact match of the V gene, CDR3β sequence, and J-gene combination (Fig. 4, C and D). To evaluate the similarity between TCR repertoires, we defined public clones as identical CDR3β sequences shared among two repertoires and defined it as below (Fig. 2, D and E)

$$\text{Public clones}\left(\%\right)=\frac{|X\cap Y|*100}{\text{min}\left(|X|,|Y|\right)}$$ (2)

where ∣X∣ and ∣Y∣ are the repertoire sizes (number of unique clones) of the repertoire *X* and *Y*. The number of shared clones between both repertoires is ∣X∩Y∣.

#### MH similarity index analysis

We used the MH similarity index (*167*) to assess the degree of similarity among TCR repertoires, taking into account the clonal frequency attributed to each unique clonotype. The MH index between a pair of repertoires is defined as

$$\text{MH}=\frac{2\sum_{i=1}^{S}x_{i}y_{i}}{\sum_{i=1}^{S}x_{i}^{2}+\sum_{i=1}^{S}y_{i}^{2}}$$ (3)

where *S* is the number of unique clones and *x* and *y* denote the frequency of *i*th clone in either repertoire. The MH index ranges between 0 (no overlap) and 1 (complete clonal overlap and identical clonal frequencies).

### Overrepresentation analysis of different disease-associated CDR3β sequences

We downloaded the McPAS (*63*) and VDJdb (*64*) datasets from the respective websites (April 2024). The unavailable, redundant, and nonhuman sequences were removed from the dataset. Only pathology (in McPAS)/antigen species (in VDJdb) with more than 30 sequences were selected for analysis. The final statistics of the datasets is given in table S1. We obtained the overlapping CDR3β sequences between cohort 1 and McPAS/VDJdb datasets using CompAIRR (*168*). We considered the clonal frequency of the overlapping sequences in each repertoire in cohort 1 and normalized the count using the following formula and grouped them by the clinical groups

$$\text{CDR}3\beta \text{frequency}={\text{Log}}_{e}\left({\text{CDR}}_{f}/R_{T}\right)$$ (4)

where ${\mathrm{CDR}}_{f}$ is the clonal frequency of the overlapping CDR3β clone in a repertoire in cohort 1 and $R_{T}$ is the sum of clonal frequencies in the same repertoire.

### HLA-conditional T1D-association testing in single–amino acid variation neighborhood

We first considered each unique TCRβ sequence within T1D-positive repertoires from cohort 2. The sequences were partitioned by the ImMunoGeneTics information system (IMGT) V-gene family, and all receptor sequences from 669 repertoires were concatenated to map each sequence to its exact and near-exact (single–amino acid variation distance) neighbors. To avoid *O*(*n*^2^) comparisons on such a large set of sequences, we identified nearly identical sequences using a fuzzy clustering technique. This method bins highly similar sequences into shared memory locations using keys with a single masked position, inspired by prior work on TCR clustering (*150*, *168*–*171*). Briefly, each CDR3 was represented as the set of possible sequences with a single masked wildcard position for either a substitution or an insertion in a Python dictionary. Each unique key was then assigned an integer index and two arrays link CDR sequence indices to masked CDR3 key indices. Group-by-key and permutation operations were then applied for rapid identification of all links between identical and near-identical CDR3 sequences. After identifying single–amino acid variation sequence neighbors of each unique CDR3 centroid, only CDR3s with at least five total neighbors in cohort 2 were retained for further analysis. Next, we considered the donors associated with each CDR3 sequence index and computed the OR of detecting a CDR3 or its near-exact single–amino acid variation neighbor across donors with class I or class II HLA alleles, computing a *P* value by a Fisher’s exact test.

A TCR feature centroid was assigned to the most statistically significant HLA allele if it had an OR of >1 with a *P* value of <1 × 10^−8^ and was detected in >5% of HLA-matched individuals and not in more than 10% of HLA-mismatched individuals. From cohort 2, this procedure yielded 20,037 unique V-gene family–CDR3β centroids assigned to either a MHC class I allele (*n* = 7255) or MHC class II allele(s) (*n* = 12,782). Next, for each strongly HLA-associated feature, we tabulated detection in cohort 1 repertoires of participants with the hypothesized restricting HLA allele. (We considered a detection if the query TCR had an exact or near exact match to a TCR with a with-in repertoires frequency greater than 1 in 500,000 templates.) Based on detections in HLA-matched repertoires, we computed the OR of observing the centroid or its neighbor in participants on the basis of T1D clinical status and computed a *P* value on the basis of Fisher’s exact test (with the Python package fishersapi based on fast-fisher) and computed and adjusted *P* value on the basis of the Benjamini-Hochberg procedure (*172*) to control false discovery rate using the Python package statsmodels. To visualize potentially T1D underrepresented and overrepresented sequence motifs derived from sequences with a false discovery rate of <0.2, we clustered these sequences into graphs using the Python package tcrdist3 (*173*) and networkx (*174*) at an edge threshold of 24 TCRdist units and plotted motifs on the basis of aligned sequences within each graph connected component.

### Quantification of HLA-based restriction of CDR3β sequence diversity

We studied the association of the HLA and CDR3β sequences using published methods (*7*) and observed the impact of T1D-risk HLA alleles on selective restriction of CDR3β sequences. The analysis was primarily performed on cohort 1 and observations were validated on cohorts 2 and 3. To assess the robustness of the findings, we also performed the analogous analysis on T1D, FDR, and CTRL repertoires, separately. SDRs (59 repertoires) were not included in this analysis because of insufficient sample number. We considered *A*, *B*, *C*, *DPA1*, *DPB1*, *DQA1*, *DQB1*, and *DRB1* HLA haplotypes in the study, and variations in the HLA alleles were obtained from genotyping experiments. The rare or common alleles for each HLA haplotype were removed from the analysis on the basis of their frequency in cohort 1 (frequency of ≥99 or <1%). Furthermore, four-digit classical alleles were partitioned on the basis of amino acid polymorphism at the HLA site (fig. S7A).

#### Position-specific CDR3β amino acid frequency analysis

First, CDR3β sequences were segregated on the basis of the length. We considered CDR3β lengths ranging from 12 to 18 (described as L12 to L18 in the manuscript) as most of the CDR3β sequences (94.17% of total sequences) were within this range (fig. S7B). All analyses focused on the international IMGT (*175*) CDR3 positions 107 to 116 that directly contact antigens (fig. S7, B and F) (*150*). The flanking positions in CDR3β sequences defined by germline-encoded V or J genes were identified using IMGT/HighV-QUEST for each length group and removed from the CDR3β sequences. The frequency of each amino acid was computed at every position within the CDR3β sequence for lengths L12 to L18. This computation involved dividing the total count of a particular amino acid by the count of all amino acids at a specific position of CDR3β sequence (fig. S7B).

#### MMLR model–MANOVA analysis

In simplest terms, the MMLR analysis is performed using *n*-dimensional independent variables to predict the *n*-dimensional dependent variables. In our study, the frequency of all amino acids at a specific position of the CDR3β sequence was used to calculate the association with counts of all amino acid polymorphism at an HLA site (fig. S7C) (*176*). We also included the top three principal components of all HLA genotypes in this analysis. Therefore, the full MMLR model was the following equation

$$y_{i}=\sum_{a=1..m-1}\beta_{a}g_{a,i}+\sum_{k=1,2,3}\pi_{k}{\text{PC}}_{k,i}+\theta$$ (5)

Here, $y_{i}$ is *n*-dimensional CDR3β amino acid frequency vector of *i*th repertoire, $\beta_{a}$ is an *n*-dimensional

parameter that represents the additive effect per group of classical alleles containing the same amino acid at specific HLA site (denoted by a in equation), $g_{a,i}$ is the allele count of allele group a in *i*th repertoire. We included *m* − 1 group of classical alleles, casting 1 group as the reference. $\pi_{k}$ is an *n*-dimensional parameter that represents the effect of the *k*th principal component, and ${\text{PC}}_{k,i}$ is the value of the *k*th principal component of *i*th repertoire. θ is an *n*-dimensional parameter that represents the intercept.

The null model only had the terms for covariates without allelic effects

$$y_{i}=\sum_{k=1,2,3}\pi_{k}{\text{PC}}_{k,i}+\theta$$ (6)

The improvement in the model fit between the full and null model was estimated using MANOVA and the significance of the improvement was assessed using Pillai’s trace. Codes from a previous study (https://github.com/immunogenomics/cdr3-QTL) were modified and reused accordingly in the study using Python and R languages (*7*). Significant associations were determined using a false discovery rate threshold of <0.05.

#### HLA risk score

The mutations in *HLA-DQB1* at site 57 and *HLA-DRB1* at site 13 and 71 have previously been associated with T1D disease progression based on 18,832 case-control samples of T1D (*79*). In this analysis, we grouped the classical four-digit *HLA-DQB1* and *HLA-DRB1* alleles on the basis of the amino acids present in these three sites, along with the T1D risk in terms of OR (table S5). The HLA risk score was calculated on the basis of the sum of the individual OR scores multiplied by the number of alleles containing a specific amino acid polymorphism (fig. S7D). In case multiple combinations were possible for the three amino acids, then we consider the combination with the highest OR score.

#### CDR3 risk score

The association of CDR3β amino acids with HLA risk score is referred to as CDR3 risk score. We developed LR models between the HLA risk score and CDR3β frequency of each amino acid at specific CDR3β positions (for all lengths running from L12 to L18) as described previously (*7*, *176*). The *P* values obtained from the LR model were further adjusted for multiple testing using the Benjamini-Hochberg method. The correlation coefficients of the significant association between the amino acid frequency and HLA risk score were denoted as the effect size of that particular T1D-associated CDR3β amino acid or CDR3 phenotype (fig. S7E). The number of significant associations was dependent on the number of repertoires in the study. Therefore, we observed a higher number of associations for cohort 1.

The cumulative effect sizes of CDR3 phenotypes were calculated for the whole CDR3β sequence, and CDR3 risk score for the entire repertoire was derived by averaging the cumulative effect sizes of all CDR3β sequences (fig. S7E). Further, we calculated the correlation between HLA risk score and CDR3 risk score for each length and also compared CDR3 risk scores across clinical groups.

#### High-risk HLA-associated motif identification from CDR3 phenotypes

The CDR3 phenotypes, derived from the HLA risk score, were transformed into sequence motifs to evaluate their association with clinical groups. The amino acids were grouped on the basis of the IMGT positions, and a cutoff of effect sizes ≥0.1 was used to select significant associations. Positive and negative effect sizes were analyzed separately to identify positively associated (pHLA motif) and negatively associated (nHLA motif) motifs with HLA-based T1D risk. The analysis was focused on CDR lengths L13 to L16, which had a higher occurrence in the repertoires (see table S8). Furthermore, IMGT position aligned amino acids showed the repetitive behavior of amino acids on the C-terminal end, leading to selection of the conserved regions (IMGT positions 107 to 111). The effect sizes of consensus motifs in the logo plot represent the average effect sizes of CDR3 phenotypes for lengths L13 to L16.

#### Comparative analysis of CDR3 risk score across T1D clinical groups

We calculated CDR3 phenotypes and their effect sizes across repertoires from different clinical groups, including T1D, FDR, and CTRL. Notably, an overlap was found in CDR3 phenotypes across the clinical groups. To appropriately test for association between CDR3 risk score and clinical groups, the CDR3 phenotypes and their corresponding effect sizes, derived from a specific clinical group, were applied to calculate the CDR3 risk score across all clinical groups. It is crucial to emphasize that the CDR3 phenotypes for each clinical group were computed independently of other clinical groups. As a control, we shuffled the effect sizes of the CDR3 phenotypes obtained from cohort 1 to highlight the importance of effect sizes and position-specific nature of the CDR3 phenotypes.

#### Analysis of heterozygous HLA allele-based restriction of TCR diversity

We used IGoR, a probabilistic model of V(D)J recombination, to model the recombination processes of unproductive TCR sequences. The sequences were aligned, and the recombination parameters were inferred through iterative learning. Multiple rounds of inference were performed to ensure robust parameter estimation. The inferred models were saved, and gene usage patterns were visualized for each sample. Note that raw sequences without any filtering criteria were used in the IGoR model (*91*). Further, the productive TCR sequences were analyzed using soNNia (*92*), a selection model that builds on the recombination probabilities learned by IGoR. soNNia inferred selection pressures by modeling the observed TCR sequences and generating a large number of synthetic sequences for comparison. The model was trained over 80 epochs to optimize selection parameters, and its performance was evaluated by comparing the generative (*P*gen) and posterior (*P*post) sequence probabilities. Model performance was assessed by calculating the entropy of the sequence distributions both pre- and postselection, which provided insights into the diversity and selection pressures within the TCR repertoire.

The HLA genes *A*, *B*, *C*, *DPA1*, *DPB1*, *DQA1*, *DQB1*, and *DRB1* were considered to assess the heterozygosity of HLA alleles. We calculated the total count of homozygous alleles in HLA genes (ranging from 0 being heterozygous and 8 being homozygous) and assumed the equal contribution of the HLA genes in the TCR diversity.

To assess the clustering of the V-gene frequencies, *TRBV28* was used as a case study, where it was segregated as low frequency (*f* < 0.015), midrange frequency (0.015 ≤ *f* ≤ 0.04), and high frequency (*f* > 0.04). For each subset, bootstrapped samples were generated by resampling 30% of the data multiple times, allowing for robust estimation of frequency distributions with respect to HLA alleles. The mean and SD of allele frequencies were computed across the resampled datasets to capture the variability in the data.

### ML approaches for repertoire classification and motif identification

#### ML analysis data preprocessing and general ML workflow

We used a combination of ML and DL approaches to classify T1D status and identify T1D-associated motifs within immune repertoires. Cohort 1 served as the training set for all methods, while repertoires from cohorts 2 and 3 were used as a held-out test set. The T1D class was considered a positive class, and remaining clinical statuses—FDR, SDR, and CTRL—were considered non-T1D or negative class.

In the *k*-mer–based LogReg and DeepRC model, the same fivefold CV dataset was used to train both model parameters and hyperparameters in cohort 1. The data were split into five random subsets, with T1D and control samples drawn separately without replacement for each fold. Each fold contained an approximately equal number of samples and maintained a consistent ratio of control to T1D samples. Moreover, each repertoire was assigned a weight on the basis of the prevalence of their age group and the distribution of disease states across these groups, thereby normalizing the influence of age on the classification model (see Supplementary Note for more detail). The first and last four amino acids in each CDR3β sequence were also excluded to avoid the effect of V and J genes in ML/DL methods.

#### Statistical classification framework for T1D repertoires using public clones

We used the ImmuneML platform (*96*) to implement the statistical classification framework (*20*) on the T1D dataset, which uses unique V-gene, CDR3, and J-gene information. The YAML file was prepared following the immuneML documentation (https://docs.immuneml.uio.no/latest/usecases/emerson_reproduction.html). In summary, we used the “CompAIRRSequenceAbundance” encoding on the basis of the CompAIRR tool (*168*) to enable faster repertoire level comparisons. Model performance was assessed across varying significance thresholds (*P* values = [0.001, 0.0001, 0.00001, 0.000001]). The optimal model was selected on the basis of optimization of log-loss values in fivefold CV. Log loss measures the divergence between predicted probabilities and the actual class value, with lower log loss indicating better performance. We further calculated the maximum balanced accuracy and AUROC to analyze the performance of the model. The optimal model was further tested on cohorts 2 and 3 to assess its predictive performance in unseen datasets.

#### k-mer–based classification model using LogReg

The linear subsequence information at the 4-mer level was used as a feature set to classify T1D status. A 4-mer frequency matrix of 160,000 (20^4^; number of possible 4-mers) by 1298 (number of repertoires in cohort 1) was generated. To populate this matrix, we used a sliding window approach, where a window of four consecutive amino acids moved across each CDR3β sequence. The frequency of occurrence of each unique 4-mer was recorded in the matrix for its respective repertoire. This 4-mer frequency matrix was then used as a feature set in a LogReg to classify the T1D statuses of immune repertoires. For model training, we applied a fivefold CV on cohort 1, where three folds were used for training, one fold for tuning, and one fold for testing, with the process repeated recursively. The outputs of the five models, one from each fold, were then assembled into a single model by averaging their predictions after applying the sigmoidal activation function. This ensemble model was subsequently tested on a held-out dataset consisting of cohorts 2 and 3. The following hyperparameter ranges were used for the training of LogReg model:

Penalty: L1, L2, Elastic-Net penalty, or no penalty

C: random value in [0.1, 10)

Solver algorithms: Newton-CG, LBFGS, Liblinear, SAG, SAGA

Number of iterations: {100, 500, 1000, 5000}

#### DL-based multiple-instance learning model for classification of T1D status

*Development of DeepRC model*We used DeepRC (*36*), a DL method based on continuous modern Hopfield networks (MHNs) (*177*), within a multiple-instance learning framework to classify immune repertoires based on T1D status. In this method, each TCR repertoire was treated as a sample, classified as either T1D or CTRL, while individual CDR3β sequences within the repertoire were viewed as instances. Thus, each sample is represented as a “bag” of CDR3β sequences with a single class label.

The DeepRC architecture used a convolutional neural network (CNN) as an encoder for processing individual sequences. Input vectors were structured with dimensions corresponding to sequence_length × *n*_amino_acids, where the sequence length may vary across sequences. A learned kernel of shape (kernel_size × *n*_amino_acids) was convolved along the amino acid positions of each CDR3β sequence, and feature-wise max pooling was applied to obtain a fixed-sized feature vector of shape (*n*_sequences × kernel_size), where *n*_sequences may differ between repertoires. A learned MHN attention pooling mechanism, as described in (*36*), was used to pool the instances in a repertoire of shape (*n*_sequences × kernel_size) into a single feature vector of shape (kernel_size). This results in a single feature vector of shape (kernel_size) as representation for a single repertoire. Last, the feature vector was passed to a fully connected output layer, consisting of one output unit, to predict the class label of the repertoire.

All trainable parameters in the DeepRC architecture were optimized end-to-end using the Adam optimizer in PyTorch, with a binary cross-entropy loss function. Similar to LogReg, we assigned three folds for training, one fold as tuning, and the remaining one fold as test set, recursively, in a fivefold CV setup. We train the DeepRC model on the training set while using the tuning set loss as early stopping criteria. The best-performing model for each CV fold was selected on the basis of the AUROC of the tuning set, resulting in five distinct “best” models from the CV process. Subsequently, a LogReg model was trained to aggregate the predictions from these five best models, forming the final DeepRC model, which was then evaluated on the held-out test set. The following parameters were used for the training of DeepRC model:

Learning rate: 5 × 10^−5^

Number of weight updates: 3 × 10^5^

Number of CNN kernels: {8, 16, 32, 64}

Number of CNN layers: 1

CNN kernel sizes: {5, 7, 9}

Number of attention layers: 2

Number of neurons per attention layer: {32}

Number of output network layers: 1

Number of units per output network layer: 64

Weight decay penalties ({l1 term, l2 term}): {{0, 0}, {1 × 10^−5^, 1 × 10^−3^}, {1 × 10^−4^, 1 × 10^−2^}}

*T1D-associated motif identification from DeepRC.* DeepRC supports different ways of interpretability, via the attention values and via the contribution analysis method IGs (*102*). We applied the IG method to the trained DeepRC model from each CV fold as described in the original paper (*36*). It allows visualization of the contribution of inputs and weights to the prediction of the DeepRC models, which could then be used to manually extract motifs. For each DeepRC model in the CV, we compute the IGs such that we obtain the contribution of the input sequences to the DeepRC model output. In this step, we compute IG on the T1D-positive samples of the tuning set samples that were also used for early stopping. The training split samples were not used here as they have already been overfitted by the DeepRC model. Subsequently, for each DeepRC model, we identified the input sequence with the highest contribution to the T1D prediction per repertoire. The DeepRC model with null coefficient in the ensemble LogReg model was excluded from the motif calculation. The identified sequences from each DeepRC model, corresponding to T1D-positive repertoires in the tuning set, were collected into a FASTA file and subsequently analyzed with GLAM2 (*178*) to compute motifs through sequence alignment

glam2p−p−b5−D0.01−I0.0001−s1

We chose a motif width of -b 5, as it corresponds to the smallest motif width matching the CNN kernel size. The first and last four amino acids of every input sequence were cropped before alignment. The final selected motif [FWY],([AFHILMPQRSTVY]){0,1},[EFGHSWY],([AFQT]){0,1},[ACDEGHIKNPQRSTVY] contains two deletions at positions 2 and 4 in the motif (fig. S21).

Consensus sequences containing both the pHLA motif and DeepRC motif were obtained by sequentially filtering the T1D dataset: first by the presence of DeepRC motif, followed by further filtering based on presence of pHLA motif. The resulting intersection of the pHLA motif and DeepRC motif is termed the consensus motif. Similar to other motifs, the consensus-motif score was calculated by normalizing the number of motif-containing sequences with the total CDR3β sequence count in the respective repertoire.

### QTL analysis

Quality control measures were performed to remove data from individuals that were sex discordant, related, and/or showed unusual levels of heterozygosity, as previously described (*127*). Genetic ancestry was inferred using Admixture software (*179*) for projection analysis on the 1000 Genomes cohort (*180*). QTL analysis was performed to detect associations between TCR motif frequency and 240 T1D risk variants (*127*) that were directly genotyped or imputed, as previously described. For PBMC data reported in this publication, LR was performed assuming an additive genotypic effect with age, sex, T1D status, predicted probability of CMV infection (*127*), and 10 multidimensional scaling components as covariates. For nPOD tissue data, age, sex, diabetes status, and 10 multidimensional scaling components were included, and, for sorted peripheral blood data, age, sex, and T1D status were included as covariates.

## Supplementary Materials

### The PDF file includes:

Supplementary Note Legends for tables S1 to S11 Figs. S1 to S25 References
