# Supplementary material for: Identification of a type 1 diabetes–associated T cell receptor repertoire signature from the human peripheral blood
Source: Sci Adv. 2026 Feb 13;12(7):eadx7448. doi: 10.1126/sciadv.adx7448 (PMC12904210; doi:10.1126/sciadv.adx7448)
Supplement: Supplementary file 1 — Supplementary Note Legends for tables S1 to S11 Figs. S1 to S25 References [file sciadv.adx7448_sm.pdf]

Science Advances
AAAS

Supplementary Materials for

Identification of a type 1 diabetes–associated T cell receptor repertoire signature from the human peripheral blood

Puneet Rawat *et al.*

Corresponding author: Puneet Rawat, puneet.rawat@medisin.uio.no;
Victor Greiff, victor.greiff@medisin.uio.no; Todd M. Brusko, tbrusko@ufl.edu

Sci. Adv. **12**, eadx7448 (2026)
DOI: 10.1126/sciadv.adx7448

The PDF file includes:

Supplementary Note
Legends for tables S1 to S11
Figs. S1 to S25
References

Other Supplementary Material for this manuscript includes the following:

Tables S1 to S11

Supplementary Note

Terminology used in the manuscript

TCR features refer either to V gene-restricted CDR3β sequence clusters, as described in “Limited T1D association of HLA-associated public TCRs”, or to repertoire-level risk scores and motif-based metrics, as outlined in “Amino acid (AA) variation in HLA risk alleles restricts the position-wise AA frequency of the TCR repertoire” (see Results). The term "T1D-associated T cell repertoire signatures" (or T1D-associated signatures) encompasses all motif-based metrics used throughout the manuscript, including DeepRC-motifs, pHLA-motifs, nHLA-motifs, and consensus-motifs. CDR3 phenotypes are defined as position-specific amino acid residues within the CDR3β region that exhibit statistically significant associations with the HLA risk score; the strength of these associations is quantified by correlation coefficients, which are referred to as effect sizes. The definition of public clones varies across analyses: in the section “Limited T1D association of HLA-associated public TCRs”, public clones are defined as TCRs sharing the same V-gene family and having exact or near-exact (single AA variation) CDR3β AA sequences; in "The presence of public clones is insufficient for the classification of T1D status", they are defined by an exact match of the V gene, CDR3β AA sequence, and J gene combination; and in "Repertoire-level similarity and diversity do not differ between T1D clinical groups", public clones are defined as identical CDR3β AA sequences shared among two repertoires (see Results). Additionally, the terms "individual" and "participant" are used interchangeably throughout the manuscript.

Assessment of sequencing depth

A separate set of replicate experiments were conducted on cohort 1 to gauge the sequencing depth and precision of sequencing technology. The replicate experiment consisted of 7 shallow sequenced replicates, 38 deep sequenced replicates, and one shallow and deep sequenced replicate. The clones are primarily defined as CDR3β sequences, unless explicitly stated otherwise. The clonal frequencies were merged for identical CDR3β AA sequences. The dataset was divided into 2 sections, where repertoires used in shallow vs shallow sequencing comparison were considered **Dataset A** and repertoires used in shallow vs deep sequencing comparison were considered **Dataset B**.

First, to assess the reproducibility of our experiments, we employed Pearson correlation (r) and Morisita-Horn (MH) similarity index, which incorporate both clonal overlap and clonal frequencies in their assessments, on shallow and deep sequenced technical replicates in cohort 1 (see **Methods**). Our results demonstrated high Pearson correlation and MH index values of shallow replicates with (i) shallow replicates ($r = 0.99 \pm 0.01$ and MH index $= 0.92 \pm 0.12$ for 7 replicates, **Dataset A**) and (ii) deep replicates ($r = 0.98 \pm 0.03$ and MH index $= 0.87 \pm 0.16$ for 38 replicates, **Dataset B**) (Fig. S2). Additionally, we sequenced two shallow and two deep replicates for one individual and observed high clonal overlap among the various combinations of shallow-shallow, shallow-deep, and deep-deep sequenced replicates ($r > 0.98$ and MH index > 0.82 for all six possible combinations). Taken together, we

demonstrate high technical reproducibility and adequate sequencing depth to capture the clonal diversity of TCR repertoires.

Robustness assessment of HLA-based CDR3 restriction based on different clinical groups

We first conducted MMLR-MANOVA tests on the T1D, FDR, and CTRL repertoire sets individually to quantify the association between each HLA site and CDR3β position. These analyses revealed predominantly class II HLA (DQA1, DQB1, and DRB1 genes)-based restrictions on CDR3β amino acid positions in each clinical group, consistent with the observations from cohort 1 (Fig. S8, Fig. S9). Therefore, we further conducted a robustness analysis of CDR3 phenotype and CDR3 risk score based on two main objectives, which includes whether CDR3 phenotypes obtained from specific clinical group (i) show overlap with other clinical statuses and (ii) demonstrate the similar association pattern (T1D>FDR>SDR>CTRL), as observed for cohort 1.

We observed 183, 251 and 68 significant CDR3 phenotypes for T1D, FDR and CTRL clinical groups, respectively using LR test between positional CDR3β frequencies of each AA and HLA risk score. CDR3 phenotypes calculated for each clinical group had significant overlap with each other, for example, the overlap of CDR3 phenotypes was around 63% (43 AAs) for T1D (183 AAs) with respect to CTRL (68 AAs) and 52% (95 AAs) for FDR (251 AAs) with respect to T1D (183 AAs). The number of significant CDR3 phenotypes was proportional to the number of repertoires in each set. Therefore, it is expected to have fewer CDR3 phenotypes for the smaller repertoire set (e.g., only 68 phenotypes were observed for the CTRL group with 182 repertoires).

We further calculated CDR3 risk score for each repertoire using CDR3 phenotypes obtained from each clinical group. We observed that CDR3 risk score shows the same association with each clinical groups as observed in cohort 1, where average CDR3 risk score was highest for the T1D repertoires followed by FDR, SDR and CTRL repertoires (T1D>FDR>SDR>CTRL), respectively, across different lengths, irrespective of which clinical group was used to calculate the CDR3 phenotypes (Fig. S11). Moreover, similar to observation from cohort 1, The CDR3 risk score showed positive correlation with HLA risk score for repertoires from T1D (~0.4%), FDR (~0.46%) and CTRL (~0.54%) clinical groups (Fig. S8, Fig. S9).

Assessment of factors influencing CDR3 risk score in T1D

We hypothesized that observed difference in CDR3 risk score among different clinical groups can arise from two possibilities: (i) the count of CDR3β sequences containing CDR3 phenotype was different among repertoires of different clinical groups or (ii) the effect sizes of the CDR3 phenotype affect the overall CDR3 risk score. To address the first possibility, we examined the CDR3β sequences in each repertoire containing at least one CDR3 phenotype (calculated on cohort 1) and observed that there were no significant differences in the percentage of CDR3β sequences across clinical groups (ranging from average of ~90.4% for length 12 to ~99.7% for length 18; Table S7).

The availability of large cohorts resulted in more significant CDR3 phenotypes (p-value<0.05), which in turn led to a higher percentage of CDR3β sequences containing these CDR3 phenotypes in cohort 1. For the second possibility, we shuffled the effect size of the T1D-associated CDR3β AAs for T1D clinical group and recalculated the CDR3 risk score (Fig. S11D). The expected trend of CDR3 risk score (T1D>FDR>SDR>CTRL) was lost after shuffling the effect sizes, highlighting its importance for the CDR3 phenotypes. This demonstrates that although almost all CDR3β sequences contained at least one CDR3 phenotype, the risk observed for T1D in TCR cohorts was mainly observed from the effect sizes of these T1D-associated CDR3β AA, in other words, how strongly these CDR3β AAs associated with HLA risk score.

Role of positive and negative effect sizes of CDR3 phenotypes

The T1D-associated CDR3 phenotypes exhibited both positive and negative effect sizes, indicating that these T1D-associated position-specific CDR3β AAs correlate both positively and negatively with the HLA risk score. Therefore, we further assessed the importance of the positively and negatively correlated CDR3 phenotypes in cohort 1. As expected, CDR3 risk score based on positively (positive effect sizes)- and negatively-associated CDR3 phenotype (negative effect sizes) exhibited a positive correlation with HLA risk score (Fig. S8). The correlation of HLA and CDR3 risk scores obtained from either positive or negative effect sizes of CDR3 phenotypes was marginally different from the correlation obtained from the cumulative effect sizes. CDR3 risk scores based on both positive and negative effect sizes were also grouped based on high-risk HLA allele types (DR3 and DR4) for each clinical group, which showed an association with high-risk HLA allele types. The CDR3 risk score for DRX/X (non-risk allele type) HLA individuals was almost consistently lower than for the individuals containing at least one T1D-associated risk allele DR3 or DR4 (Fig. S10). It is important to note that we observed a positive correlation with HLA risk score and higher CDR3 risk score for T1D compared to CTRL repertoires (Fig. S8) for CDR3 risk score obtained from the negatively-associated CDR3 phenotypes (although in negative numbering scale). The negatively-associated CDR3 phenotypes can be utilized to identify CDR3β motifs that were less frequent in T1D repertoires, as observed in subsequent analyses.

Analysis of CDR3β motifs obtained from the HLA-based restriction analysis

In the consensus positively-associated HLA-motif (pHLA-motif; [LVSICY][FLWHY][FMWNSIEHY][FWPDVIEHY][FWDEHY]), aromatic and hydrophobic residues were present in almost all positions; whereas, negatively charged residues were predominantly present at the C-terminus of the motif. The C-terminal residues were also more redundant across different lengths, even when additional positions were included for longer CDR3β lengths (Fig. S13). Similarly, a consensus negatively-associated HLA-motif (nHLA-motif; [KR][AGHIKMTV][ADKRT][GKQRT][GIKLTV]) was also derived from the negatively-associated CDR3 phenotype (Fig. S14). Negatively associated CDR3 phenotypes had relatively fewer observable recurring patterns across different CDR3β lengths (see **Methods**). However, positively charged residues

were predominant in almost all positions in the motif, showing conserved biophysical compatibility among HLA alleles and restricted TCR repertoire (*183*).

It is important to note that pHLA- and nHLA-motif scores have varying frequency within a repertoire and should not be compared with each other. In summary, we successfully identified HLA-associated CDR3β motifs exhibiting gradual overrepresentation or diminution across different clinical groups in cohort 1.

Performance of the conventionally considered high-risk HLA alleles in classification of immune repertoires

The HLA DR3 and DR4 alleles were strongly associated with a high-risk of T1D. Consequently, we utilized an individual's HLA type to evaluate the predictability of their clinical statuses with regards to T1D. By considering the presence of at least one DR3 or DR4 allele as a predictor of T1D, we achieved an accuracy of 57.1%, (sensitivity of 75.6% and specificity of 48.2%) in cohort 1 and 80.1% (sensitivity of 82.3% and specificity of 71.9%) in cohort 2 and 3 (Fig. S16A). We also employed stricter criteria, requiring both alleles to be either DR3 or DR4 as a predictor of T1D. This led to an accuracy improvement to 72.4%. However, we observed a significant decrease in sensitivity (32.1%) while specificity increased substantially to 91.8%. Whereas cohort 2 and 3 showed an accuracy of 47.5% (sensitivity of 33.8% and specificity of 97.2%). Approximately 85% of the repertoires in cohort 1 carried at least one HLA DRX allele, and the majority of these repertoires were present in non-T1D clinical groups. This explains the higher specificity observed with the stricter criteria. Indeed, our results aligned closely with the documented knowledge that the presence of two T1D risk-associated HLA alleles (i.e., DR3/3, DR4/4, DR3/4) associates with a higher incidence of T1D compared to one or no copies of these risk alleles (e.g., DR3/X, DR4/X, DRX/X) (*184*, *185*). However, such heterozygous individuals carrying HLA DR3/DR4 were relatively few in number (n=211 with 61.1% of those belonging to the T1D group), and among the 1149 individuals with one or no allele containing DR3/DR4, 276 (24%) had T1D leading to lower sensitivity (Fig. S16A).

Age-based confounding factors reduction using sample weights

We derived age-dependent sample weights to balance data distribution across age groups and disease classes. We first computed age bins and assigned each sample to an appropriate age group, then calculated sample weights for each sample based on the distribution of age groups and disease states. These sample weights were designed to correct for over-representation by down-weighting samples from highly represented groups and up-weighting those from underrepresented groups, effectively normalizing the contribution of each sample to the overall loss function. The pseudo-code for the process is given below:

```
total_n_samples = 1298 # total number of samples in cohort 1
age_groups = bin_ages(ages, n_bins=10)
contingency_table = create_contingency_table(disease_states, age_group)
for cell in contingency_table:
      cell    =    n_samples_in_age_group    *    n_samples_in_disease_state    /
(total_n_samples * cell)
```

```
# For (age_group, disease) combinations with no samples
replace_infs_with_1(contingency_table)

# sum of sample weights is equal to the number of samples
weights = get_weights_based_on_disease_and_age_group(data, contingency_table)
```

Supplementary Tables

Table S1 | Statistical Overview of the McPAS and VDJdb Databases (as of April, 2024).

Table S2 | Distribution of pathology (McPAS) and antigen species (VDJdb)-associated CDR3β sequences across clinical groups present in cohort 1. The normalized frequency of the CDR3β clones was calculated by dividing the clonal frequency of the overlapping CDR3β clones in a repertoire by the total sum of clonal frequencies of all CDR3β clones in the same repertoire. Values are presented as mean ± standard deviation and p-values were obtained from the Kruskal-Wallis test and adjusted for multiple testing using the Benjamini-Hochberg procedure. Significant values where normalized frequencies are higher for T1D compared to other clinical groups are highlighted in violet.

Table S3 | Unique V-gene family-CDR3β centroids assigned to either MHC class I or class II alleles. Values with FDR < 0.2 that are overrepresented (red) or underrepresented (blue) in the T1D clinical group are highlighted.

Table S4 | P-values obtained from the MMLR-MANOVA tests for the whole cohort 1 and each clinical group (T1D, FDR and SDR). P-values were adjusted for multiple testing using the Benjamini-Hochberg procedure.

Table S5 | The odds ratios for T1D risk based on DRB1 and DQB1 HLA alleles (sourced from https://github.com/immunogenomics/cdr3-QTL/tree/main/data/genotype).

Table S6 | Observed CDR3 phenotypes and their effect sizes for Cohort 1 and for each clinical group (T1D, FDR, CTRL). The significant CDR3 phenotypes (p-value<0.05) are highlighted in blue.

Table S7 | The average percentage of CDR3β sequences containing at least one CDR3 phenotype in each clinical group and for different CDR3β sequence lengths.

Table S8 | Calculation of positively- (pHLA) and negatively- (nHLA) associated HLA-motifs by combining the IMGT-numbered positions from CDR3β sequence lengths L13 to L16.

Table S9 | Performance of the different methods utilized in the current study on cohort 1, and held-out test cohorts 2 and 3. Note: some methods, such as HLA risk score and different motif-based approaches, may not involve direct training on cohort 1.

Table S10 | Performance matrices for each split in statistical classification method using public clones. The best-performing encoding is highlighted in violet.

Table S11 | T1D-associated V-gene/CDR3/J-gene clones obtained from statistical classification using public clones.

Metadata information

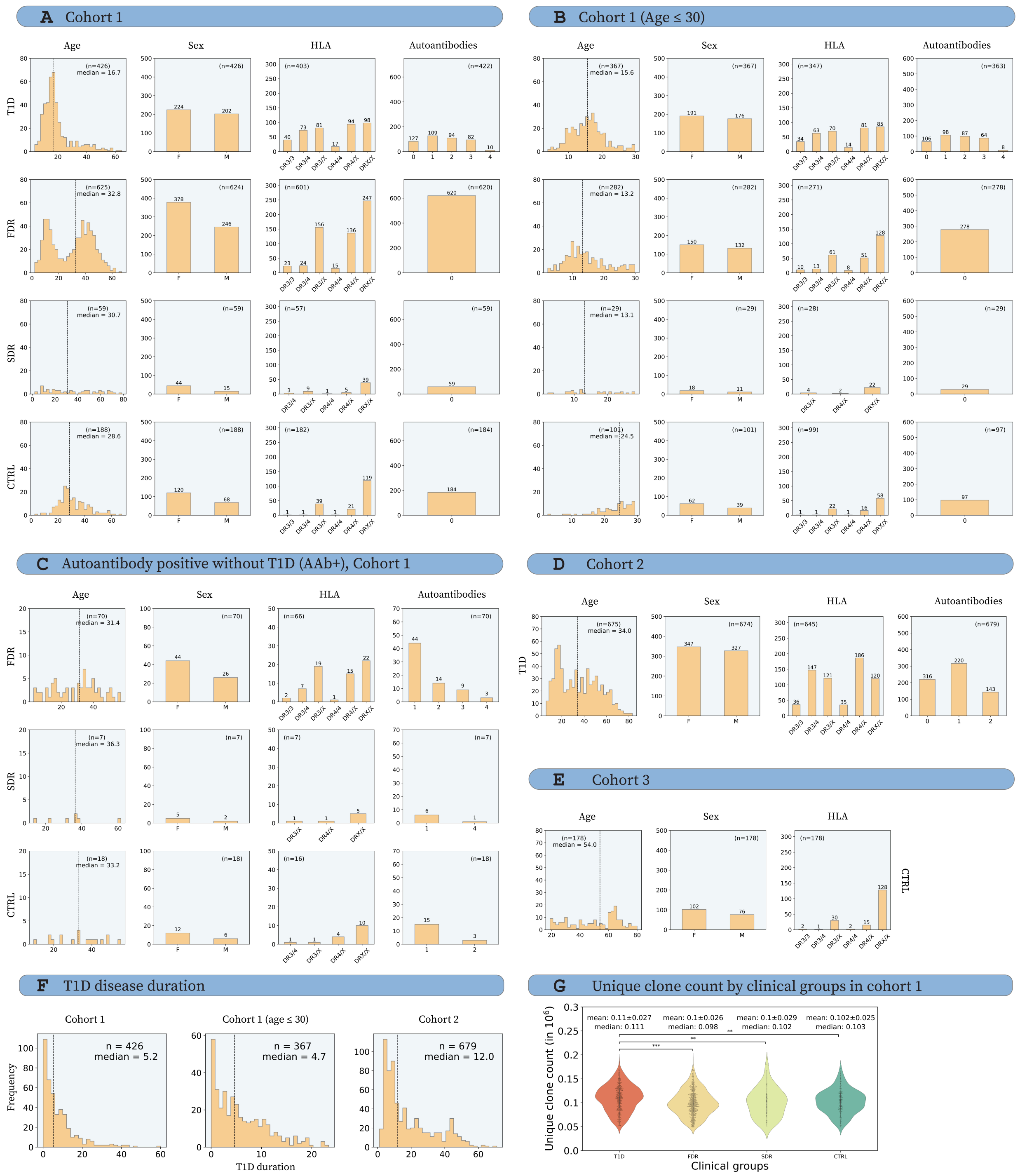

Fig. S1 | The metadata information for the TCRβ repertoires. The metadata information includes age, sex, human leukocyte antigen (HLA) and autoantibody distribution in the **(A)** cohort 1, **(B)** cohort 1 filtered based on age (age ≤ 30 years), **(C)** "AAb+" repertoires in cohort 1, which are at risk of developing T1D (i.e., contains significant concentration of one or more autoantibodies but not classified under T1D clinical group), **(D)** cohort 2 (contains only T1D repertoires) and **(E)** cohort 3 (contains only control repertoires). The autoantibody information was not available for the TCRβ repertoires in cohort 3. "AAb+" repertoires, although part of cohort 1, were kept as a separate category and excluded from all analyses unless specified. **(F)** The duration (in years) for T1D clinical group in: cohort 1, cohort 1 with age ≤ 30 years and cohort 2, respectively. "n" is the number of data points

considered in the respective metadata information plot. The median line was plotted for all age-related metadata information along with the median value. **(G)** The number of unique clones observed in cohort 1, when grouped by clinical groups, shows minimal differences in both mean and median values. p-values for pairwise testing were calculated using two tailed Mann-Whitney U tests and p-values were adjusted between clinical groups. All p-values were adjusted for multiple testing using the Benjamini–Hochberg method. p-values were described as * for [0.01,0.05], ** for [0.001,0.01] and *** for <0.001 and no stars plotted for non-significant values. Relates to **Fig. 1**.

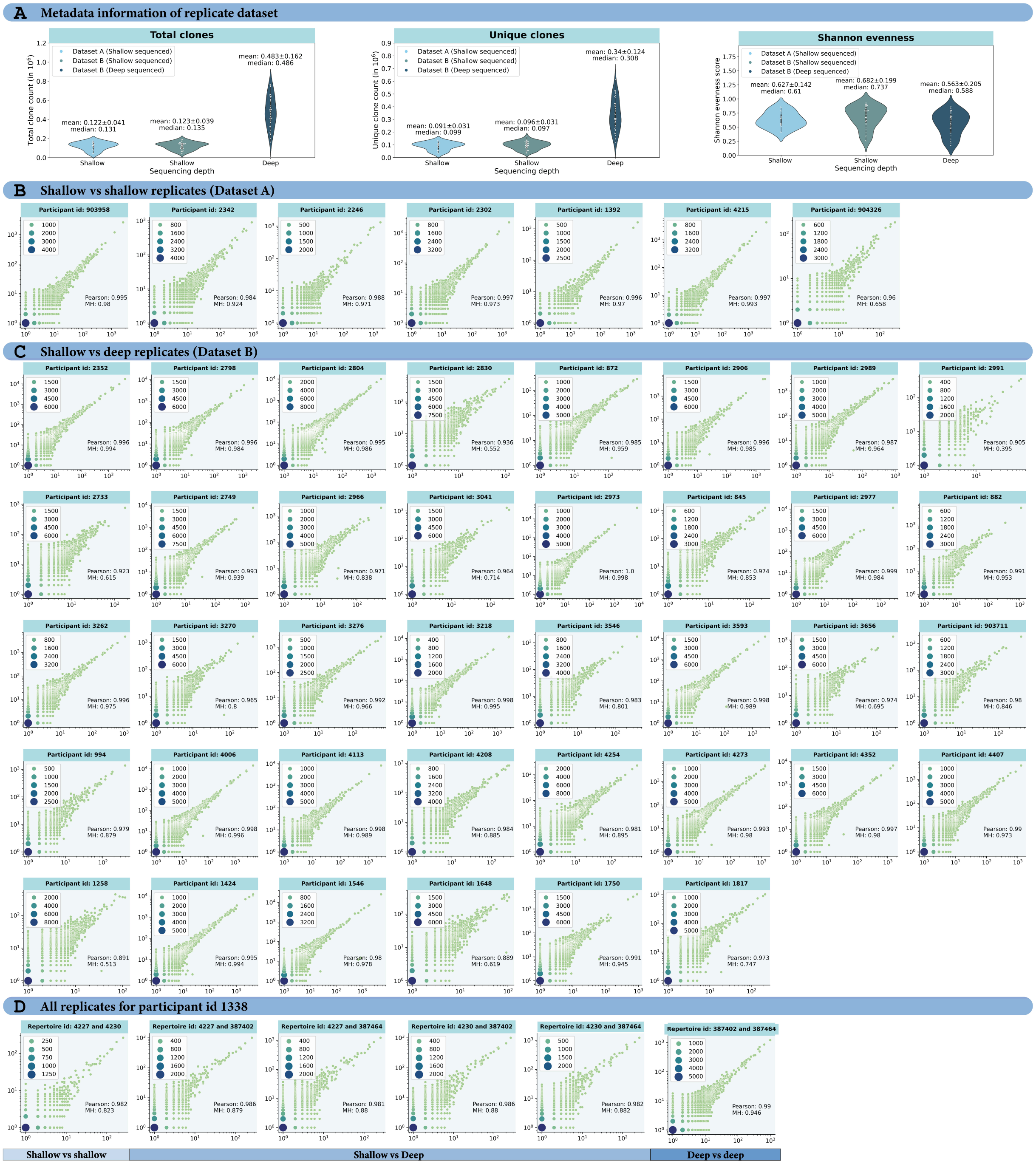

Fig. S2 | High overlap observed between the technical replicates of shallow vs shallow and shallow vs deep sequenced repertoires demonstrate high reproducibility of the experiments. Technical replicates are defined as two samples obtained from the blood of the same participant, while clones are defined as unique CDR3β sequences. **(A)** metadata information (total clones, unique clones and shannon evenness) of the technical replicate dataset, where repertoires used in shallow vs shallow sequencing comparison were considered **Dataset A** and repertoires used in shallow vs deep sequencing comparison were considered **Dataset B.** To assess the reproducibility of our experiments, we employed Pearson correlation (r) and Morisita-Horn (MH) similarity index, which incorporate both clonal overlap and clonal frequencies in their assessments (annotated on each plot). Scatterplots of sequence counts of one replicate versus the other replicate of the same participant were presented for **(B)** shallow vs shallow sequenced repertoires and **(C)** shallow vs deep sequenced repertoires. **(D)** participant id 1338 that had two shallow and two deep sequenced repertoires. Therefore, all combinations of shallow and deep sequenced repertoires were plotted. The results demonstrated high Pearson correlation and MH index values of shallow replicates with (i) shallow replicates (r = 0.99 ± 0.01 and MH index = 0.92 ± 0.12 for 7 replicates) and (ii) deep replicates (r = 0.98 ± 0.03 and MH index = 0.87 ± 0.16 for 38

replicates). Additionally, two shallow and two deep sequenced replicates for participant id 1338 observed high clonal overlap among the various combinations of shallow-shallow, shallow-deep, and deep-deep sequenced replicates ($r > 0.98$ and MH index > 82 for all six possible combinations). Taken together, we demonstrate high technical reproducibility and adequate sequencing depth to capture the clonal diversity of TCRs. Relates to **Fig. 1**.

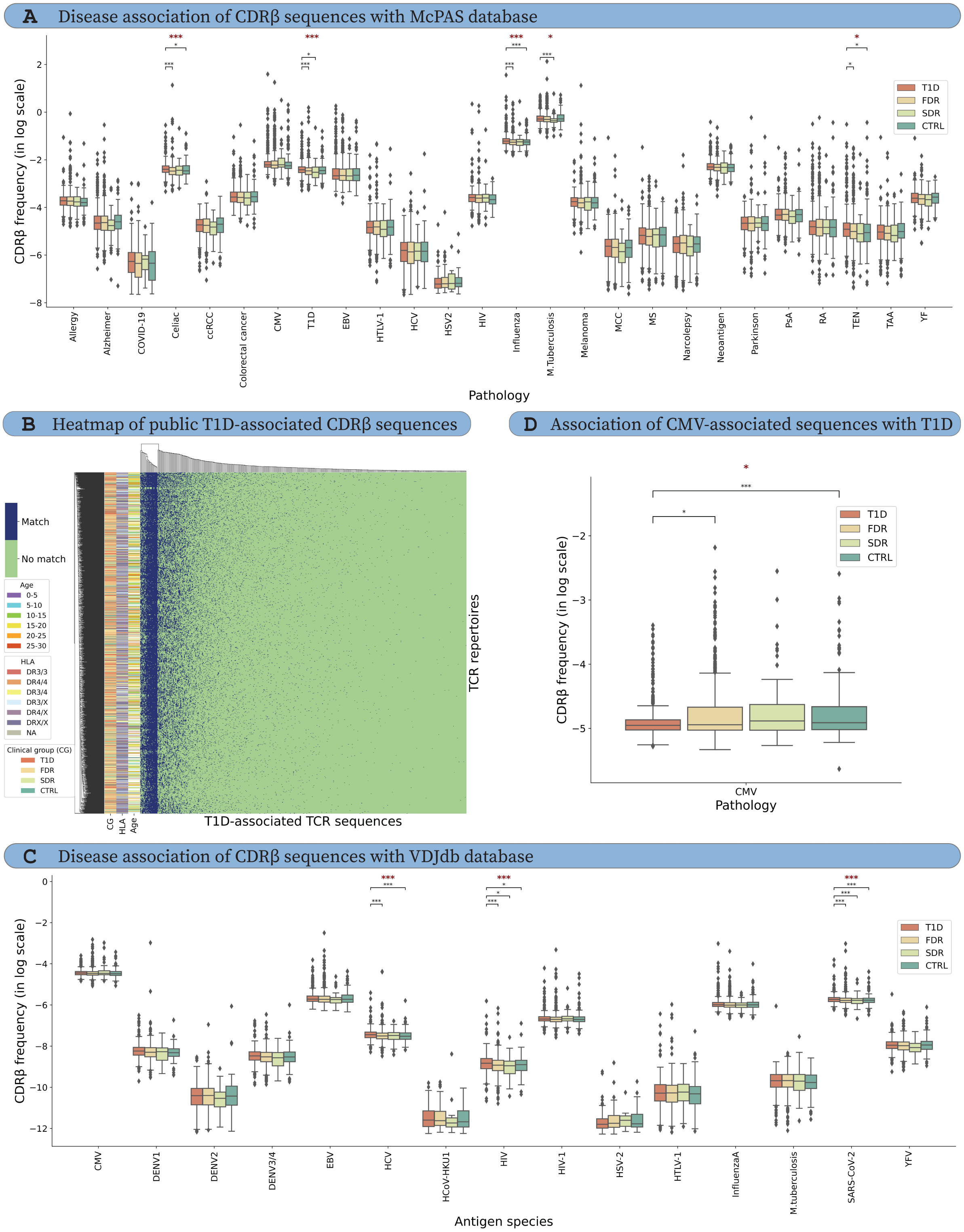

Fig. S3 | The publicly available inflammatory disease and T1D-associated CDR3β sequences demonstrate association with clinical groups in cohort 1. The unique clone count of CDR3β sequences in cohort 1, which were also present in the McPAS/VDJdb database for given pathology (as detailed in Table S1), were averaged for each repertoire and normalized by the total clone count. These normalized values were further clustered based on the clinical groups and visualized in log scale. (**A**) Sequence matches were calculated for different pathologies in McPAS database. (**B**) The distribution of T1D

pathology-associated CDR3β sequences in each repertoire. Individual T1D-associated CDR3β sequences revealed a small subset of potential false positives in the McPAS database, which were observed in almost all repertoires. Manually curated databases are subject to bias based on a priori knowledge and experimental design. Thus, our results provide further support for utilization of machine learning and other unbiased methodologies to classify T1D repertoires. (**C**) Sequence matches were calculated for different antigen species in the VDJdb database. **(D)** Association of 25,508 unique CDR3β sequences associated with cytomegalovirus (CMV) exposure with clinical groups. In the figure, multiple testing was performed using the Kruskal-Wallis test (denoted with red stars) and p-values were adjusted between pathology/antigen species. p-values for pairwise testing were calculated using two tailed Mann-Whitney U test and p-values were adjusted between different clinical groups. All p-values were adjusted for multiple testing using the Benjamini–Hochberg method. p-values were described as * for [0.01,0.05], ** for [0.001,0.01] and *** for <0.001 and no stars plotted for non-significant values. The cluster plot was generated using the UPGMA clustering method and Euclidean distance matrix. A universal color scheme was used to show type 1 diabetes (T1D), first degree relatives (FDR), second degree relatives (SDR) and control (CTRL) in red, yellow, light green and dark green, respectively.

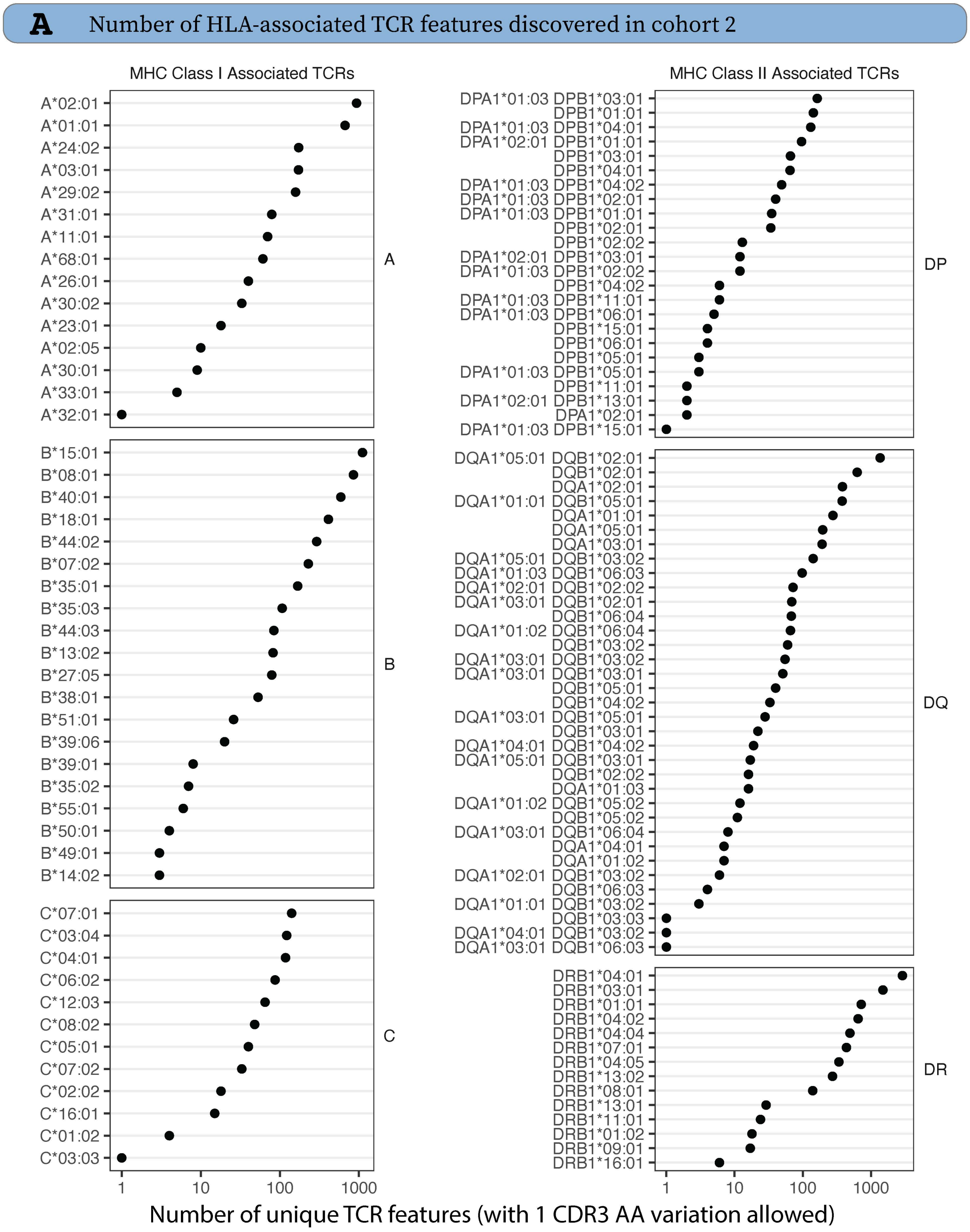

Fig. S4 | **Number of HLA-associated TCR features discovered in cohort 2**. A TCR was hypothesized as HLA-associated if its V-family constrained exact CDR3 match or edit-distance 1 CDR3 match was overrepresented in repertoires of persons with a particular HLA allele or haplotype (p-value < 1e-8 and prevalence within HLA-matched group > 5%, and prevalence in HLA-mismatched repertoires < 10%. Relates to **Fig. 3**.

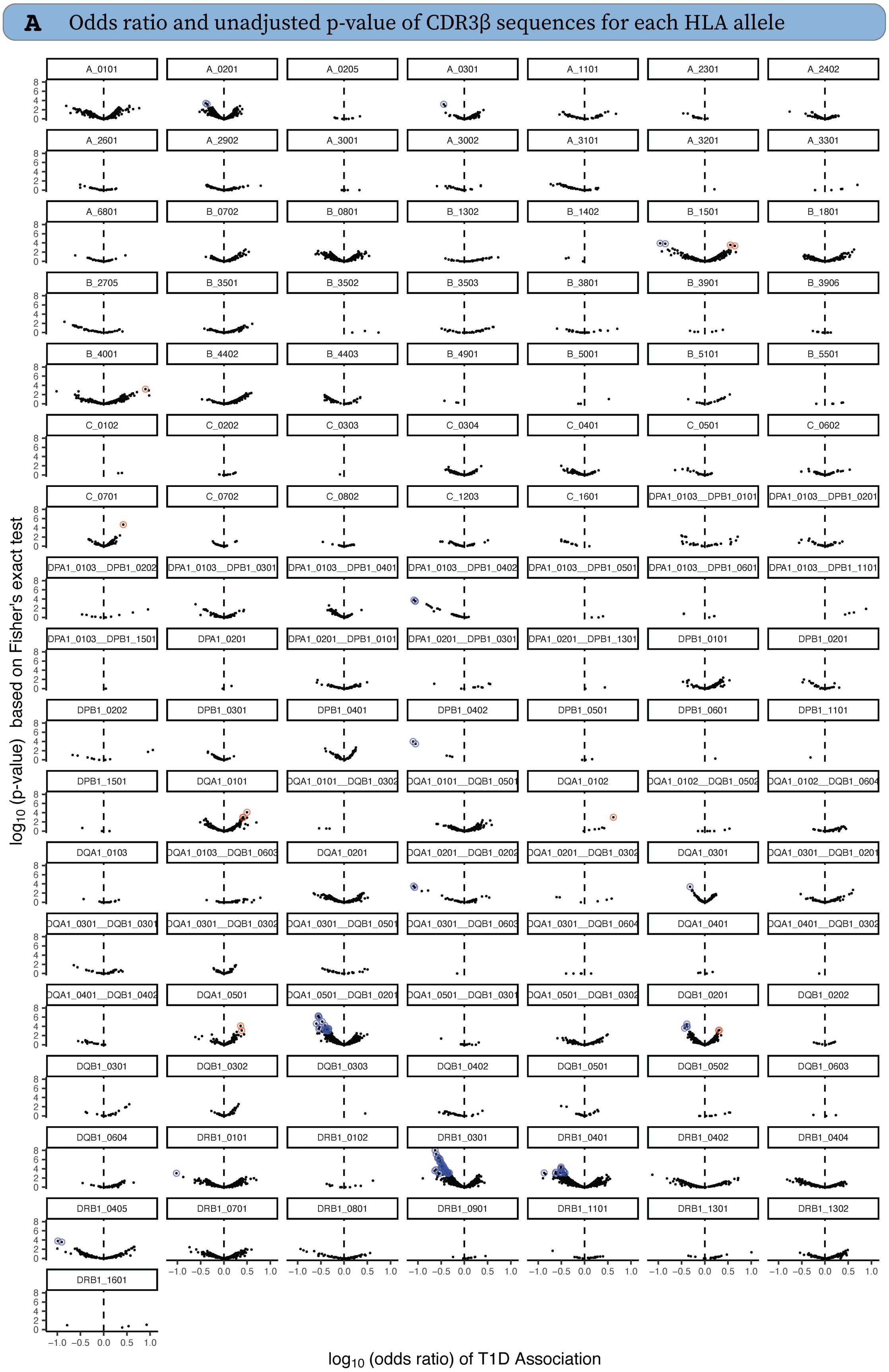
A
Odds ratio and unadjusted p-value of CDR3β sequences for each HLA allele
A_0101
A_0201
A_0205
A_0301
A_1101
A_2301
A_2402
A_2601
A_2902
A_3001
A_3002
A_3101
A_3201
A_3301
A_6801
B_0702
B_0801
B_1302
B_1402
B_1501
B_1801
B_2705
B_3501
B_3502
B_3503
B_3801
B_3901
B_3906
B_4001
B_4402
B_4403
B_4901
B_5001
B_5101
B_5501
C_0102
C_0202
C_0303
C_0304
C_0401
C_0501
C_0602
C_0701
C_0702
C_0802
C_1203
C_1601
DPA1_0103__DPB1_0101
DPA1_0103__DPB1_0201
DPA1_0103__DPB1_0202
DPA1_0103__DPB1_0301
DPA1_0103__DPB1_0401
DPA1_0103__DPB1_0402
DPA1_0103__DPB1_0501
DPA1_0103__DPB1_0601
DPA1_0103__DPB1_1101
DPA1_0103__DPB1_1501
DPA1_0201
DPA1_0201__DPB1_0101
DPA1_0201__DPB1_0301
DPA1_0201__DPB1_1301
DPB1_0101
DPB1_0201
DPB1_0202
DPB1_0301
DPB1_0401
DPB1_0402
DPB1_0501
DPB1_0601
DPB1_1101
DPB1_1501
DQA1_0101
DQA1_0101__DQB1_0302
DQA1_0101__DQB1_0501
DQA1_0102
DQA1_0102__DQB1_0502
DQA1_0102__DQB1_0604
DQA1_0103
DQA1_0103__DQB1_0603
DQA1_0201
DQA1_0201__DQB1_0202
DQA1_0201__DQB1_0302
DQA1_0301
DQA1_0301__DQB1_0201
DQA1_0301__DQB1_0301
DQA1_0301__DQB1_0302
DQA1_0301__DQB1_0501
DQA1_0301__DQB1_0603
DQA1_0301__DQB1_0604
DQA1_0401
DQA1_0401__DQB1_0302
DQA1_0401__DQB1_0402
DQA1_0501
DQA1_0501__DQB1_0201
DQA1_0501__DQB1_0301
DQA1_0501__DQB1_0302
DQB1_0201
DQB1_0202
DQB1_0301
DQB1_0302
DQB1_0303
DQB1_0402
DQB1_0501
DQB1_0502
DQB1_0603
DQB1_0604
DRB1_0101
DRB1_0102
DRB1_0301
DRB1_0401
DRB1_0402
DRB1_0404
DRB1_0405
DRB1_0701
DRB1_0801
DRB1_0901
DRB1_1101
DRB1_1301
DRB1_1302
DRB1_1601
−1.0 −0.5 0.0 0.5 1.0
0 2 4 6 8
log10 (p-value) based on Fisher's exact test
log10 (odds ratio) of T1D Association

Fig. S5 | **HLA-conditioned enrichment or depletion of TCRs based on T1D clinical status.** TCRs were identified as HLA-associated based on odds ratio of detection in cohort 2. Subsequently we tabulated detection of these TCRs and their v-family constrained one mutation distance neighbors among individuals expressing the predicted restricting allele in cohort 1 and tested for enrichment or depletion based on T1D clinical status (as detailed in Table S3). Volcano plots show odds ratio of T1D association and unadjusted negative log10 p-values. TCRs with statistically significant T1D-association (Fisher's exact test, false discovery rate < .2) are shown as red (T1D-enriched) or blue (T1D-depleted) circles. Relates to **Fig. 3**.

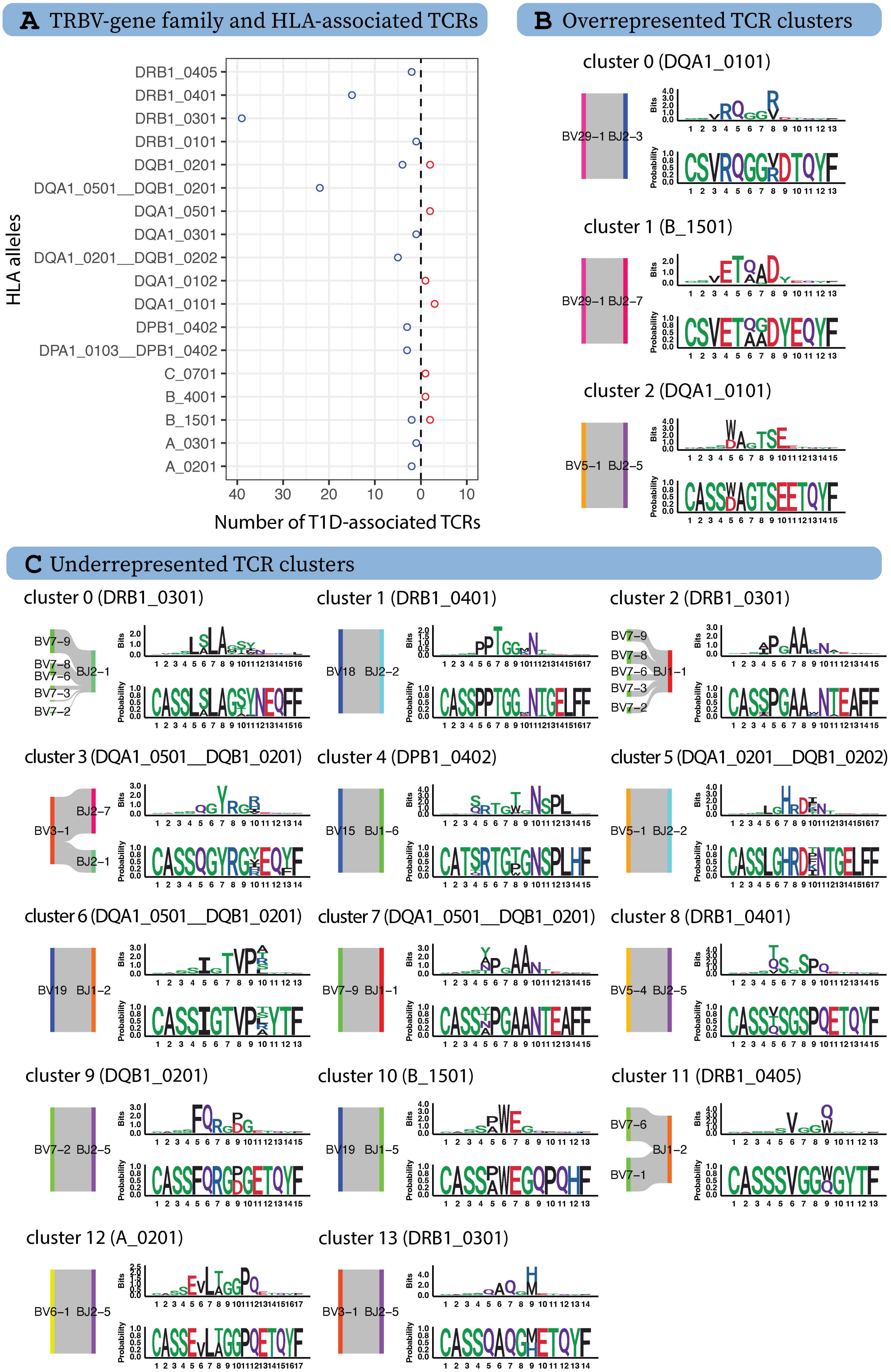
A TRBV-gene family and HLA-associated TCRs
B Overrepresented TCR clusters
C Underrepresented TCR clusters
HLA alleles
DRB1_0405
DRB1_0401
DRB1_0301
DRB1_0101
DQB1_0201
DQA1_0501__DQB1_0201
DQA1_0501
DQA1_0301
DQA1_0201__DQB1_0202
DQA1_0102
DQA1_0101
DPB1_0402
DPA1_0103__DPB1_0402
C_0701
B_4001
B_1501
A_0301
A_0201
40 30 20 10 0 10
Number of T1D-associated TCRs
cluster 0 (DQA1_0101)
BV29−1 BJ2−3
CSVRQGGVDTQYF
cluster 1 (B_1501)
BV29−1 BJ2−7
cluster 2 (DQA1_0101)
BV5−1 BJ2−5
cluster 0 (DRB1_0301)
BV7−9 BV7−8 BV7−6 BV7−3 BV7−2 BJ2−1
CASSLSLAGSYNEQFF
cluster 1 (DRB1_0401)
BV18 BJ2−2
CASSPPTGG_NTGELFF
cluster 2 (DRB1_0301)
BV7−9 BV7−8 BV7−6 BV7−3 BV7−2 BJ1−1
CASSPGAA_NTEAFF
cluster 3 (DQA1_0501__DQB1_0201)
BV3−1 BJ2−7 BJ2−1
cluster 4 (DPB1_0402)
BV15 BJ1−6
cluster 5 (DQA1_0201__DQB1_0202)
BV5−1 BJ2−2
cluster 6 (DQA1_0501__DQB1_0201)
BV19 BJ1−2
cluster 7 (DQA1_0501__DQB1_0201)
BV7−9 BJ1−1
cluster 8 (DRB1_0401)
BV5−4 BJ2−5
cluster 9 (DQB1_0201)
BV7−2 BJ2−5
cluster 10 (B_1501)
BV19 BJ1−5
cluster 11 (DRB1_0405)
BV7−6 BV7−1 BJ1−2
CASSSVGGWGYTF
cluster 12 (A_0201)
BV6−1 BJ2−5
cluster 13 (DRB1_0301)
BV3−1 BJ2−5
CASSQAQGMETQYF
Bits
Probability

Fig. S6 | **T1D-association of TCR features observed based on assessment of HLA-associated, TRBV-gene family constrained and single amino acid mutation distance neighbor CDR3β sequences.** (**A**) TRBV-gene family and HLA-associated CDR3β sequences depleted (blue) or overrepresented (red) in the T1D repertoires (false discovery rate < 0.2), when considering detection of a CDR3 or a nearly-identical CDR3 with only one indel or substitution. (**B**) Gene usage and CDR3β(AA) motifs formed from top sequence clusters overrepresented in T1D repertoires. **(C**) Gene usage and CDR3β(AA) motifs formed from top sequence clusters underrepresented in T1D repertoires. HLA association determined by Fisher Exact Test p-value < 1E-8, HLA-prevalence > 0.05, non-HLA-prevalence < 0.1 in Cohort2. Relates to **Fig. 3**.

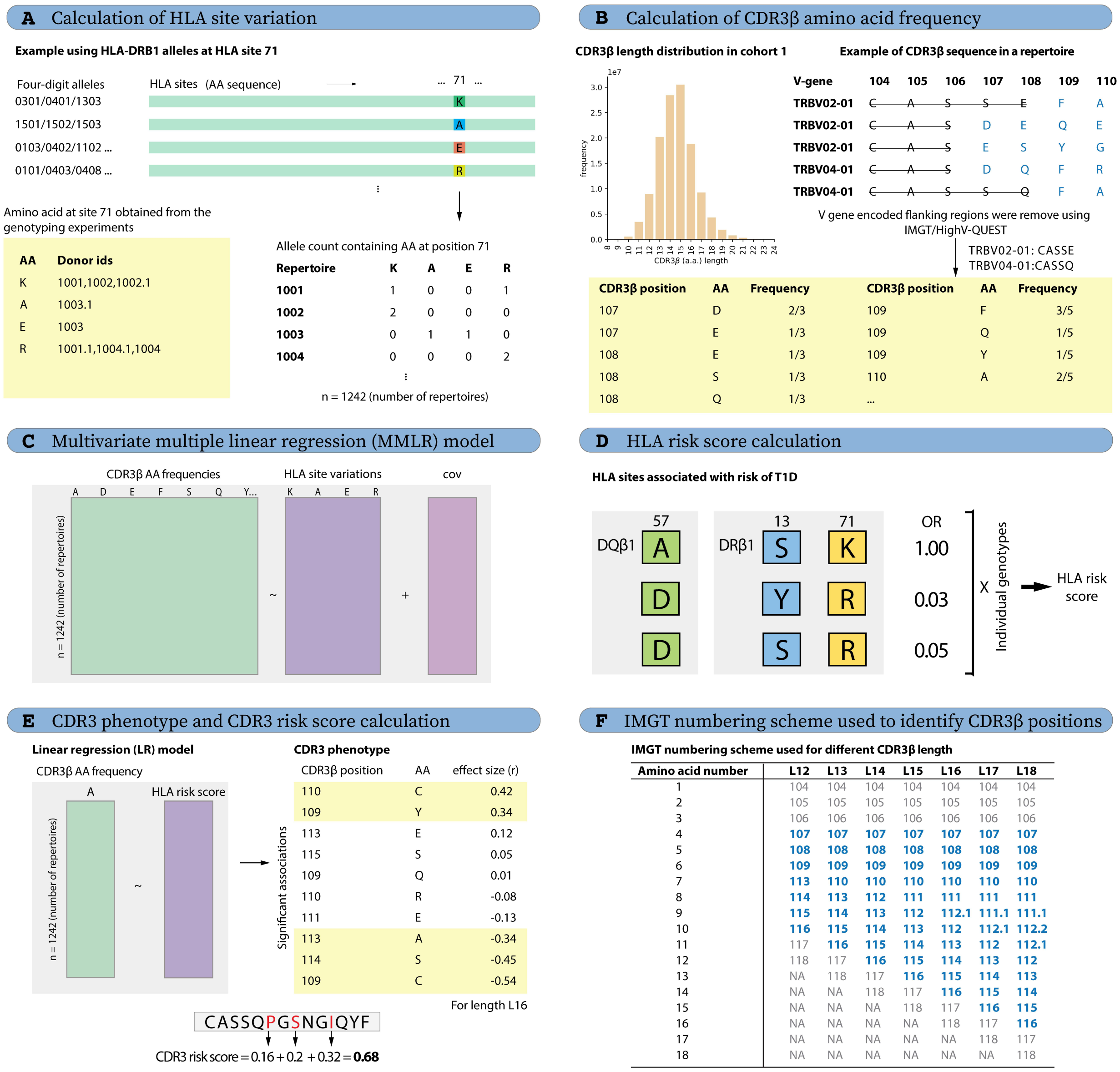

Fig. S7 | The methodology used to assess the association between HLA and CDR3β. (A) The information related to amino acid (AA) polymorphism present at the HLA site was obtained from the HLA genotyping experiments. Here, HLA-DRB1 is given as an example, which has 4 AA variants (K, A, E, R) at site 71. All possible AA variant counts were calculated for each repertoire. **(B)** The histogram (left) shows the distribution of the CDR3β length in cohort 1 (for lengths containing more than 30,000 CDR3β sequences). The position-specific AA frequency was calculated for each CDR3β sequence with length ranging from L12 to L18 after removing the V- and J-germline-encoded flanking regions (right). In the example, we have removed the N-terminal sequences based on specific V-genes ("CASSE" for TRBV02-01 and "CASSQ" for TRBV04-01). The V and J germline-encoded sequences were removed using IMGT/HighV-QUEST tool. Further, the frequency of all AAs was calculated for each position and for each length (L12-L18) of CDR3β sequences. **(C)** The multivariate multiple linear regression model (MMLR) was calculated for each HLA site polymorphism, where count of AA variants at the site was explanatory variable and AA frequency of specific CDR3β position was response variable. This was done for each IMGT position of CDR3β sequence for all lengths (L12-L18), recursively. **(D)** The HLA risk score was calculated based on the 3 AA positions: HLA-DQβ1 (site 57) and HLA-DRβ1 (site 13 and 71). The odds ratio (OR) for possible combinations of three AAs was provided in a previous study (*79*), which was multiplied by the individual's genotype. In case multiple combinations were possible for the three AAs, then we consider the combination with the highest OR score. **(E)** In the first step to calculate CDR3 risk score, we considered HLA risk score and position-specific frequency of one AA to develop several linear regression models (LR) for each CDR3β position and length, recursively. The p-values were further adjusted for multiple testing using the Benjamini–Hochberg method. The AAs which showed significant correlations with HLA risk score (p-value≤0.05) were considered "T1D-associated position-specific

CDR3β AA" or "CDR3 phenotypes" and respective correlation coefficients were considered the "effect sizes" associated with T1D. Within a repertoire, the effect sizes of each CDR3 phenotype were summed up for the whole CDR3β sequence, and averaged for the whole repertoire to calculate CDR3 risk score. **(F)** In our study, the CDR3β positions for CDR3β length L12 to L18 were defined using the IMGT numbering scheme, which are highlighted in blue color in the figure. The examples in the figures are taken directly or inspired from Iskigaki et al (*7*). Relates to **Fig. 3**.

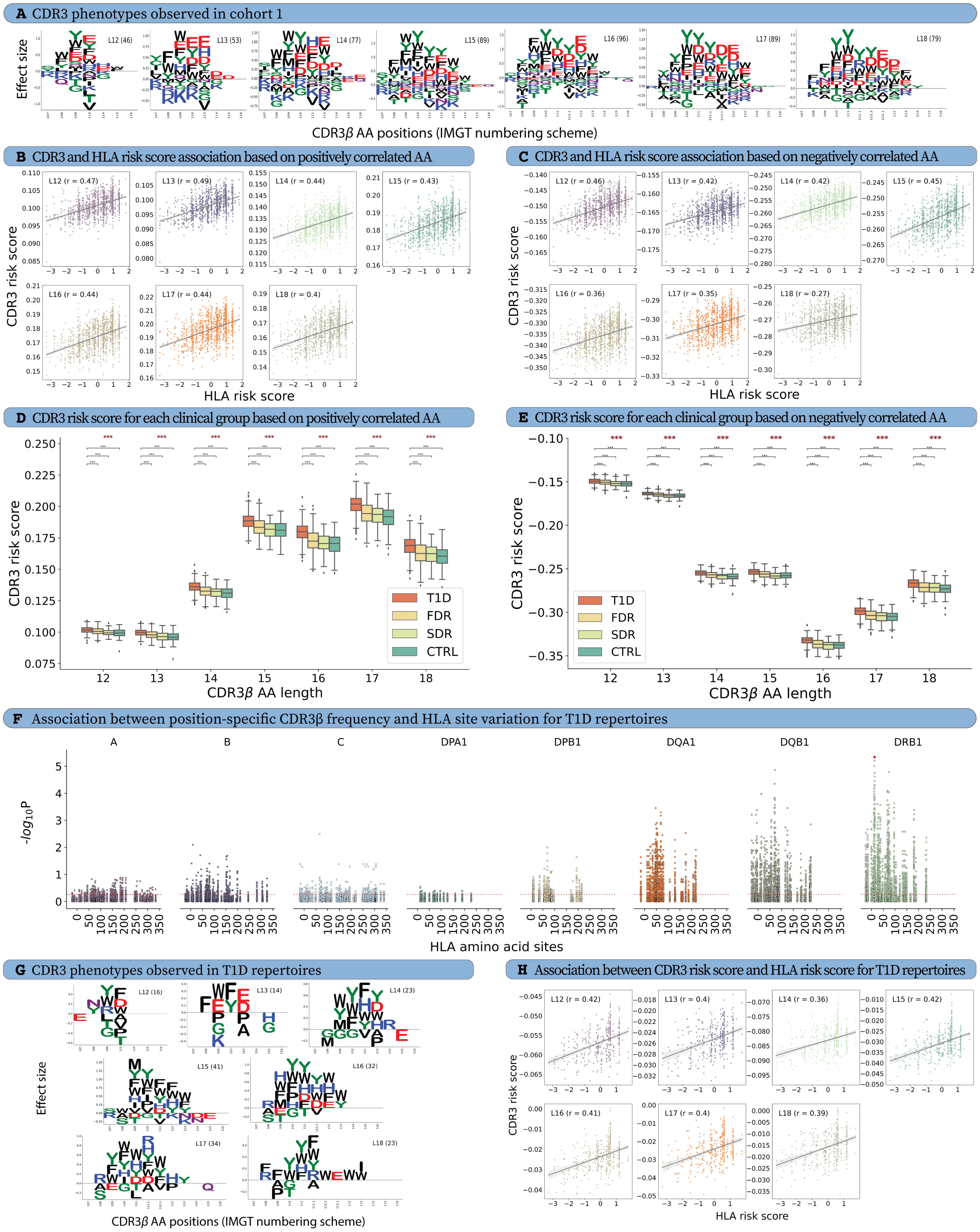

Fig. S8 | **Assessment of positively- and negatively-associated CDR3 phenotypes obtained from cohort 1 and HLA-based restriction of CDR3β sequences in T1D clinical group of cohort 1. (A)** The sequence logo plot of CDR phenotypes and their effect sizes (coefficients), obtained from the LR analysis of each CDR3β length (L12-L18), using cohort 1. In cohort 1, there were 304 positive and 225 negative significant (p-value≤0.05) associations present between CDR3 phenotypes and HLA risk score, for all CDR3β lengths. We calculated the correlation with HLA risk score when CDR3 risk score was calculated using **(B)**

positively-associated CDR3 phenotypes and **(C)** negatively-associated CDR3 phenotypes. CDR3 risk score calculated using **(D)** positively-associated and **(E)** negatively-associated CDR3 phenotypes also showed association with clinical groups and observed the expected trend, where T1D>FDR>SDR>CTRL. **(F)** The MANOVA test p-values from the MMLR analysis of the 402 T1D repertoires, where all CDR3β positions were plotted for each HLA site containing mutation(s). The p-values (≤0.05) above the red line were significant. The lowest p-value observed for cohort 1 (HLA-DRB1 site 13 with CDR3β position 111 for L15) is mapped onto the MANOVA test p-values and highlighted in red color. **(G)** The logo plot represents CDR3 phenotypes and their effect size (coefficient) obtained from the LR analysis using T1D clinical group in cohort 1. **(H)** The correlation between HLA risk score and CDR3 risk scores calculated for T1D repertoires, for each CDR3β length (L12-L18). The correlation (r) values were shown for each length in appropriate plots. Multiple testing was performed using the Kruskal-Wallis test (denoted with red stars) and p-values were adjusted between different CDR3β lengths. p-values for pairwise testing were calculated using two tailed Mann-Whitney U tests and p-values were adjusted between clinical groups. All p-values were adjusted for multiple testing using the Benjamini–Hochberg method. p-values were described as * for [0.01,0.05], ** for [0.001,0.01] and *** for <0.001 and no stars plotted for non-significant values. Relates to **Fig. 3**.

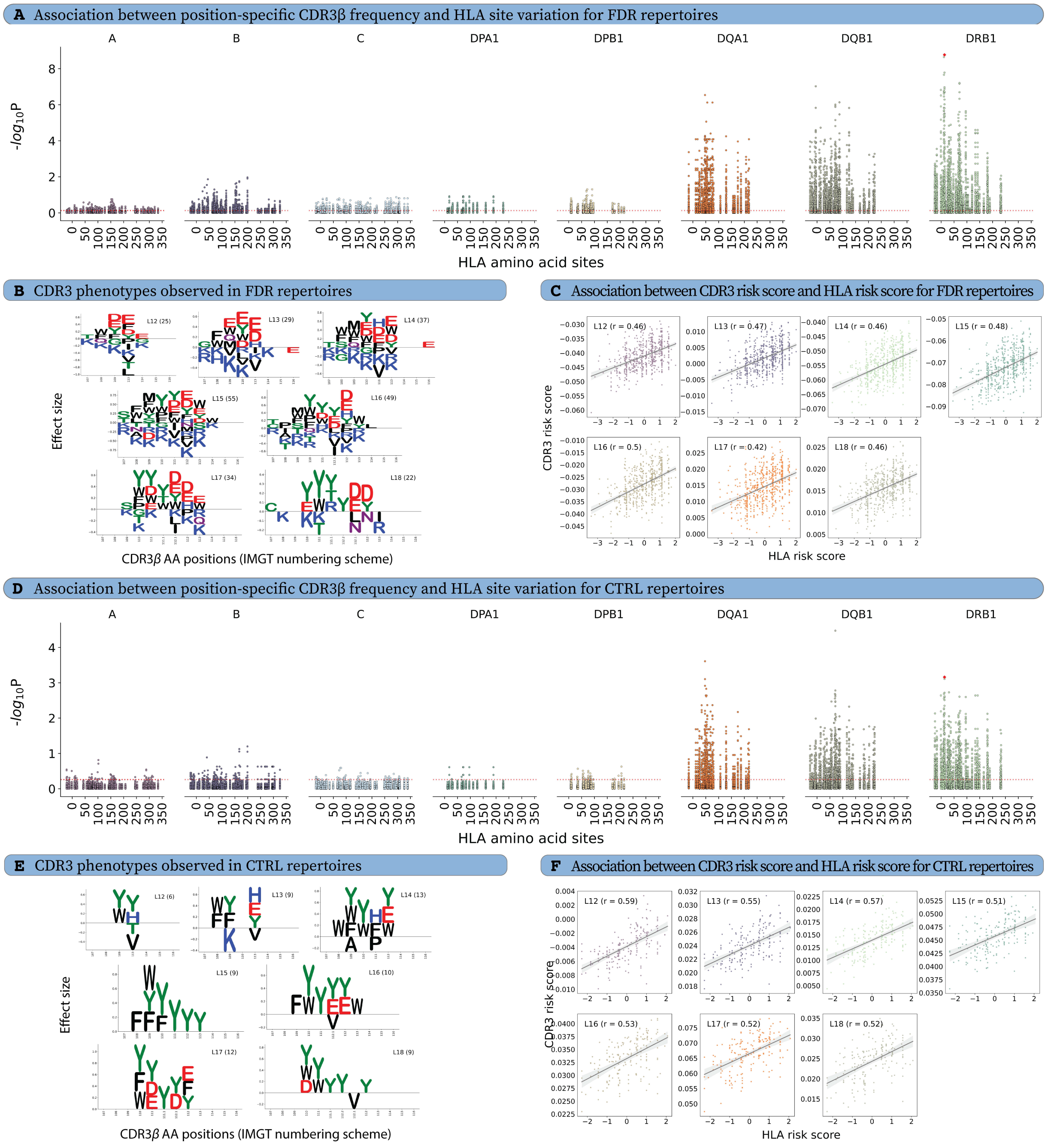

Fig. S9 | Assessment of HLA-based restriction of CDR3β sequences in FDR (first degree relatives) and CTRL (control) groups of cohort 1. The MANOVA test p-values from the MMLR analysis of the **(A)** 601 FDR and **(D)** 182 CTRL repertoires, where all CDR3β positions were plotted for each HLA site containing mutation(s). The p-values (≤0.05) above the red line were significant. The logo plots represent CDR3 phenotypes and their effect sizes (coefficient) obtained from the LR analysis using **(B)** FDR and **(E)** CTRL repertoires. The correlation between HLA risk score and CDR3 risk scores calculated for **(C)** FDR and **(F)** CTRL repertoires, for each CDR3β length (L12-L18). The correlation (r) values were shown for each length in appropriate plots. The lowest p-value observed for cohort 1 (HLA-DRB1 site 13 with CDR3β position 111 for L15) is mapped onto the MANOVA test p-values and highlighted in red color. Relates to Fig. 3.

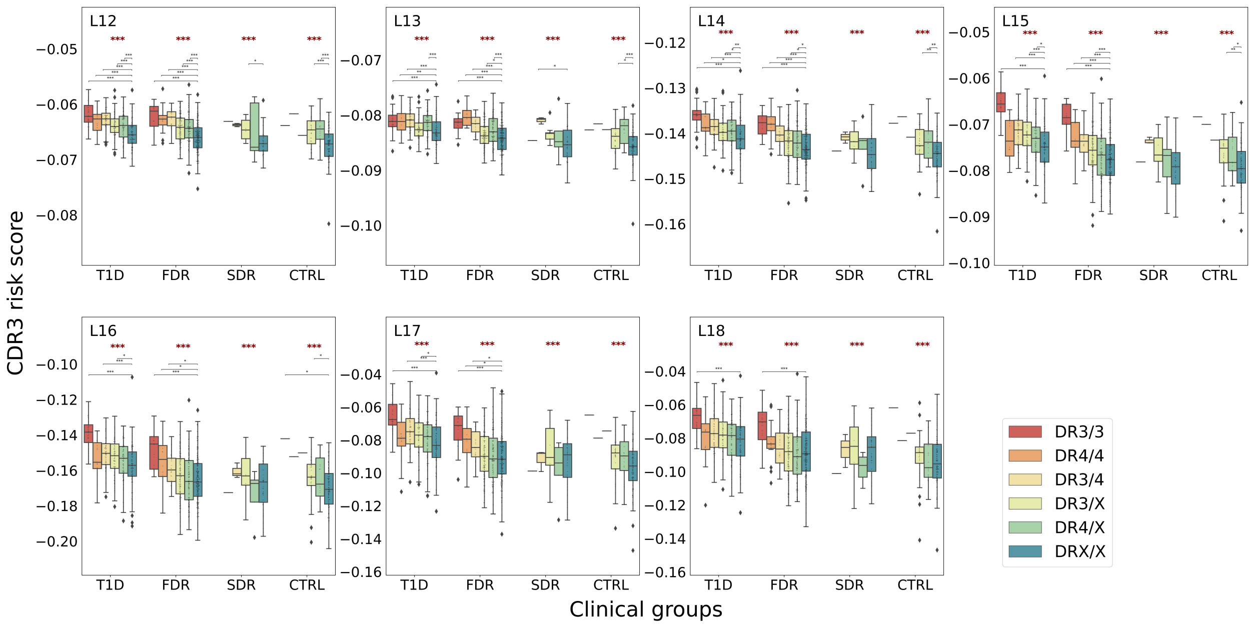

Fig. S10 | CDR3 risk score shows association with conventional T1D-linked high-risk HLA alleles (DR3 and DR4) at different CDR3β lengths within clinical groups, in cohort 1. The CDR3 risk score was higher in individuals containing at least one high-risk HLA allele regardless of clinical groups. In the figure, multiple testing was performed using the Kruskal-Wallis test (denoted with red stars) and p-values were adjusted between different clinical groups. p-values for pairwise testing were calculated using two tailed Mann-Whitney U tests and p-values were adjusted between different high-risk HLA alleles. All p-values were adjusted for multiple testing using the Benjamini–Hochberg method. p-values were described as * for [0.01,0.05], ** for [0.001,0.01] and *** for <0.001 and no stars plotted for non-significant values. Relates to **Fig. 3**.

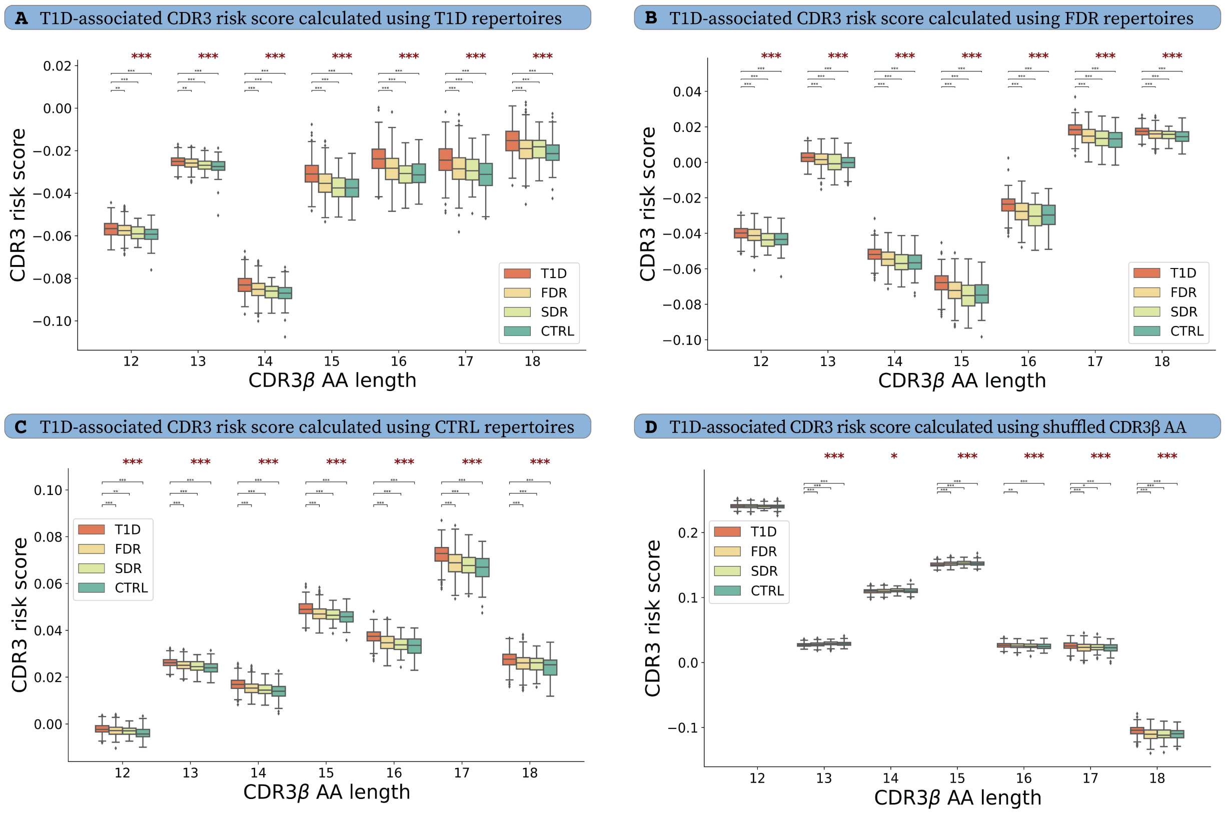

Fig. S11 | The association between CDR3 risk scores and clinical groups exhibits a high level of consistency and robustness, suggesting a strong and reliable association. The CDR3 phenotypes obtained from each clinical group were further used to calculate the CDR3 risk score for all clinical groups. Further, CDR3 risk score was clustered based on clinical groups, where CDR3 phenotypes were identified using **(A)** T1D **(B)** FDR and **(C)** CTRL clinical groups. **(D)** As a validation test, we shuffled the effect sizes (LR coefficients) of the CDR3 phenotypes obtained from cohort 1 to calculate the CDR3 risk score. The observed trend, where T1D>FDR>SDR>CTRL was lost upon shuffling the effect sizes of CDR3 phenotypes. In the figure, multiple testing was performed using the Kruskal-Wallis test (denoted with red stars) and p-values were adjusted between different CDR3β lengths. p-values for pairwise testing were calculated using two tailed Mann-Whitney U tests and p-values were adjusted between different clinical groups. All p-values were adjusted for multiple testing using the Benjamini–Hochberg method. p-values were described as * for [0.01,0.05], ** for [0.001,0.01] and *** for <0.001 and no stars plotted for non-significant values. Relates to **Fig. 3**.

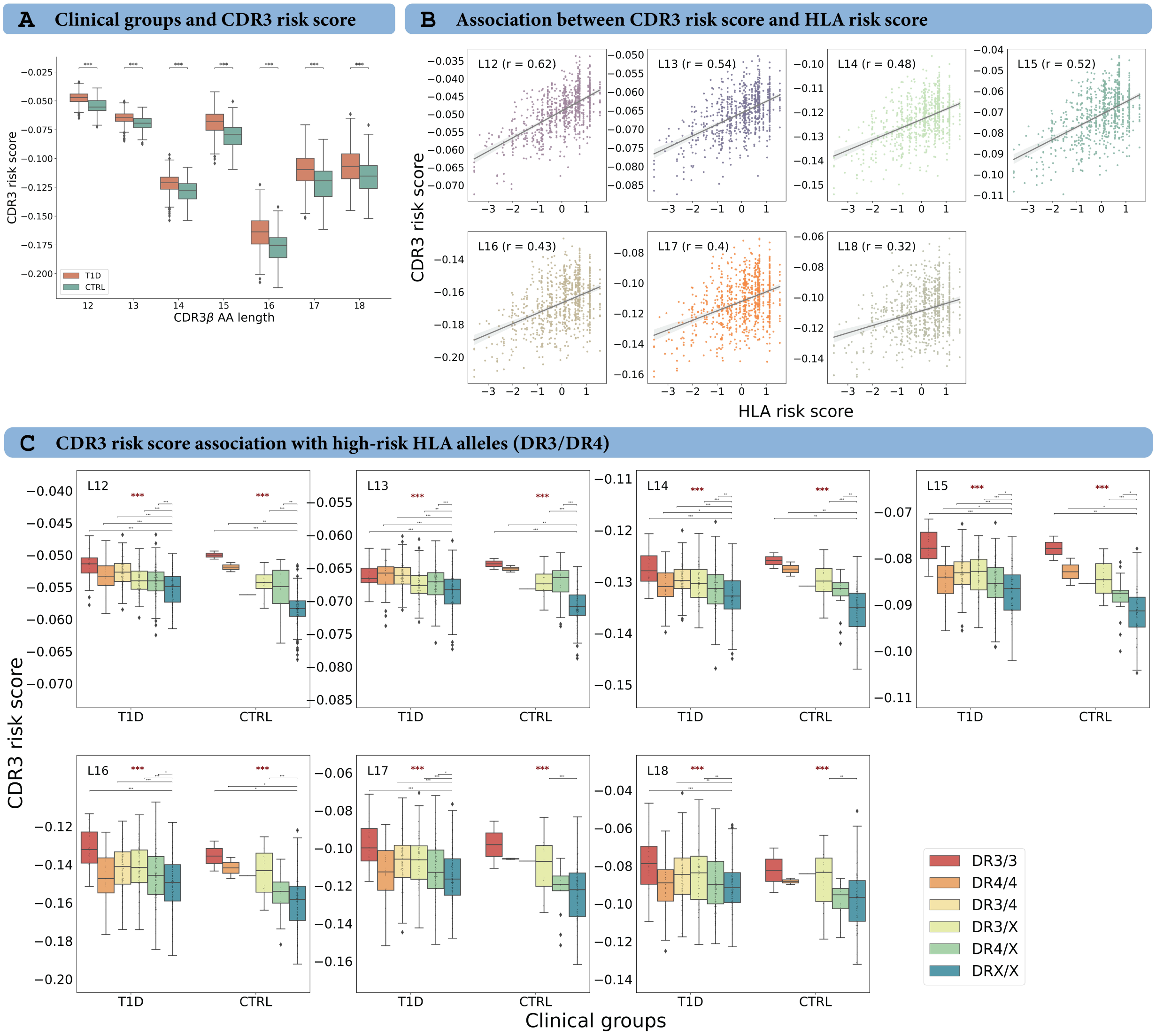

Fig. S12 | Assessment of CDR3 risk score calculated on cohort 2 and 3 based on the CDR3 phenotypes obtained from cohort 1. The CDR3 risk score calculated on cohort 2 and 3 **(A)** showed association with T1D and CTRL clinical groups, where T1D>CTRL; and **(B)** showed positive correlation with HLA risk score for different CDR3β length (L12-L18). **(C)** CDR3 risk score also showed association with presence of high-risk HLA alleles (DR3 and DR4) in cohort 2 and 3, when clustered by clinical groups. In figure **panel C**, multiple testing was performed using the Kruskal-Wallis test (denoted with red stars) and p-values were adjusted between different clinical groups. p-values for pairwise testing were calculated using two tailed Mann-Whitney U tests and p-values were adjusted between different high-risk HLA alleles (and between clinical groups in **panel A**). All p-values were adjusted for multiple testing using the Benjamini–Hochberg method. p-values were described as * for [0.01,0.05], ** for [0.001,0.01] and *** for <0.001 and no stars plotted for non-significant values. Relates to Fig. 3.

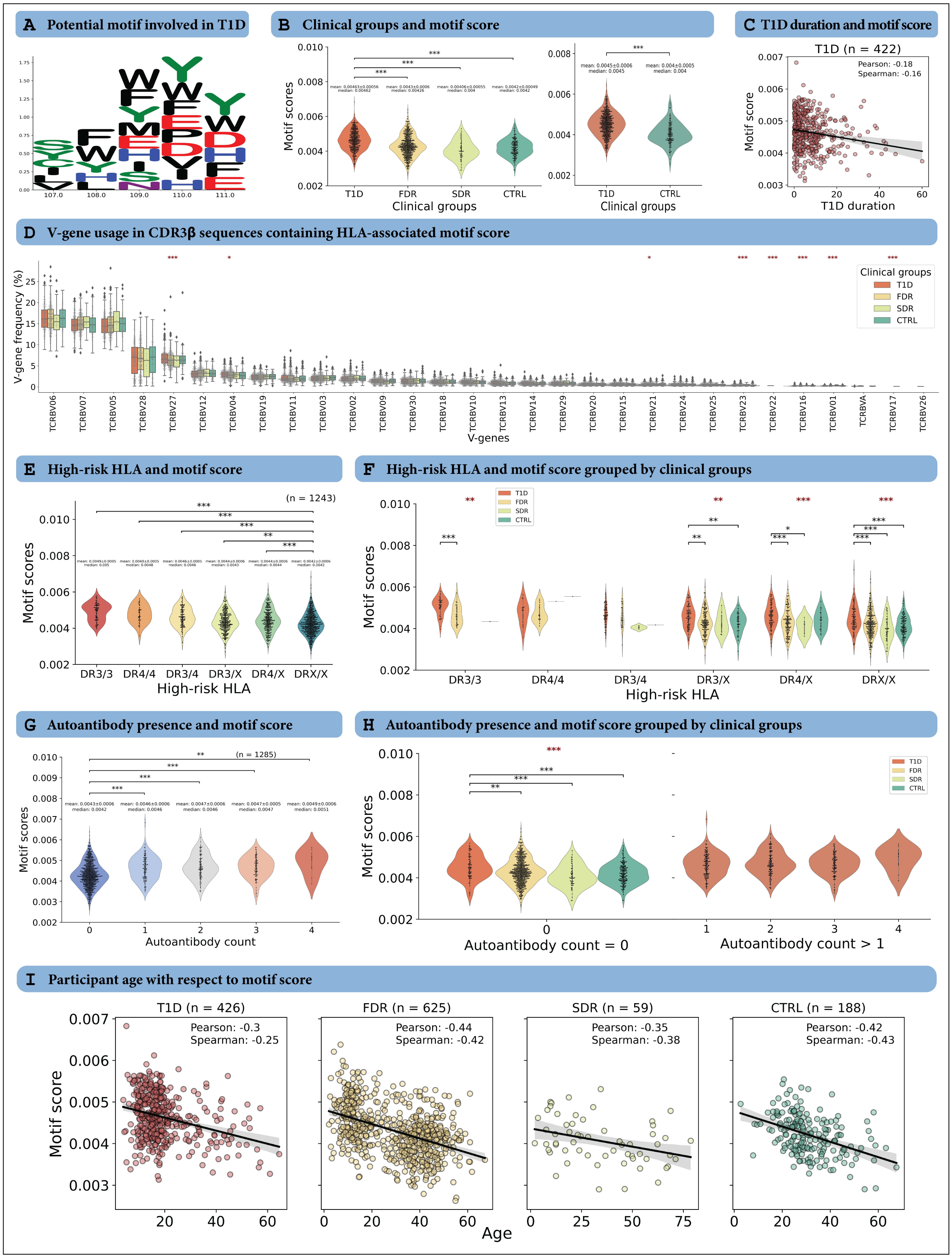

Fig. S13 | The assessment of the pHLA-motif, derived from the HLA risk score, shows positive association with the clinical groups, high-risk HLA alleles (DR3/DR4) and autoantibody presence. (A) A sequence logo representation of the

pHLA-motif**.** The consensus motif was derived from the CDR3β lengths L13-16 and IMGT positions 107-111. **(B)** The motif score was higher for T1D repertoires in cohort 1, following the expected trend of T1D > FDR > SDR > CTRL, with SDR being an exception. A similar pattern was also observed in cohorts 2 and 3. The pHLA-motif score represents the number of CDR3β sequences containing the pHLA-motifs normalized by total number of CDR3β sequences per repertoire, where higher pHLA-motif score signifies higher risk of T1D. **(C)** The correlation between T1D duration and motif score shows a pearson correlation of -0.18 and spearman correlation of -0.16. **(D)** There was no trend observed in V-gene usage across TCRβ repertoires of different clinical groups, when considering the CDR3β sequences containing pHLA-motifs. **(E)** The pHLA-motif score was significantly higher in the high-risk HLA type (DR3 or DR4) individuals than other HLA types (DRX/X). The p-values in the figure were calculated with respect to the DRX/X HLA type. **(F)** The pHLA-motif score was plotted for each high-risk HLA type and grouped by the clinical groups.The pHLA-motif score observed the expected trend T1D>FDR>CTRL in all cases irrespective of high-risk HLA allele type. Here, pairwise testing was performed with respect to the T1D clinical group. **(G)** The pHLA-motif score also showed positive association with the number of autoantibodies present. The p-values were calculated with respect to no presence of autoantibodies. **(H)** The pHLA-motif score was high for autoantibody count >0. However, in case of no presence of autoantibody, motif score followed the expected trend where T1D>FDR>CTRL. **(I)** The pHLA-motif score was plotted against age of the individual for different clinical groups (for cohort 1). Each T1D status showed negative correlation with the age of the individual. In the figure, the p-values for the multiple testing were performed using the Kruskal-Wallis test (denoted with red stars). Similarly, p-values for pairwise testing were calculated using two tailed Mann-Whitney U tests. p-values were described as * for [0.01,0.05], ** for [0.001,0.01] and *** for <0.001 and no stars plotted for non-significant values. All p-values were adjusted for multiple testing using the Benjamini–Hochberg method. Relates to **Fig. 3**.

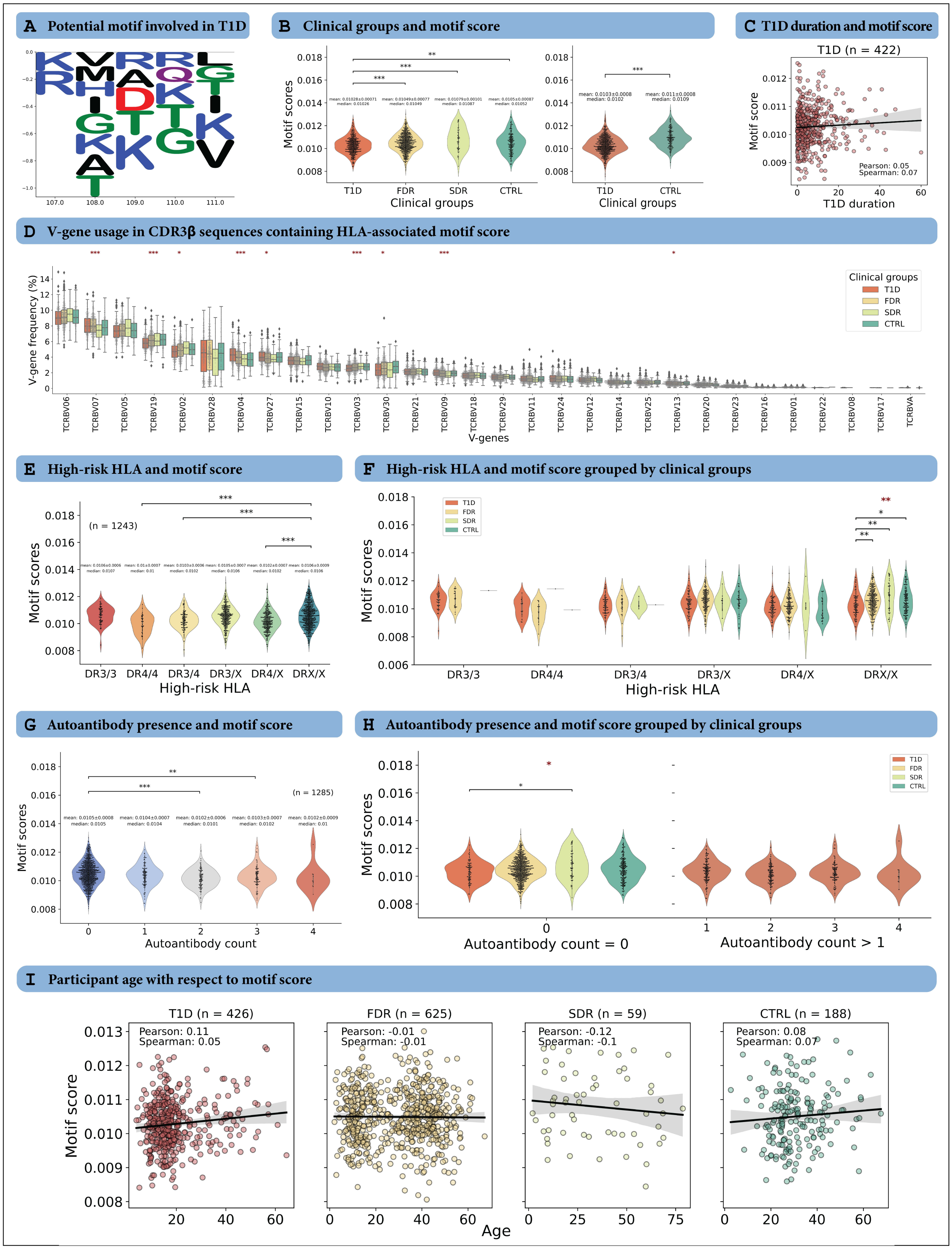

Fig. S14 | The assessment of the nHLA-motif (protective motif), derived from the HLA risk score, shows negative association with the clinical groups, high-risk HLA alleles (DR3/DR4) and autoantibody presence. (A) A sequence logo

representation of the nHLA-motif**.** The consensus motif was derived from the CDR3β lengths L13-16 and IMGT positions 107-111. **(B)** The motif score was higher for non-T1D repertoires in cohort 1, following the expected trend of T1D < FDR < SDR < CTRL, with SDR being an exception. A similar pattern was also observed in cohorts 2 and 3. The nHLA-motif score represents the number of CDR3β sequences containing the nHLA-motifs normalized by total number of CDR3β sequences per repertoire, where higher nHLA-motif score signifies lower risk of T1D. **(C)** The nHLA-motif score from the protective motif showed no correlation with the T1D duration (pearson correlation of 0.05 and spearman correlation of 0.07). **(D)** The V-genes with higher frequency had relatively lower usage in the T1D repertoires and had an observable trend of T1D<FDR<CTRL, when considering the CDR3β sequences containing the nHLA-motif. **(E)** The repertoires containing at least one DR3 allele exhibited nHLA-motif scores comparable to non-risk repertoires, indicating that DR3 alleles may possess more protective motifs. **(F)** The nHLA-motif score was plotted for each high-risk HLA type and grouped by the clinical groups. The nHLA-motif score was significantly higher for CTRL repertoires in cases both HLA alleles were not associated with high-risk of T1D (DRX/X) and observed the expected trend T1D<FDR<CTRL. Here, pairwise testing was performed with respect to the T1D clinical group. **(G)** The nHLA-motif score was higher for the repertoires with no or just one autoantibody present. The p-values were calculated with respect to no autoantibody presence. **(H)** The nHLA-motif score observed association with clinical groups in repertoires with no autoantibody presence with an expected trend where T1D<FDR<CTRL. **(I)** The nHLA-motif score was plotted against age of the individual for different clinical groups (for cohort 1). No correlation was observed for the nHLA-motif score with age of the individual. In the figure, the p-values for the multiple testing were performed using the Kruskal-Wallis test (denoted with red stars). Similarly, p-values for pairwise testing were calculated using two tailed Mann-Whitney U tests. p-values were described as * for [0.01,0.05], ** for [0.001,0.01] and *** for <0.001 and no stars plotted for non-significant values. All p-values were adjusted for multiple testing using the Benjamini–Hochberg method. Relates to **Fig. 3**.

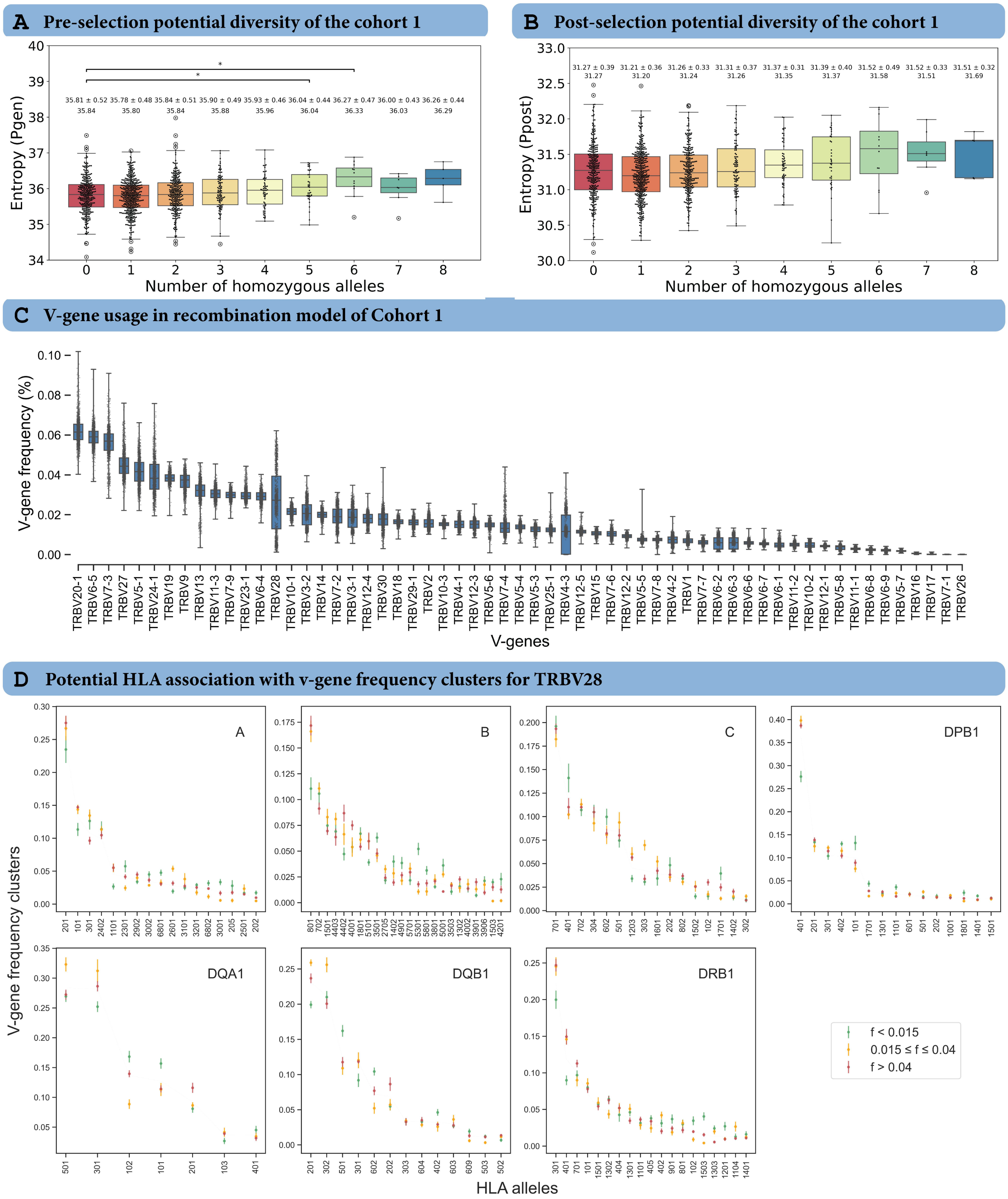

Fig. S15 | **Heterozygous HLA alleles showed restriction of the TCR repertoire diversity in cohort 1. (A)** The pre-selection TCR repertoire diversity (Pgen, IGoR) and **(B)** post-selection repertoire diversity (Ppost, SoNNia) calculated with respect to count of homozygous HLA alleles for 8 HLA genes (A, B, C, DPA1, DPB1, DQA1, DQB1, DRB1) showed a trend where repertoires with more heterozygous HLA alleles had lower diversity in both pre and post selection. **(C)** V-gene usage frequency calculated from the IGoR model. Some of the V-genes including TRBV28, TRBV4-3, TRBV3-2, TRBV3-1 showed clustering of v-gene frequency. **(D)** as a case study, the three v-gene frequency clusters of TRBV28 were assessed for their potential link with

the HLA genes. The HLA alleles with frequency >0.01 were considered in the plot. The error bars were plotted by bootstrapping samples, where 30% of the data resampled multiple times. In figure **A** and **B**, p-values for pairwise testing were calculated using two tailed Mann-Whitney U tests with respect to most heterozygous HLA individuals (number of homozygous alleles = 0). p-values were described as * for [0.01,0.05], ** for [0.001,0.01] and *** for <0.001 and no stars plotted for non-significant values. All p-values were adjusted for multiple testing using the Benjamini–Hochberg method.

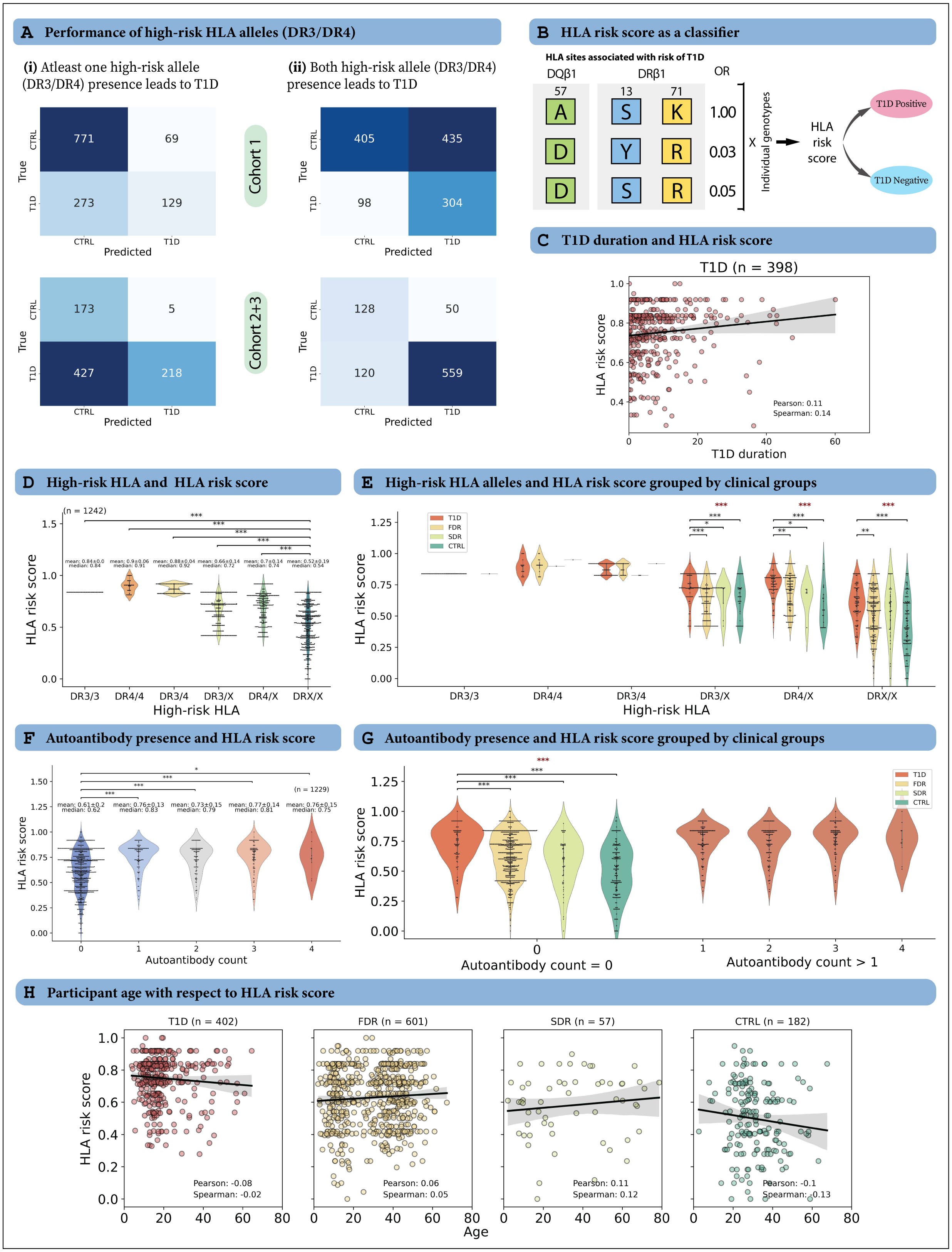

Fig. S16 | Assessment of HLA risk score demonstrates that HLA alleles are robust predictors of T1D status (A) Confusion matrices were generated using high-risk HLA allele types (DR3 and DR4) as predictor of T1D status under two scenarios: (i)

considering the presence of at least one high-risk HLA allele as indication of T1D status, and (ii) considering the presence of both high-risk HLA allele as indication of T1D status. The confusion matrices were generated separately for cohort 1 and cohort 2+3 (see **Supplementary Note** for more detail). **(B)** The HLA risk score was calculated based on the 3 AA positions: HLA-DQβ1 (site 57) and HLA-DRβ1 (site 13 and 71). Further, HLA risk score was used for binary classification of T1D positive and negative repertoires. **(C)** Expectedly, T1D duration does not show correlation with HLA risk score (pearson correlation: 0.11 and spearman correlation: 0.14). **(D)** HLA risk score in the repertoires with high-risk HLA alleles type (DR3 or DR4) was significantly higher compared to other HLA types (DRX/X). The p-values in the figure were calculated with respect to the DRX/X HLA type. **(E)** The HLA risk score was plotted for each high-risk HLA type and grouped by the clinical groups. The HLA risk score was significantly high in all clinical groups containing both alleles associated with high-risk (DR3 and DR4). However, it observed the expected trend T1D>FDR>SDR>CTRL in cases where at least one HLA allele was not associated with high-risk of T1D. Here, pairwise testing was performed with respect to the T1D clinical group. **(F)** The HLA risk score also observed positive association with the number of autoantibodies present. The p-values were calculated with respect to no presence of autoantibodies. **(G)** HLA risk score was high for all individuals when autoantibody count was >0. However, in case of no presence of autoantibody, HLA risk score followed the expected trend where T1D>FDR>SDR>CTRL. **(H)** The HLA risk score was plotted against age of the individual for different clinical groups (for cohort 1) and as expected, it did not show any correlation with the age of the individual. In the figure, the p-values for the multiple testing were performed using the Kruskal-Wallis test (denoted with red stars). Similarly, p-values for pairwise testing were calculated using two tailed Mann-Whitney U tests. p-values were described as * for [0.01,0.05], ** for [0.001,0.01] and *** for <0.001 and no stars plotted for non-significant values. All p-values were adjusted for multiple testing using the Benjamini–Hochberg method. Relates to **Fig. 4**.

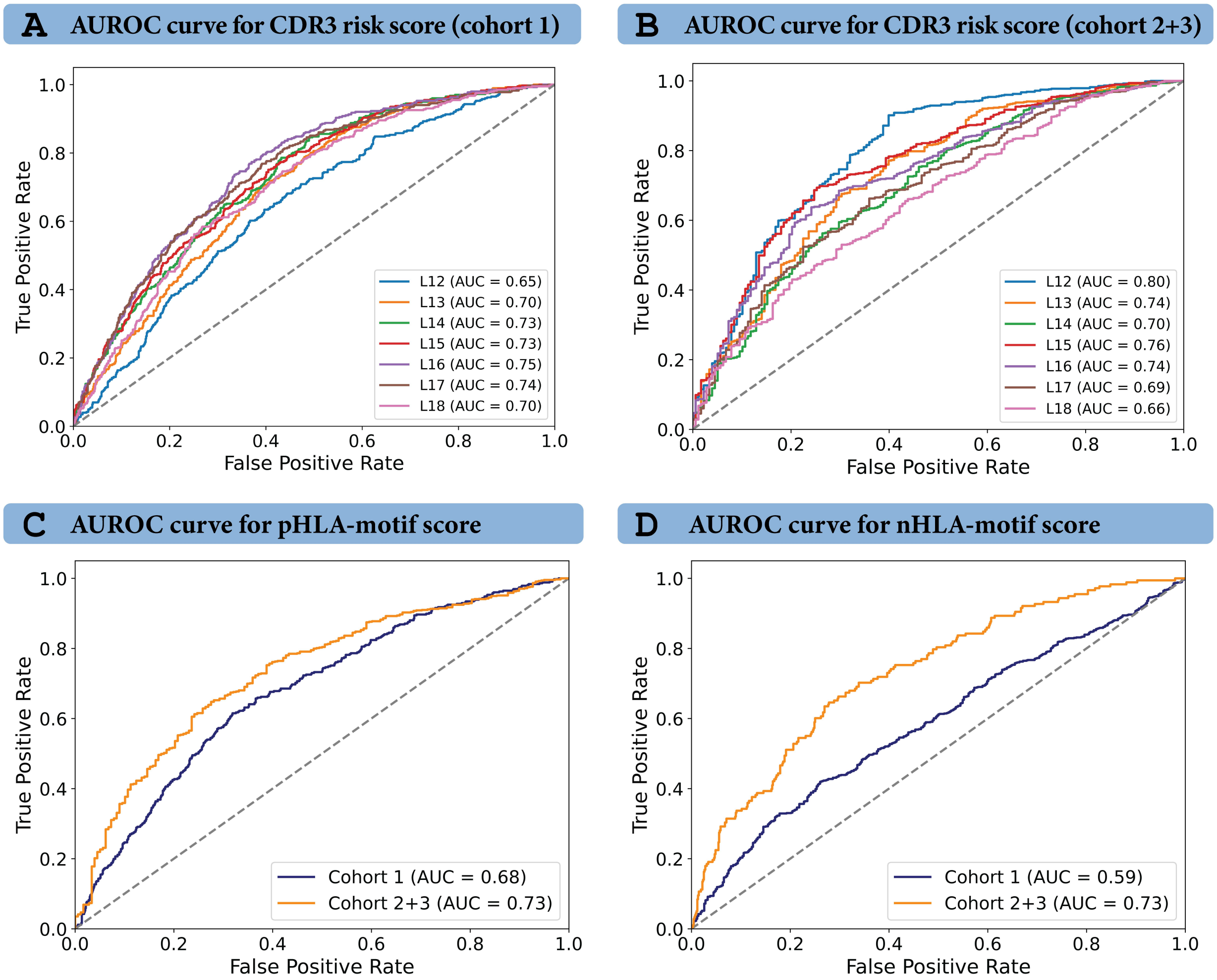

Fig. S17 | CDR3 risk scores and HLA-associated motif scores derived from the TCR repertoire (TCR features) in the HLA-based restriction study demonstrate good performance in classification of T1D and non-T1D repertoires. The AUROC curves for classification were plotted for **(A)** CDR3 risk score calculated for each CDR3β length (L12-L18) for cohort 1, **(B)** CDR3 risk score calculated for cohort 2 and 3 using the CDR3 phenotypes obtained from cohort 1, **(C)** pHLA-motif score and (D) nHLA-motif score. Relates to **Fig. 4**.

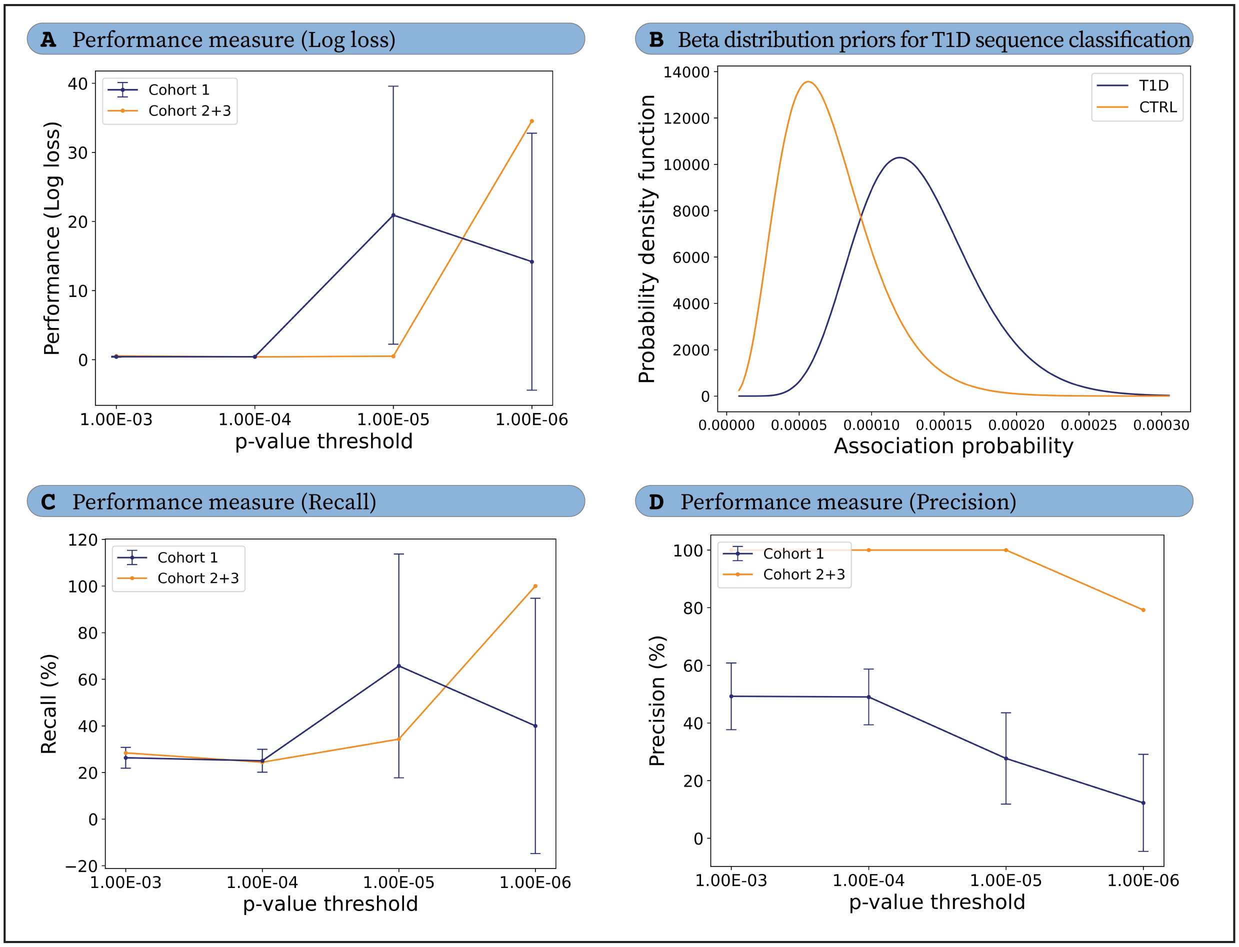

Fig. S18 | **The performance of Emerson's statistical classification framework based on public clones demonstrated high variability in the results on the training cohort 1 and test cohorts 2+3, at different selection thresholds. (A)** The classification of TCR repertoires was optimized using the Log loss function. **(B)** The beta-distribution priors for T1D and CTRL classes demonstrated poor class separation. **(C)** The recall for the model was low for all threshold values. **(D)** The precision for the model was low for cohort 1 but unexpectedly high for cohort 2+3 (potentially due to high sequencing depth in cohort 3). Relates to **Fig. 4**.

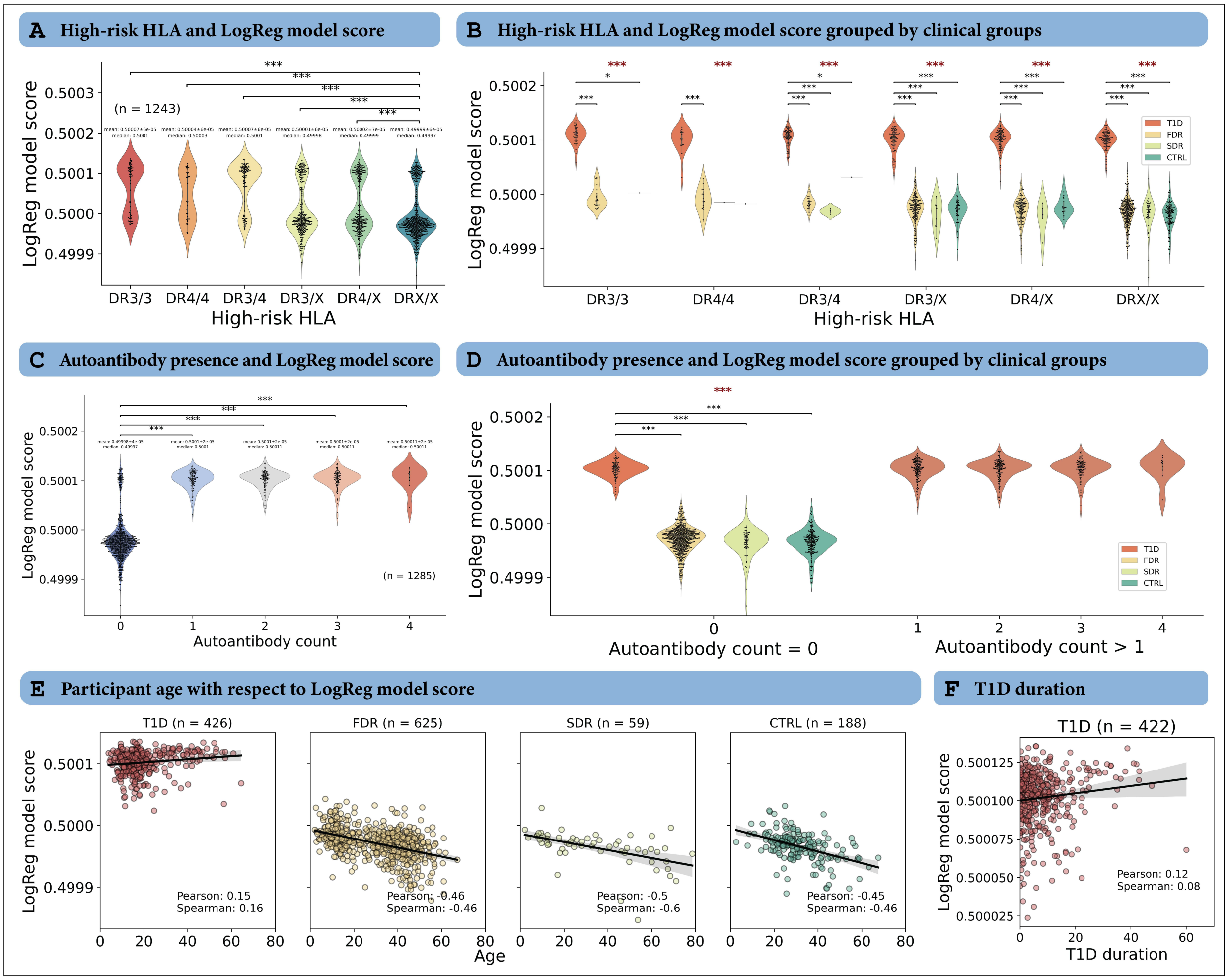

Fig. S19 | The logistic regression model exhibits signs of overfitting on cohort 1. (A) The logistic regression (LogReg) model scores in repertoires containing high-risk HLA alleles (DR3 or DR4) exhibited a significant difference compared to other HLA types (DRX/X). Notably, all combinations of high-risk HLA alleles formed two distinct clusters. The p-values in the figure were calculated with respect to the DRX/X HLA type. **(B)** The model score was plotted for each high-risk HLA type and grouped by the clinical groups. The model score was significantly high for T1D repertoires in all high-risk HLA combinations and did not show expected observation of T1D>FDR>SDR>CTRL. Here, pairwise testing was performed with respect to the T1D clinical group. **(C)** The LogReg model score also had significantly low values when no autoantibody were present. The p-values were calculated with respect to no presence of autoantibodies. **(D)** The LogReg model score was high for autoantibody count >0. However, in case of no presence of autoantibody, LogReg model score was significantly higher for the T1D repertoires and did not follow the expected trend of T1D>FDR>SDR>CTRL. **(E)** Interestingly, the LogReg model score plotted against the age of the individual, showed a positive correlation for T1D group and negative correlation for remaining clinical groups. **(F)** The T1D duration also showed a positive correlation with LogReg model score (pearson correlation: 0.27 and spearman correlation: 0.17). In the figure, the p-values for the multiple testing were performed using the Kruskal-Wallis test (denoted with red stars). Similarly, p-values for pairwise testing were calculated using two tailed Mann-Whitney U test. p-values were described as * for [0.01,0.05], ** for [0.001,0.01] and *** for <0.001 and no stars plotted for non-significant values. All p-values were adjusted for multiple testing using the Benjamini–Hochberg method. Relates to Fig. 4.

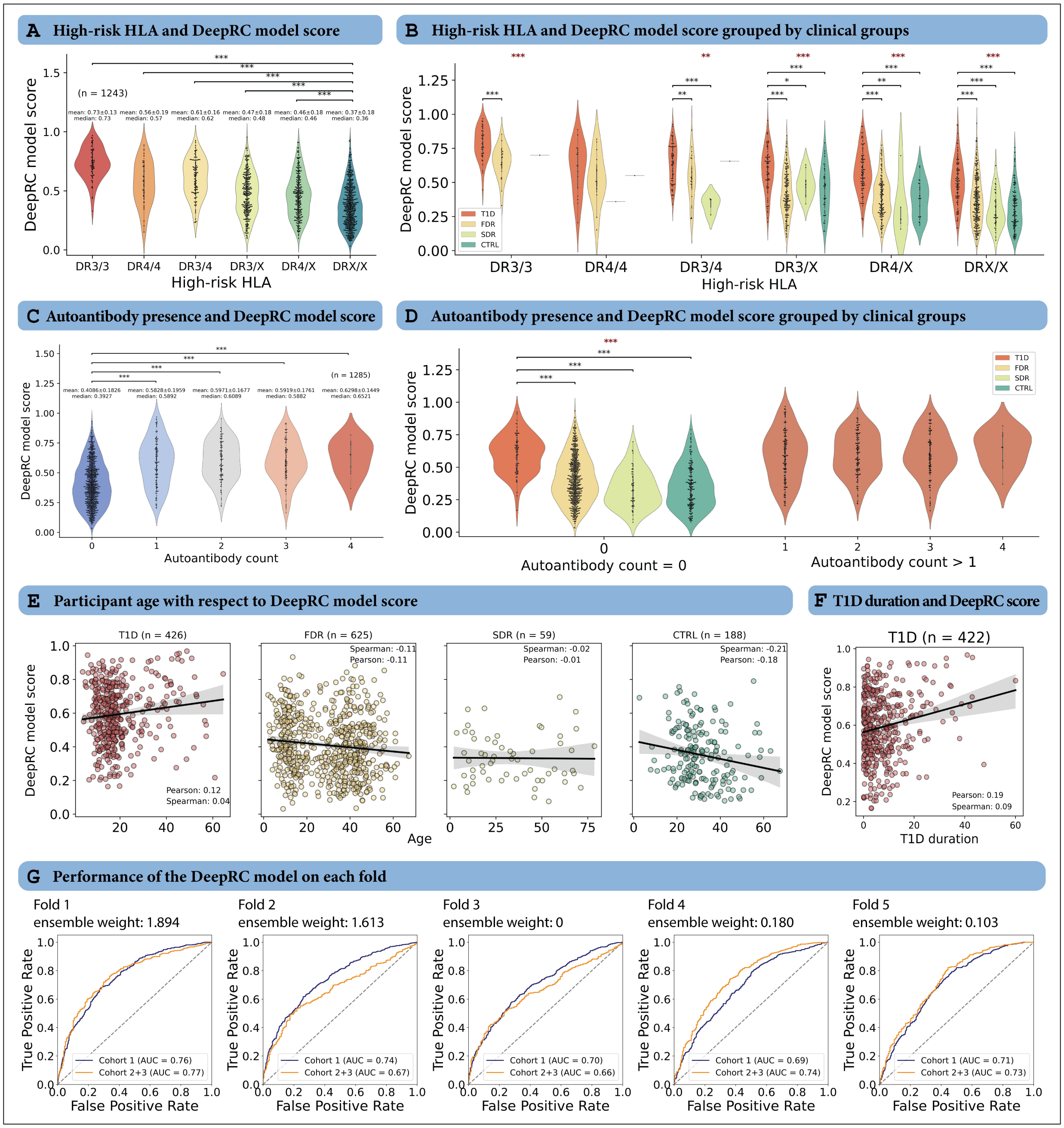

Fig. S20 | **Assessment of the DeepRC model performance demonstrates that TCR repertoires can be robust predictors of T1D risk. (A)** DeepRC model score in the repertoires with high-risk HLA alleles type (DR3 or DR4) was significantly higher compared to other HLA types (DRX/X). The p-values in the figure were calculated with respect to the DRX/X HLA type. **(B)** The DeepRC model score was plotted for each high-risk HLA type and grouped by the clinical groups. The DeepRC model score was significantly high in all clinical groups containing both alleles associated with high-risk (DR3 and DR4). However, it observed the expected trend T1D>FDR>CTRL in cases where at least one HLA allele was not associated with high-risk of T1D. Here, pairwise testing was performed with respect to the T1D clinical group. **(C)** The DeepRC model score also observed positive association with the number of autoantibodies present. The p-values were calculated with respect to no presence of autoantibodies. **(D)** DeepRC model score was high for all individuals when autoantibody count was >0. However, in case of no presence of autoantibody, DeepRC model score followed the expected trend where T1D>FDR>CTRL. **(E)** The DeepRC model score was plotted against age of the individual for different clinical groups (for cohort 1) and a negative correlation was observed for all clinical groups. **(F)** Interestingly, T1D duration observed a positive correlation with DeepRC model score (pearson correlation: 0.19 and spearman correlation: 0.09). **(G)** The final ensemble logistic regression (LR) model, built using the outputs

of each DeepRC model, assigned weights to each fold. These ensemble weights, along with the performance (AUROC curve) of the DeepRC model for each fold, were also presented. In the figure, the p-values for the multiple testing were performed using the Kruskal-Wallis test (denoted with red stars). Similarly, p-values for pairwise testing were calculated using two tailed Mann-Whitney U tests. p-values were described as * for [0.01,0.05], ** for [0.001,0.01] and *** for <0.001 and no stars plotted for non-significant values. All p-values were adjusted for multiple testing using the Benjamini–Hochberg method. Relates to **Fig. 4**.

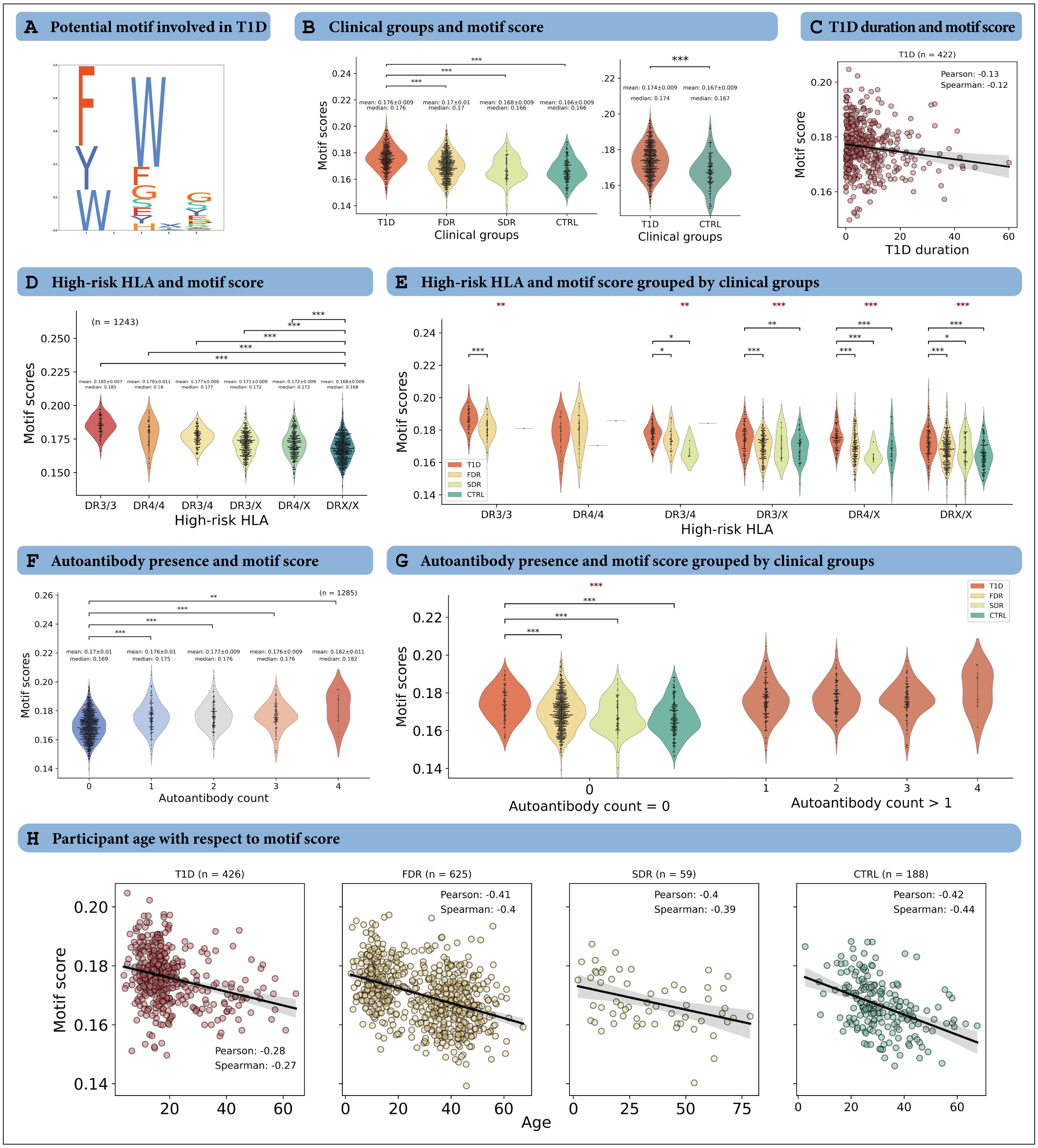

Fig. S21 | Assessment of the TCR repertoires-derived, interpretable version of the DeepRC model, referred to as DeepRC-motif, demonstrates a robust association with T1D status. The DeepRC-motif score represents the number of CDR3β sequences containing the DeepRC-motif normalized by total number of CDR3β sequences per repertoire, where higher DeepRC-motif score signifies higher risk of T1D. **(A)** The sequence logo representation of the DeepRC-motif. **(B)** DeepRC-motif score was significantly higher for T1D repertoires and observed the expected trend T1D>FDR>SDR>CTRL. **(C)** T1D duration observed a negative correlation with DeepRC-motif score (pearson correlation: -0.13 and spearman correlation: -0.12). **(D)** DeepRC-motif score in the repertoires with high-risk HLA alleles type (DR3 or DR4) was significantly higher compared to other HLA types (DRX/X). The p-values in the figure were calculated with respect to the DRX/X HLA type. **(E)** The DeepRC-motif score was plotted for each high-risk HLA type and grouped by the clinical groups. The DeepRC-motif score was significantly high in all clinical groups containing both alleles associated with high-risk (DR3 and DR4). However, it observed the expected trend T1D>FDR>CTRL in cases where at least one HLA allele was not associated with high-risk of T1D. Here, pairwise testing

was performed with respect to the T1D clinical group. **(F)** The DeepRC-motif score also observed positive association with the number of autoantibodies present. The p-values were calculated with respect to no presence of autoantibodies. **(G)** DeepRC-motif score was high for all individuals when autoantibody count was >0. However, in case of no presence of autoantibody, DeepRC-motif score followed the expected trend where T1D>FDR>CTRL. **(H)** The DeepRC-motif score was plotted against age of the individual for different clinical groups (for cohort 1) and a negative correlation was observed for all clinical groups. In the figure, the p-values for the multiple testing were performed using the Kruskal-Wallis test (denoted with red stars). Similarly, p-values for pairwise testing were calculated using two tailed Mann-Whitney U tests. p-values were described as * for [0.01,0.05], ** for [0.001,0.01] and *** for <0.001 and no stars plotted for non-significant values. All p-values were adjusted for multiple testing using the Benjamini–Hochberg method. Relates to **Fig. 4**.

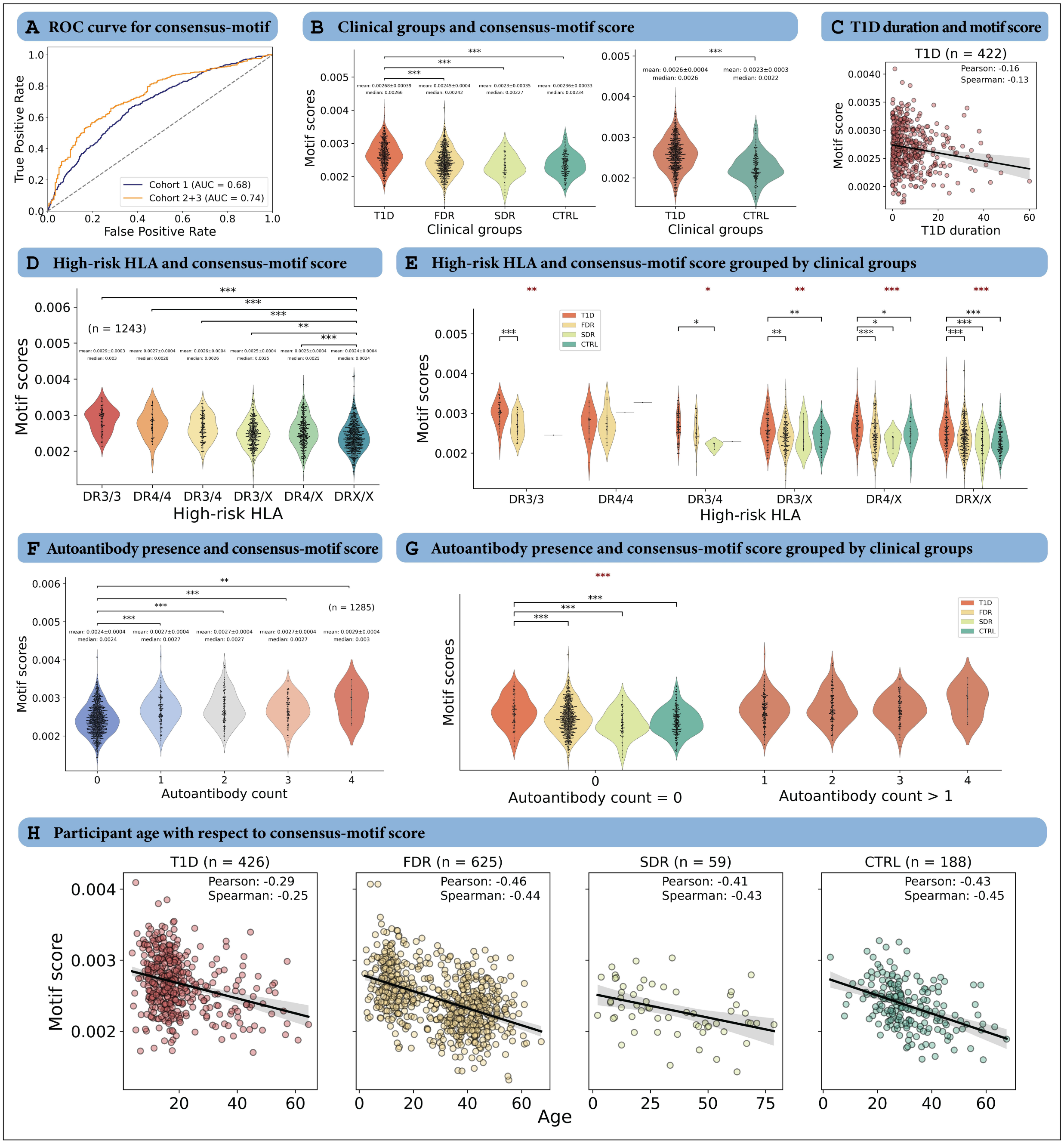

Fig. S22 | **The assessment of the consensus-motif, derived from the intersection of pHLA-motif and DeepRC-motif, shows positive association with the clinical groups, high-risk HLA alleles (DR3/DR4) and autoantibody presence. (A)** consensus-motif score was used for classification, where AUROC was 0.68 for cohort 1 and 0.74 for cohort 2+3. **(B)** The consensus-motif score was higher for T1D repertoires in cohort 1, following the expected trend of T1D > FDR > CTRL. A similar pattern was also observed in cohorts 2 and 3. The consensus-motif score represents the number of CDR3β sequences containing both pHLA-motif and DeepRC-motif normalized by total number of CDR3β sequences per repertoire, where higher consensus-motif score signifies higher risk of T1D. **(C)** The correlation between T1D duration and consensus-motif score shows a pearson correlation of -0.16 and spearman correlation of -0.13. **(D)** The consensus-motif score was significantly higher in the high-risk HLA type (DR3 or DR4) individuals than other HLA types (DRX/X). The p-values in the figure were calculated with respect to the DRX/X HLA type. **(E)** The consensus-motif score was plotted for each high-risk HLA type and grouped by the clinical groups. The consensus-motif score observed the expected trend T1D>FDR>CTRL in all cases irrespective of high-risk HLA allele type. Here, pairwise testing was performed with respect to the T1D clinical group. **(F)** The consensus-motif score also

showed positive association with the number of autoantibodies present. The p-values were calculated with respect to no presence of autoantibodies. **(G)** The consensus-motif score was high for autoantibody count >0. However, in case of no presence of autoantibody, consensus-motif score followed the expected trend where T1D>FDR>CTRL. **(H)** The consensus-motif score was plotted against age of the individual for different clinical groups (for cohort 1). Each clinical group showed negative correlation with the age of the individual. In the figure, the p-values for the multiple testing were performed using the Kruskal-Wallis test (denoted with red stars). Similarly, p-values for pairwise testing were calculated using two tailed Mann-Whitney U tests. p-values were described as * for [0.01,0.05], ** for [0.001,0.01] and *** for <0.001 and no stars plotted for non-significant values. All p-values were adjusted for multiple testing using the Benjamini–Hochberg method. Relates to **Fig. 3**, **Fig. 4**.

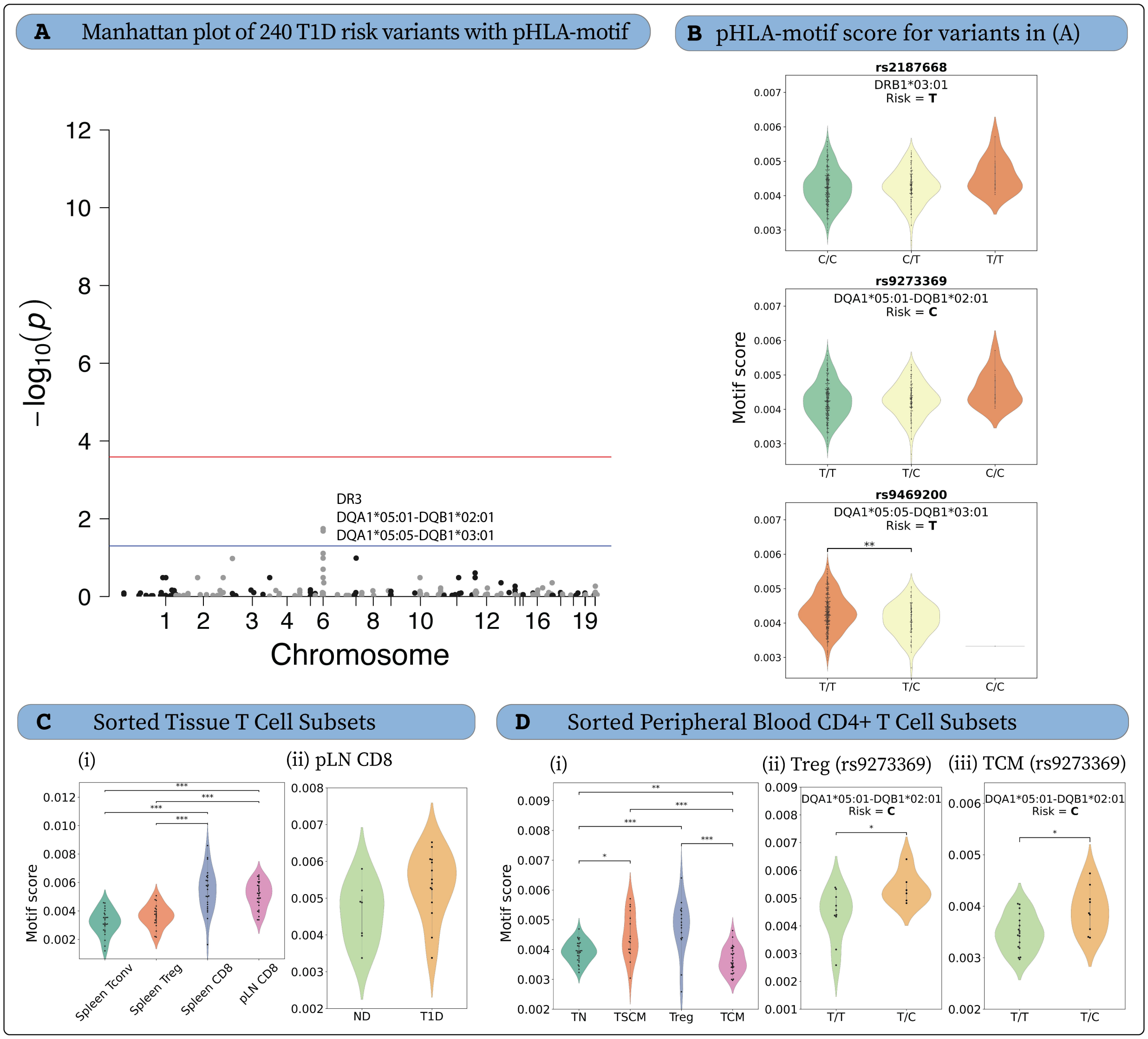

Fig. S23 | **Positively-associated HLA-motif (pHLA-motif) enriched in carriers of T1D risk genetics in bulk and sorted CD4^{+} Treg and TCM from peripheral blood as well as pancreatic lymph node CD8^{+} T cells.** (**A**) Manhattan plot of 240 T1D risk variants versus pHLA-motif score in bulk peripheral blood. Linear regression assuming additive genotypic effect with age, sex, T1D status, predicted probability of CMV infection, and 10 multidimensional scaling components as covariates. Benjamini-Hochberg false discovery rate threshold at p=2.58e-4 (red line) and a conventional threshold of p = 0.05 (blue line). **(B)** Violin plots of pHLA-motif score in European ancestry non-diabetic (ND) individuals, according to significantly associated variants in **(A)**. Tagged HLA genotype and risk allele annotated above each plot. (**C**) pHLA-motif score across sorted T cell subsets in spleen and pancreatic lymph node (pLN). **(i)** Mixed-effects analysis with Tukey's multiple comparisons test. (**ii**) pLN CD8^{+} T cell pHLA-motif score according to T1D status. (**D**) pHLA-motif score in sorted peripheral blood CD4^{+} T cell subsets. **(i)** Mixed-effects analysis with Tukey's multiple comparisons test. (**ii**) Treg and (**iii**) CD4^{+} TCM pHLA-motif score according to DQA1*05:01-DQB1*02:01 tag SNP. Linear regression with age, sex, and T1D status as covariates. In all violin plots, p-values for pairwise testing were calculated using two tailed Mann-Whitney U tests and p-values were adjusted between different clinical groups using the Benjamini–Hochberg method. p-values were described as * for [0.01,0.05], ** for [0.001,0.01] and *** for <0.001 and no stars plotted for non-significant values. Relates to **Fig. 5**.

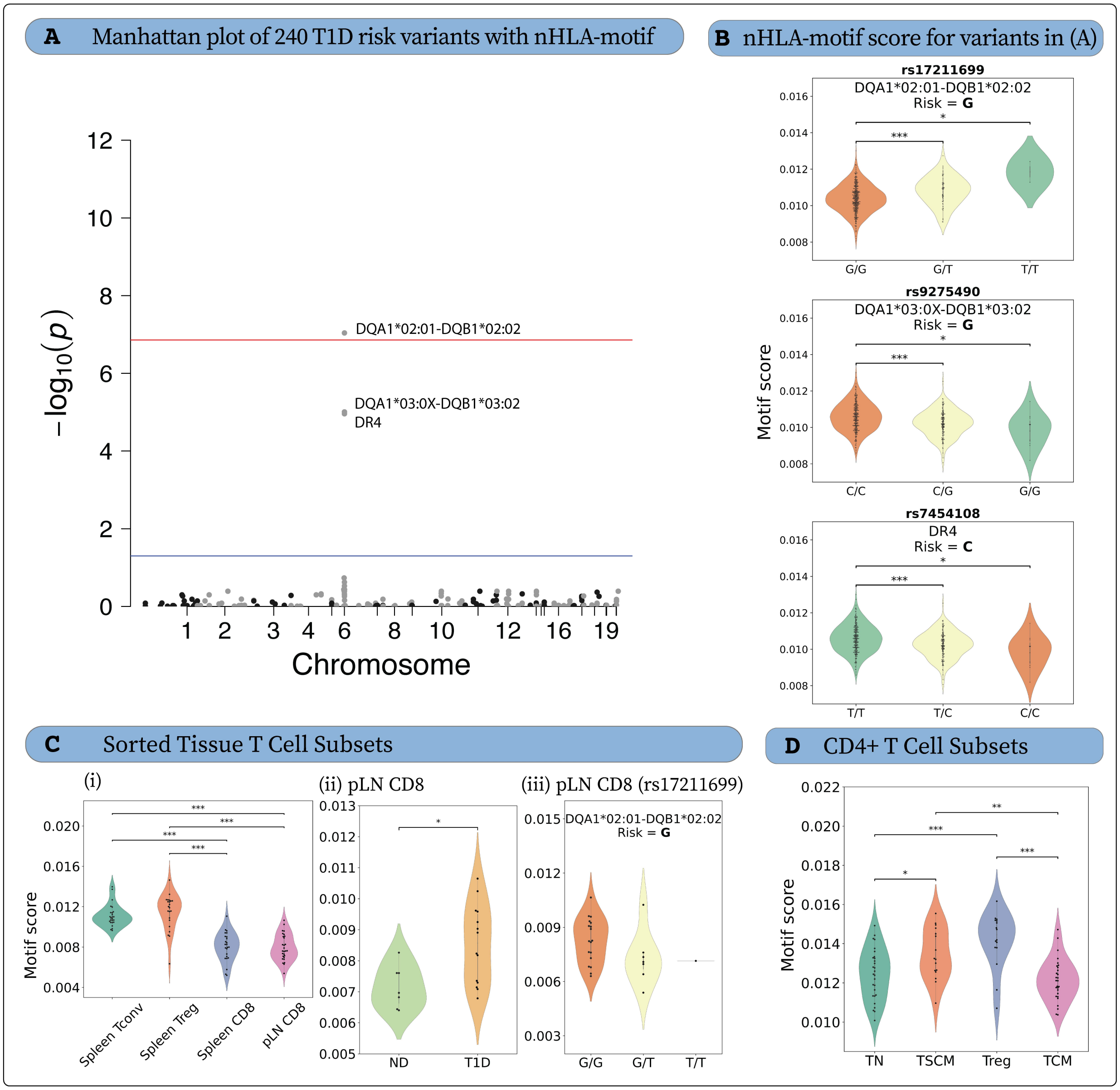

Fig. S24 | **Negatively-associated HLA-motif (nHLA-motif) enriched in carriers of T1D protective genetics in bulk peripheral blood and carriers of T1D risk genetics in pancreatic lymph node CD8⁺ T cells.** (**A**) Manhattan plot of 240 T1D risk variants versus nHLA-motif score in bulk peripheral blood. Linear regression assuming additive genotypic effect with age, sex, T1D status, predicted probability of CMV infection, and 10 multidimensional scaling components as covariates. Benjamini-Hochberg false discovery rate threshold at p=1.38e-7 (red line) and a conventional threshold of p = 0.05 (blue line). **(B)** Violin plots of nHLA-motif score in European ancestry non-diabetic (ND) individuals, according to significantly associated variants in **(A)**. Tagged HLA genotype and risk allele annotated above each plot. (**C**) nHLA-motif score across sorted T cell subsets in spleen and pancreatic lymph node (pLN). **(i)** Mixed-effects analysis with Tukey's multiple comparisons test. (**ii**) pLN $CD8^+$ T cell nHLA-motif score according to T1D status. (**iii**) pLN $CD8^+$ T cell nHLA-motif score according to DQA1*02:01-DQB1*02:02 tag SNP. **(D)** nHLA-Motif score across sorted CD4+ T cell subsets, where mixed-effects analysis was done with Tukey's multiple comparisons test. Linear regression with age, sex, diabetes status, and 10 multidimensional scaling components as covariates. In all violin plots, p-values for pairwise testing were calculated using two tailed Mann-Whitney U tests and p-values were adjusted between different clinical groups using the Benjamini–Hochberg method. p-values were described as * for [0.01,0.05], ** for [0.001,0.01] and *** for <0.001 and no stars plotted for non-significant values. Relates to **Fig. 5**.

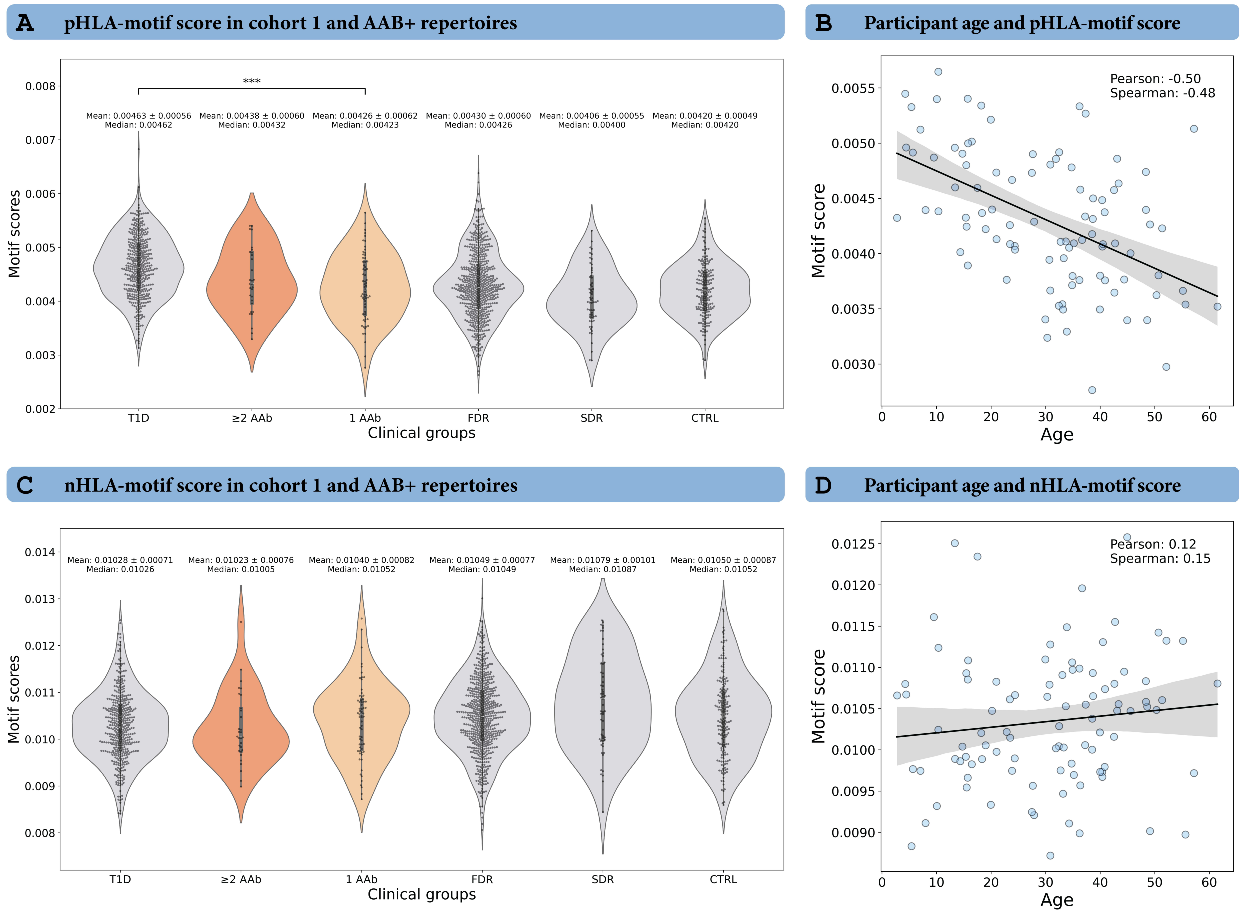

Fig. S25 | HLA-associated TCR motif scores in autoantibody-positive, non-diabetic repertoires (AAb+) are intermediate between controls and T1D groups. AAb+ individuals (n = 95) were stratified by autoantibody count: single autoantibody ("1 AAb", n = 65) and multiple autoantibodies ("≥2 AAb", n = 30). **(A)** In cohort 1, both the "1 AAb" and "≥2 AAb" subgroups of AAb+ repertoires exhibited higher pHLA-motif scores than controls, yet these scores remained lower than those in the T1D group. **(B)** pHLA-motif score showed a strong negative correlation (r = –0.5) with participant age in AAb+ group. In contrast, **(C)** Comparison of nHLA-motif score for the "1 AAb" and "≥2 AAb" subgroups of AAb+ repertoires against all other clinical groups in cohort 1. The protective nHLA-motif score in AAb+ repertoires was lower compared to controls but higher than T1D, and **(D)** it showed a weak positive correlation (r = 0.12) with age in the AAb+ group. In all violin plots, statistical significance was evaluated by conducting pairwise comparisons of the '1 AAb' and '≥2 AAb' subgroups against the remaining clinical groups. p-values for pairwise testing were calculated using two tailed Mann-Whitney U tests and adjusted using the Benjamini–Hochberg method. p-values were described as * for [0.01,0.05], ** for [0.001,0.01] and *** for <0.001 and no stars plotted for non-significant values. Relates to Fig. 5.

REFERENCES

1. S. Ahmed, K. Cerosaletti, E. James, S. A. Long, S. Mannering, C. Speake, M. Nakayama, T. Tree, B. O. Roep, K. C. Herold, T. M. Brusko, Standardizing T-cell biomarkers in type 1 diabetes: Challenges and recent advances. *Diabetes* **68**, 1366–1379 (2019).

2. M. Nakayama, A. W. Michels, Using the T cell receptor as a biomarker in type 1 diabetes. *Front. Immunol.* **12**, 777788 (2021).

3. W. S. DeWitt 3rd, A. Smith, G. Schoch, J. A. Hansen, F. A. Matsen 4th, P. Bradley, Human T cell receptor occurrence patterns encode immune history, genetic background, and receptor specificity. *Elife* **7**, e38358 (2018).

4. M. R. Ortega, M. V. Pogorelyy, A. A. Minervina, P. G. Thomas, A. M. Walczak, T. Mora, Learning predictive signatures of HLA type from T-cell repertoires. bioRxiv 577228 [Preprint] (2024). https://doi.org/10.1101/2024.01.25.577228.

5. H. J. Zahid, R. Taniguchi, P. Ebert, I.-T. Chow, C. Gooley, J. Lv, L. Pisani, M. Rusnak, R. Elyanow, H. Takamatsu, W. Zhou, J. Greissl, H. Robins, J. M. Carlson, Large-scale statistical mapping of T-cell receptor β sequences to human leukocyte antigens. bioRxiv 587617 [Preprint] (2024). https://doi.org/10.1101/2024.04.01.587617.

6. Y. Nagafuchi, M. Ota, H. Hatano, M. Inoue, S. Kobayashi, M. Okubo, Y. Sugimori, M. Nakano, S. Yamada, R. Yoshida, Y. Tsuchida, Y. Iwasaki, H. Shoda, Y. Okada, K. Yamamoto, K. Ishigaki, T. Okamura, K. Fujio, Control of naive and effector CD4 T cell receptor repertoires by rheumatoid-arthritis-risk HLA alleles. *J. Autoimmun.* **133**, 102907 (2022).

7. K. Ishigaki, K. A. Lagattuta, Y. Luo, E. A. James, J. H. Buckner, S. Raychaudhuri, HLA autoimmune risk alleles restrict the hypervariable region of T cell receptors. *Nat. Genet.* **54**, 393–402 (2022).

8. H. ElAbd, M. Pesesky, G. Innocenti, B. K. Chung, A. K. H. Mahdy, V. Kriukova, L. Kulsvehagen, D. Strobbe, C. Stühler, G. Mayr, D. H. May, M. Prinzensteiner, T. A. Steiert, F. Tran, M. V. Hadjihannas, R. Günther, E. Rosati, S. Mucha, W. Lieb, M. Ziemann, A. Dempfle,

F. Braun, T. Folseraas, J. R. Hov, E. Melum, P. Bacher, M. Sterneck, T. J. Weismüller, H. Lenzen, B. Bokemeyer, B. Howie, H. S. Robins, C. Röcken, S. Schreiber, N. Khanna, A.-K. Pröbstel, C. Schramm, T. Vogl, T. H. Karlsen, A. Franke, T and B cell responses against Epstein-Barr virus in primary sclerosing cholangitis. *Nat. Med.*, **31**, 2306–2316 (2025).

9. J. A. Noble, A. M. Valdes, M. Cook, W. Klitz, G. Thomson, H. A. Erlich, The role of HLA class II genes in insulin-dependent diabetes mellitus: Molecular analysis of 180 Caucasian, multiplex families. *Am. J. Hum. Genet.* **59**, 1134–1148 (1996).

10. A. P. Lambert, K. M. Gillespie, G. Thomson, H. J. Cordell, J. A. Todd, E. A. M. Gale, P. J. Bingley, Absolute risk of childhood-onset type 1 diabetes defined by human leukocyte antigen class II genotype: A population-based study in the United Kingdom. *J. Clin. Endocrinol. Metab.* **89**, 4037–4043 (2004).

11. J. A. Noble, A. M. Valdes, Genetics of the HLA region in the prediction of type 1 diabetes. *Curr. Diab. Rep.* **11**, 533–542 (2011).

12. J. P. Krischer, X. Liu, Å. Lernmark, W. A. Hagopian, M. J. Rewers, J.-X. She, J. Toppari, A.-G. Ziegler, B. Akolkar, TEDDY Study Group, Predictors of the initiation of islet autoimmunity and progression to multiple autoantibodies and clinical diabetes: The TEDDY Study. *Diabetes Care* **45**, 2271–2281 (2022).

13. J. Greissl, M. Pesesky, S. C. Dalai, A. W. Rebman, M. J. Soloski, E. J. Horn, J. N. Dines, R. M. Gittelman, T. M. Snyder, R. O. Emerson, E. Meeds, T. Manley, I. M. Kaplan, L. Baldo, J. M. Carlson, H. S. Robins, J. N. Aucott, Immunosequencing of the T-cell receptor repertoire reveals signatures specific for diagnosis and characterization of early Lyme disease. medRxiv 21261353 [Preprint] (2021). https://doi.org/10.1101/2021.07.30.21261353.

14. J. J. Ross, C. H. Wasserfall, R. Bacher, D. J. Perry, K. McGrail, A. L. Posgai, X. Dong, A. Muir, X. Li, M. Campbell-Thompson, T. M. Brusko, D. A. Schatz, M. J. Haller, M. A. Atkinson, Exocrine pancreatic enzymes are a serological biomarker for type 1 diabetes staging and pancreas size. *Diabetes* **70**, 944–954 (2021).

15. S. J. Hanna, R. H. Bonami, B. Corrie, M. Westley, A. L. Posgai, E. T. Luning Prak, F. Breden, A. W. Michels, T. M. Brusko, Type 1 Diabetes AIRR Consortium, The Type 1 Diabetes T Cell Receptor and B Cell Receptor Repository in the AIRR Data Commons: A practical guide for access, use and contributions through the Type 1 Diabetes AIRR Consortium. *Diabetologia* **68**, 186–202 (2025).

16. V. Greiff, G. Yaari, L. Cowell, Mining adaptive immune receptor repertoires for biological and clinical information using machine learning. *Curr. Opin. Syst. Biol.* **24**, 109–119 (2020).

17. R. A. Arnaout, E. T. L. Prak, N. Schwab, F. Rubelt, Adaptive immune receptor repertoire community, The future of blood testing is the immunome. *Front. Immunol.* **12**, 626793 (2021).

18. V. Mhanna, H. Bashour, K. Lê Quý, P. Barennes, P. Rawat, V. Greiff, E. Mariotti-Ferrandiz, Adaptive immune receptor repertoire analysis. *Nat. Rev. Methods Primers* **4**, 6 (2024).

19. X. Liu, W. Zhang, M. Zhao, L. Fu, L. Liu, J. Wu, S. Luo, L. Wang, Z. Wang, L. Lin, Y. Liu, S. Wang, Y. Yang, L. Luo, J. Jiang, X. Wang, Y. Tan, T. Li, B. Zhu, Y. Zhao, X. Gao, Z. Wan, C. Huang, M. Fang, Q. Li, H. Peng, X. Liao, J. Chen, F. Li, G. Ling, H. Zhao, H. Luo, Z. Xiang, J. Liao, Y. Liu, H. Yin, H. Long, H. Wu, H. Yang, J. Wang, Q. Lu, T cell receptor β repertoires as novel diagnostic markers for systemic lupus erythematosus and rheumatoid arthritis. *Ann. Rheum. Dis.* **78**, 1070–1078 (2019).

20. R. O. Emerson, W. S. DeWitt, M. Vignali, J. Gravley, J. K. Hu, E. J. Osborne, C. Desmarais, M. Klinger, C. S. Carlson, J. A. Hansen, M. Rieder, H. S. Robins, Immunosequencing identifies signatures of cytomegalovirus exposure history and HLA-mediated effects on the T cell repertoire. *Nat. Genet.* **49**, 659–665 (2017).

21. T. M. Snyder, R. M. Gittelman, M. Klinger, D. H. May, E. J. Osborne, R. Taniguchi, H. J. Zahid, I. M. Kaplan, J. N. Dines, M. T. Noakes, R. Pandya, X. Chen, S. Elasady, E. Svejnoha, P. Ebert, M. W. Pesesky, P. De Almeida, H. O'Donnell, Q. DeGottardi, G. Keitany, J. Lu, A. Vong, R. Elyanow, P. Fields, J. Greissl, L. Baldo, S. Semprini, C. Cerchione, F. Nicolini, M. Mazza, O. M. Delmonte, K. Dobbs, R. Laguna-Goya, G. Carreño-Tarragona, S. Barrio, L. Imberti, A. Sottini, E. Quiros-Roldan, C. Rossi, A. Biondi, L. R. Bettini, M. D'Angio, P. Bonfanti, M. F.

Tompkins, C. Alba, C. Dalgard, V. Sambri, G. Martinelli, J. D. Goldman, J. R. Heath, H. C. Su, L. D. Notarangelo, E. Paz-Artal, J. Martinez-Lopez, J. M. Carlson, H. S. Robins, Magnitude and dynamics of the T-cell response to SARS-CoV-2 infection at both individual and population levels. medRxiv 20165647 [Preprint] (2020), doi: https://doi.org/10.1101/2020.07.31.20165647.

22. X. Yu, M. Pan, J. Ye, C. A. Hathaway, S. S. Tworoger, J. Lea, B. Li, Quantifiable TCR repertoire changes in prediagnostic blood specimens among patients with high-grade ovarian cancer. *Cell Rep. Med.* **5**, 101612 (2024).

23. T. J. O'Donnell, C. Kanduri, G. Isacchini, J. P. Limenitakis, R. A. Brachman, R. A. Alvarez, I. H. Haff, G. K. Sandve, V. Greiff, Reading the repertoire: Progress in adaptive immune receptor analysis using machine learning. *Cell Syst.* **15**, 1168–1189 (2024).

24. N. D. Chu, H. S. Bi, R. O. Emerson, A. M. Sherwood, M. E. Birnbaum, H. S. Robins, E. J. Alm, Longitudinal immunosequencing in healthy people reveals persistent T cell receptors rich in highly public receptors. *BMC Immunol.* **20**, 19 (2019).

25. C. R. Weber, T. Rubio, L. Wang, W. Zhang, P. A. Robert, R. Akbar, I. Snapkov, J. Wu, M. L. Kuijjer, S. Tarazona, A. Conesa, G. K. Sandve, X. Liu, S. T. Reddy, V. Greiff, Reference-based comparison of adaptive immune receptor repertoires. *Cell Rep. Methods* **2**, 100269 (2022).

26. A. Christophersen, M. Ráki, E. Bergseng, K. E. Lundin, J. Jahnsen, L. M. Sollid, S.-W. Qiao, Tetramer-visualized gluten-specific CD4+ T cells in blood as a potential diagnostic marker for coeliac disease without oral gluten challenge. *United European Gastroenterol. J.* **2**, 268–278 (2014).

27. A. Christophersen, E. G. Lund, O. Snir, E. Solà, C. Kanduri, S. Dahal-Koirala, S. Zühlke, Ø. Molberg, P. J. Utz, M. Rohani-Pichavant, J. F. Simard, C. L. Dekker, K. E. A. Lundin, L. M. Sollid, M. M. Davis, Distinct phenotype of $CD4^{+}$ T cells driving celiac disease identified in multiple autoimmune conditions. *Nat. Med.* **25**, 734–737 (2019).

28. S. Dahal-Koirala, G. Balaban, R. S. Neumann, L. Scheffer, K. E. A. Lundin, V. Greiff, L. M. Sollid, S.-W. Qiao, G. K. Sandve, TCRpower: Quantifying the detection power of T-cell receptor

sequencing with a novel computational pipeline calibrated by spike-in sequences. *Brief. Bioinform.* **23**, bbab566 (2022).

29. N. De Neuter, E. Bartholomeus, G. Elias, N. Keersmaekers, A. Suls, H. Jansens, E. Smits, N. Hens, P. Beutels, P. Van Damme, G. Mortier, V. Van Tendeloo, K. Laukens, P. Meysman, B. Ogunjimi, Memory $CD4^{+}$ T cell receptor repertoire data mining as a tool for identifying cytomegalovirus serostatus. *Genes Immun.* **20**, 255–260 (2019).

30. J. Ostmeyer, S. Christley, I. T. Toby, L. G. Cowell, Biophysicochemical motifs in T-cell receptor sequences distinguish repertoires from tumor-infiltrating Lymphocyte and adjacent healthy tissue. *Cancer Res.* **79**, 1671–1680 (2019).

31. P. Schmidt-Barbo, G. Kalweit, M. Naouar, L. Paschold, E. Willscher, C. Schultheiß, B. Märkl, S. Dirnhofer, A. Tzankov, M. Binder, M. Kalweit, Detection of disease-specific signatures in B cell repertoires of lymphomas using machine learning. *PLOS Comput. Biol.* **20**, e1011570 (2024).

32. J.-W. Sidhom, G. Oliveira, P. Ross-MacDonald, M. Wind-Rotolo, C. J. Wu, D. M. Pardoll, A. S. Baras, Deep learning reveals predictive sequence concepts within immune repertoires to immunotherapy. *Sci. Adv.* **8**, eabq5089 (2022).

33. T. Schneider-Hohendorf, C. Wünsch, S. Falk, C. Raposo, F. Rubelt, H. Mirebrahim, H. Asgharian, U. Schlecht, D. Mattox, W. Zhou, E. Dawin, M. Pawlitzki, S. Lauks, S. Jarius, B. Wildemann, J. Havla, T. Kümpfel, M.-C. Schrot, M. Ringelstein, M. Kraemer, C. Schwake, T. Schmitter, I. Ayzenberg, K. Fischer, S. G. Meuth, O. Aktas, M. W. Hümmert, J. R. Kretschmer, C. Trebst, I. Kleffner, J. Massey, P. A. Muraro, H. Chen-Harris, C. C. Gross, L. Klotz, H. Wiendl, N. Schwab, Broader anti-EBV TCR repertoire in multiple sclerosis: Disease specificity and treatment modulation. *Brain* **148**, 933–940 (2025).

34. Y. Nagafuchi, M. Nakano, K. A. Lagattuta, M. Ota, H. Hatano, H. Takahashi, T. Itamiya, H. Inokuchi, S. Raychaudhuri, T. Okamura, K. Fujio, K. Ishigaki, T cell plasticity in systemic lupus erythematosus revealed by large-scale T cell receptor repertoire and transcriptome studies. medRxiv 24319648 [Preprint] (2025). https://doi.org/10.1101/2025.01.06.24319648.

35. E. A. Komech, M. V. Pogorelyy, E. S. Egorov, O. V. Britanova, D. V. Rebrikov, A. G. Bochkova, E. I. Shmidt, N. A. Shostak, M. Shugay, S. Lukyanov, I. Z. Mamedov, Y. B. Lebedev, D. M. Chudakov, I. V. Zvyagin, $CD8^+$ T cells with characteristic T cell receptor beta motif are detected in blood and expanded in synovial fluid of ankylosing spondylitis patients. *Rheumatology* **57**, 1097–1104 (2018).

36. M. Widrich, B. Schäfl, M. Pavlović, H. Ramsauer, L. Gruber, M. Holzleitner, J. Brandstetter, G. K. Sandve, V. Greiff, S. Hochreiter, G. Klambauer, Modern Hopfield networks and attention for immune repertoire classification. bioRxiv 038158 [Preprint] (2020). https://doi.org/10.1101/2020.04.12.038158.

37. J.-W. Sidhom, H. Benjamin Larman, D. M. Pardoll, A. S. Baras, DeepTCR is a deep learning framework for revealing sequence concepts within T-cell repertoires. *Nat. Commun.* **12**, 1605.

38. A. Slabodkin, L. M. Sollid, G. K. Sandve, P. A. Robert, V. Greiff, Weakly supervised identification and generation of adaptive immune receptor sequences associated with immune disease status. bioRxiv 558823 [Preprint] (2023). https://doi.org/10.1101/2023.09.24.558823.

39. S. Culina, A. I. Lalanne, G. Afonso, K. Cerosaletti, S. Pinto, G. Sebastiani, K. Kuranda, L. Nigi, A. Eugster, T. Østerbye, A. Maugein, J. E. McLaren, K. Ladell, E. Larger, J.-P. Beressi, A. Lissina, V. Appay, H. W. Davidson, S. Buus, D. A. Price, M. Kuhn, E. Bonifacio, M. Battaglia, S. Caillat-Zucman, F. Dotta, R. Scharfmann, B. Kyewski, R. Mallone, ImMaDiab Study Group, Islet-reactive $CD8^+$ T cell frequencies in the pancreas, but not in blood, distinguish type 1 diabetic patients from healthy donors. *Sci Immunol* **3**, eaao4013 (2018).

40. A. M. Mitchell, E. E. Baschal, K. A. McDaniel, K. M. Simmons, L. Pyle, K. Waugh, A. K. Steck, L. Yu, P. A. Gottlieb, M. J. Rewers, M. Nakayama, A. W. Michels, Temporal development of T cell receptor repertoires during childhood in health and disease. *JCI Insight* **7**, e161885 (2022).

41. I. Gomez-Tourino, Y. Kamra, R. Baptista, A. Lorenc, M. Peakman, T cell receptor β-chains display abnormal shortening and repertoire sharing in type 1 diabetes. *Nat. Commun.* **8**, 1792 (2017).

42. H. R. Seay, E. Yusko, S. J. Rothweiler, L. Zhang, A. L. Posgai, M. Campbell-Thompson, M. Vignali, R. O. Emerson, J. S. Kaddis, D. Ko, M. Nakayama, M. J. Smith, J. C. Cambier, A. Pugliese, M. A. Atkinson, H. S. Robins, T. M. Brusko, Tissue distribution and clonal diversity of the T and B cell repertoire in type 1 diabetes. *JCI Insight* **1**, e88242 (2016).

43. A. Eugster, A. Lindner, M. Catani, A.-K. Heninger, A. Dahl, S. Klemroth, D. Kühn, S. Dietz, M. Bickle, A.-G. Ziegler, E. Bonifacio, High diversity in the TCR repertoire of GAD65 autoantigen-specific human $CD4^+$ T cells. *J. Immunol.* **194**, 2531–2538 (2015).

44. K. Cerosaletti, F. Barahmand-Pour-Whitman, J. Yang, H. A. DeBerg, M. J. Dufort, S. A. Murray, E. Israelsson, C. Speake, V. H. Gersuk, J. A. Eddy, H. Reijonen, C. J. Greenbaum, W. W. Kwok, E. Wambre, M. Prlic, R. Gottardo, G. T. Nepom, P. S. Linsley, Single-cell RNA sequencing reveals expanded clones of islet antigen-reactive $CD4^+$ T cells in peripheral blood of subjects with type 1 diabetes. *J. Immunol.* **199**, 323–335 (2017).

45. D. J. Perry, M. R. Shapiro, S. W. Chamberlain, I. Kusmartseva, S. Chamala, L. Balzano-Nogueira, M. Yang, J. O. Brant, M. Brusko, M. D. Williams, K. M. McGrail, J. McNichols, L. D. Peters, A. L. Posgai, J. S. Kaddis, C. E. Mathews, C. H. Wasserfall, B.-J. M. Webb-Robertson, M. Campbell-Thompson, D. Schatz, C. Evans-Molina, A. Pugliese, P. Concannon, M. S. Anderson, M. S. German, C. E. Chamberlain, M. A. Atkinson, T. M. Brusko, A genomic data archive from the Network for Pancreatic Organ Donors with Diabetes. *Sci. Data* **10**, 323 (2023).

46. M. D. Williams, R. Bacher, D. J. Perry, C. R. Grace, K. M. McGrail, A. L. Posgai, A. Muir, S. Chamala, M. J. Haller, D. A. Schatz, T. M. Brusko, M. A. Atkinson, C. H. Wasserfall, Genetic Composition and autoantibody titers model the probability of detecting C-peptide following type 1 diabetes diagnosis. *Diabetes* **70**, 932–943 (2021).

47. X. Jia, B. Han, S. Onengut-Gumuscu, W.-M. Chen, P. J. Concannon, S. S. Rich, S. Raychaudhuri, P. I. W. de Bakker, Imputing amino acid polymorphisms in human leukocyte antigens. *PLOS ONE* **8**, e64683 (2013).

48. V. Greiff, P. Bhat, S. C. Cook, U. Menzel, W. Kang, S. T. Reddy, A bioinformatic framework for immune repertoire diversity profiling enables detection of immunological status. *Genome Med.* **7**, 49 (2015).

49. J. Chiffelle, R. Genolet, M. A. Perez, G. Coukos, V. Zoete, A. Harari, T-cell repertoire analysis and metrics of diversity and clonality. *Curr. Opin. Biotechnol.* **65**, 284–295 (2020).

50. M. Vujović, P. Marcatili, B. Chain, J. Kaplinsky, T. L. Andresen, Signatures of T cell immunity revealed using sequence similarity with TCRDivER algorithm. *Commun. Biol.* **6**, 357 (2023).

51. E. Miho, A. Yermanos, C. R. Weber, C. T. Berger, S. T. Reddy, V. Greiff, Computational strategies for dissecting the high-dimensional complexity of adaptive immune repertoires. *Front. Immunol.* **9**, 224 (2018).

52. O. V. Britanova, E. V. Putintseva, M. Shugay, E. M. Merzlyak, M. A. Turchaninova, D. B. Staroverov, D. A. Bolotin, S. Lukyanov, E. A. Bogdanova, I. Z. Mamedov, Y. B. Lebedev, D. M. Chudakov, Age-related decrease in TCR repertoire diversity measured with deep and normalized sequence profiling. *J. Immunol.* **192**, 2689–2698 (2014).

53. O. V. Britanova, K. R. Lupyr, D. B. Staroverov, I. A. Shagina, A. A. Aleksandrov, Y. Y. Ustyugov, D. V. Somov, A. Klimenko, N. A. Shostak, I. V. Zvyagin, A. V. Stepanov, E. M. Merzlyak, A. N. Davydov, M. Izraelson, E. S. Egorov, E. A. Bogdanova, A. K. Vladimirova, P. A. Iakovlev, D. A. Fedorenko, R. A. Ivanov, V. I. Skvortsova, S. Lukyanov, D. M. Chudakov, Targeted depletion of TRBV9^{+} T cells as immunotherapy in a patient with ankylosing spondylitis. *Nat. Med.* **29**, 2731–2736 (2023).

54. V. Greiff, U. Menzel, U. Haessler, S. C. Cook, S. Friedensohn, T. A. Khan, M. Pogson, I. Hellmann, S. T. Reddy, Quantitative assessment of the robustness of next-generation sequencing of antibody variable gene repertoires from immunized mice. *BMC Immunol.* **15**, 40 (2014).

55. N. J. Gotelli, R. K. Colwell, Quantifying biodiversity: Procedures and pitfalls in the measurement and comparison of species richness. *Ecol. Lett.* **4**, 379–391 (2001).

56. M. Estorninho, V. B. Gibson, D. Kronenberg-Versteeg, Y.-F. Liu, C. Ni, K. Cerosaletti, M. Peakman, A novel approach to tracking antigen-experienced CD4 T cells into functional compartments via tandem deep and shallow TCR clonotyping. *J. Immunol.* **191**, 5430–5440 (2013).

57. C. E. Shannon, A mathematical theory of communication. *Bell Syst. Tech. J.* **27**, 379–423 (1948).

58. R. J. M. Bashford-Rogers, A. L. Palser, B. J. Huntly, R. Rance, G. S. Vassiliou, G. A. Follows, P. Kellam, Network properties derived from deep sequencing of human B-cell receptor repertoires delineate B-cell populations. *Genome Res.* **23**, 1874–1884 (2013).

59. E. H. Simpson, Measurement of Diversity. *Nature* **163**, 688 (1949).

60. W. H. Berger, F. L. Parker, Diversity of planktonic foraminifera in deep-sea sediments. *Science* **168**, 1345–1347 (1970).

61. V. Greiff, C. R. Weber, J. Palme, U. Bodenhofer, E. Miho, U. Menzel, S. T. Reddy, Learning the high-dimensional immunogenomic features that predict public and private antibody repertoires. *J. Immunol.* **199**, 2985–2997 (2017).

62. R. Amoriello, M. Chernigovskaya, V. Greiff, A. Carnasciali, L. Massacesi, A. Barilaro, A. M. Repice, T. Biagioli, A. Aldinucci, P. A. Muraro, D. A. Laplaud, A. Lossius, C. Ballerini, TCR repertoire diversity in multiple sclerosis: High-dimensional bioinformatics analysis of sequences from brain, cerebrospinal fluid and peripheral blood. *EBioMedicine* **68**, 103429 (2021).

63. N. Tickotsky, T. Sagiv, J. Prilusky, E. Shifrut, N. Friedman, McPAS-TCR: A manually curated catalogue of pathology-associated T cell receptor sequences. *Bioinformatics* **33**, 2924–2929 (2017).

64. D. V. Bagaev, R. M. A. Vroomans, J. Samir, U. Stervbo, C. Rius, G. Dolton, A. Greenshields-Watson, M. Attaf, E. S. Egorov, I. V. Zvyagin, N. Babel, D. K. Cole, A. J. Godkin, A. K. Sewell, C. Kesmir, D. M. Chudakov, F. Luciani, M. Shugay, VDJdb in 2019: Database extension, new

analysis infrastructure and a T-cell receptor motif compendium. *Nucleic Acids Res.* **48**, D1057–D1062 (2020).

65. A. Cohn, A. M. Sofia, S. S. Kupfer, Type 1 diabetes and celiac disease: Clinical overlap and new insights into disease pathogenesis. *Curr. Diab. Rep.* **14**, 517 (2014).

66. U. Volta, F. Tovoli, G. Caio, Clinical and immunological features of celiac disease in patients with type 1 diabetes mellitus. *Expert Rev. Gastroenterol. Hepatol.* **5**, 479–487 (2011).

67. A. M. Mitchell, E. E. Baschal, K. A. McDaniel, T. Fleury, H. Choi, L. Pyle, L. Yu, M. J. Rewers, M. Nakayama, A. W. Michels, Tracking DNA-based antigen-specific T cell receptors during progression to type 1 diabetes. *Sci. Adv.* **9**, eadj6975 (2023).

68. J. Gutierrez-Achury, J. Romanos, S. F. Bakker, V. Kumar, E. C. de Haas, G. Trynka, I. Ricaño-Ponce, A. Steck, Type 1 Diabetes Genetics Consortium, W.-M. Chen, S. Onengut-Gumuscu, S. Simsek, Diabeter, M. Rewers, C. J. Mulder, E. Liu, S. S. Rich, C. Wijmenga, Contrasting the Genetic background of type 1 diabetes and celiac disease autoimmunity. *Diabetes Care* **38**, S37–S44 (2015).

69. L.-K. Chen, Y.-C. Chou, S.-T. Tsai, S.-J. Hwang, S.-D. Lee, Hepatitis C virus infection-related type 1 diabetes mellitus. *Diabet. Med.* **22**, 340–343 (2005).

70. H. Masuda, T. Atsumi, A. Fujisaku, C. Shimizu, N. Yoshioka, T. Koike, Acute onset of type 1 diabetes accompanied by acute hepatitis C: The potential role of proinflammatory cytokine in the pathogenesis of autoimmune diabetes. *Diabetes Res. Clin. Pract.* **75**, 357–361 (2007).

71. M. Taguchi, N. Ihana-Sugiyama, D. Shiojiri, K. Izumi, M. Kobayashi, N. Kodani, R. Bouchi, M. Ohsugi, A. Tanabe, K. Ueki, H. Kajio, New-onset type 1 diabetes and Graves' disease after antiretroviral therapy in a patient with human immunodeficiency virus infection. *J. Diabetes Investig.* **14**, 489–493 (2023).

72. Min-ChunYeh, H.-C. Chuang, S.-F. Weng, C.-H. Hsu, C.-L. Huang, Y.-P. Lin, Y.-Y. Lin, Y.-S. Hsieh, Newly diagnosed type 1 diabetes mellitus in a human immunodeficiency virus-infected

patient with antiretroviral therapy-induced immune reconstitution inflammatory syndrome: A case report. *BMC Infect. Dis.* **23**, 619 (2023).

73. E. K. Kendall, V. R. Olaker, D. C. Kaelber, R. Xu, P. B. Davis, Association of SARS-CoV-2 infection with new-onset type 1 diabetes among pediatric patients from 2020 to 2021. *JAMA Netw. Open* **5**, e2233014 (2022).

74. I. Ekman, T. Vuorinen, M. Knip, R. Veijola, J. Toppari, H. Hyöty, T. Kinnunen, J. Ilonen, J. Lempainen, Early childhood CMV infection may decelerate the progression to clinical type 1 diabetes. *Pediatr. Diabetes* **20**, 73–77 (2019).

75. C. Y. Pak, H. M. Eun, R. G. McArthur, J. W. Yoon, Association of cytomegalovirus infection with autoimmune type 1 diabetes. *Lancet* **2**, 1–4 (1988).

76. D. H. May, S. Woodhouse, B. Howie, H. S. Robins, A catalog of the public T-cell response to cytomegalovirus. bioRxiv 593237 [Preprint] (2024). https://doi.org/10.1101/2024.05.08.593237.

77. M. Messemaker, B. P. Y. Kwee, Ž. Moravec, D. Álvarez-Salmoral, J. Urbanus, S. de Paauw, J. Geerligs, R. Voogd, B. Morris, A. Guislain, M. Mußmann, Y. Winkler, M. Steinmetz, M. Iras, E. Marcus, J. Teuwen, A. Perrakis, R. L. Beijersbergen, W. Scheper, T. N. Schumacher, A functionally validated TCR-pMHC database for TCR specificity model development. bioRxiv 651095 [Preprint] (2025). https://doi.org/10.1101/2025.04.28.651095.

78. J. Ilonen, M. Sjöroos, M. Knip, R. Veijola, O. Simell, H. K. Akerblom, P. Paschou, E. Bozas, B. Havarani, A. Malamitsi-Puchner, J. Thymelli, A. Vazeou, C. S. Bartsocas, Estimation of genetic risk for type 1 diabetes. *Am. J. Med. Genet.* **115**, 30–36 (2002).

79. X. Hu, A. J. Deutsch, T. L. Lenz, S. Onengut-Gumuscu, B. Han, W.-M. Chen, J. M. M. Howson, J. A. Todd, P. I. W. de Bakker, S. S. Rich, S. Raychaudhuri, Additive and interaction effects at three amino acid positions in HLA-DQ and HLA-DR molecules drive type 1 diabetes risk. *Nat. Genet.* **47**, 898–905 (2015).

80. F. Pociot, Å. Lernmark, Genetic risk factors for type 1 diabetes. *Lancet* **387**, 2331–2339 (2016).

81. C. J. Smith, S. Strausz, FinnGen, J. P. Spence, H. M. Ollila, J. K. Pritchard, Haplotype analysis reveals pleiotropic disease associations in the HLA region. medRxiv 24311183 [Preprint] (2024). https://doi.org/10.1101/2024.07.29.24311183.

82. A. R. García, A. Paterou, M. Lee, H. Sławiński, R. Ferreira, L. G. Landry, D. Trzupek, L. Teyton, A. Szypowska, L. S. Wicker, M. Nakayama, J. A. Todd, M. Ł. Pękalski, HLA class II mediates type 1 diabetes risk by anti-insulin repertoire selection. bioRxiv 458974 [Preprint] (2021). https://doi.org/10.1101/2021.09.06.458974.

83. A. R. García, A. Paterou, R. D. Powell Doherty, L. G. Landry, M. Lee, A. M. Anderson, C. L. Scudder, H. Slawinski, R. C. Ferreira, D. Trzupek, A. Szypowska, L. Teyton, N. Ternette, M. Nakayama, L. S. Wicker, J. A. Todd, M. L. Pekalski, Autoimmune interactions between the HLA-DQβ1_{57} polymorphism, T cell receptors, and microbial mimics of insulin in type 1 diabetes. medRxiv 22274678 [Preprint] (2022) https://doi.org/10.1101/2022.05.11.22274678.

84. K. M. Ashby, K. A. Hogquist, A guide to thymic selection of T cells. *Nat. Rev. Immunol.* **24**, 103–117 (2024).

85. H. ElAbd, A. Mahdy, E. M. Wacker, M. Gretsova, D. Ellinghaus, A. Franke, Decoding the restriction of T cell receptors to human leukocyte antigen alleles using statistical learning. bioRxiv 636910 [Preprint] (2025). https://doi.org/10.1101/2025.02.06.636910.

86. A. Pugliese, Autoreactive T cells in type 1 diabetes. *J. Clin. Invest.* **127**, 2881–2891 (2017).

87. S. Liu, P. Bradley, W. Sun, Neural network models for sequence-based TCR and HLA association prediction. *PLOS Comput. Biol.* **19**, e1011664 (2023).

88. M. Nakayama, N. Abiru, H. Moriyama, N. Babaya, E. Liu, D. Miao, L. Yu, D. R. Wegmann, J. C. Hutton, J. F. Elliott, G. S. Eisenbarth, Prime role for an insulin epitope in the development of type 1 diabetes in NOD mice. *Nature* **435**, 220–223 (2005).

89. C. L. Williams, R. Fareed, G. L. M. Mortimer, R. J. Aitken, I. V. Wilson, G. George, K. M. Gillespie, A. J. K. Williams, BOX Study Group, A. E. Long, The longitudinal loss of islet autoantibody responses from diagnosis of type 1 diabetes occurs progressively over follow-up

and is determined by low autoantibody titres, early-onset, and genetic variants. *Clin. Exp. Immunol.* **210**, 151–162 (2022).

90. A. J. Brown, J. White, L. Shaw, J. Gross, A. Slabodkin, E. Kushner, V. Greiff, J. Matsuda, L. Gapin, J. Scott-Browne, J. Kappler, P. Marrack, MHC heterozygosity limits T cell receptor variability in CD4 T cells. *Sci. Immunol.* **9**, eado5295 (2024).

91. Q. Marcou, T. Mora, A. M. Walczak, High-throughput immune repertoire analysis with IGoR. *Nat. Commun.* **9**, 561 (2018).

92. G. Isacchini, A. M. Walczak, *T. Mora*, A. Nourmohammad, Deep generative selection models of T and B cell receptor repertoires with soNNia. *Proc. Natl. Acad. Sci. U.S.A.* **118**, e2023141118 (2021).

93. C. Krishna, D. Chowell, M. Gönen, Y. Elhanati, T. A. Chan, Genetic and environmental determinants of human TCR repertoire diversity. *Immun. Ageing* **17**, 26 (2020).

94. M. Liu, J. Goo, Y. Liu, W. Sun, M. C. Wu, L. Hsu, Q. He, TCR-L: An analysis tool for evaluating the association between the T-cell receptor repertoire and clinical phenotypes. *BMC Bioinformatics* **23**, 152 (2022).

95. J. A. Noble, H. A. Erlich, Genetics of type 1 diabetes. *Cold Spring Harb. Perspect. Med.* **2**, a007732 (2012).

96. M. Pavlović, L. Scheffer, K. Motwani, C. Kanduri, R. Kompova, N. Vazov, K. Waagan, F. L. M. Bernal, A. A. Costa, B. Corrie, R. Akbar, G. S. Al Hajj, G. Balaban, T. M. Brusko, M. Chernigovskaya, S. Christley, L. G. Cowell, R. Frank, I. Grytten, S. Gundersen, I. H. Haff, E. Hovig, P.-H. Hsieh, G. Klambauer, M. L. Kuijjer, C. Lund-Andersen, A. Martini, T. Minotto, J. Pensar, K. Rand, E. Riccardi, P. A. Robert, A. Rocha, A. Slabodkin, I. Snapkov, L. M. Sollid, D. Titov, C. R. Weber, M. Widrich, G. Yaari, V. Greiff, G. K. Sandve, The immuneML ecosystem for machine learning analysis of adaptive immune receptor repertoires. *Nat. Mach. Intell.* **3**, 936–944 (2021).

97. N. Conrad, S. Misra, J. Y. Verbakel, G. Verbeke, G. Molenberghs, P. N. Taylor, J. Mason, N. Sattar, J. J. V. McMurray, I. B. McInnes, K. Khunti, G. Cambridge, Incidence, prevalence, and co-occurrence of autoimmune disorders over time and by age, sex, and socioeconomic status: A population-based cohort study of 22 million individuals in the UK. *Lancet* **401**, 1878–1890 (2023).

98. M. E. Zaslavsky, E. Craig, J. K. Michuda, N. Sehgal, N. Ram-Mohan, J.-Y. Lee, K. D. Nguyen, R. A. Hoh, T. D. Pham, K. Röltgen, B. Lam, E. S. Parsons, S. R. Macwana, W. DeJager, E. M. Drapeau, K. M. Roskin, C. Cunningham-Rundles, M. Anthony Moody, B. F. Haynes, J. D. Goldman, J. R. Heath, K. C. Nadeau, B. A. Pinsky, C. A. Blish, S. E. Hensley, K. Jensen, E. Meyer, I. Balboni, P. J. Utz, J. T. Merrill, J. M. Guthridge, J. A. James, S. Yang, R. Tibshirani, A. Kundaje, S. D. Boyd, Disease diagnostics using machine learning of immune receptors. bioRxiv 489314 [Preprint] (2024). https://doi.org/10.1101/2022.04.26.489314.

99. M. Chen, Y. Zhao, Z. Wang, B. He, J. Yao, A noisy-label-learning formulation for immune repertoire classification and disease-associated immune receptor sequence identification. arXiv:2307.15934 [cs.LG] (2023).

100. Y. Katayama, T. J. Kobayashi, Comparative study of repertoire classification methods reveals data efficiency of k-mer feature extraction. *Front. Immunol.* **13**, 797640 (2022).

101. M. E. Zaslavsky, E. Craig, J. K. Michuda, N. Sehgal, N. Ram-Mohan, J.-Y. Lee, K. D. Nguyen, R. A. Hoh, T. D. Pham, K. Röltgen, B. Lam, E. S. Parsons, S. R. Macwana, W. DeJager, E. M. Drapeau, K. M. Roskin, C. Cunningham-Rundles, M. A. Moody, B. F. Haynes, J. D. Goldman, J. R. Heath, R. S. Chinthrajah, K. C. Nadeau, B. A. Pinsky, C. A. Blish, S. E. Hensley, K. Jensen, E. Meyer, I. Balboni, P. J. Utz, J. T. Merrill, J. M. Guthridge, J. A. James, S. Yang, R. Tibshirani, A. Kundaje, S. D. Boyd, Disease diagnostics using machine learning of B cell and T cell receptor sequences. *Science* **387**, eadp2407 (2025).

102. M. Sundararajan, A. Taly, Q. Yan, "Axiomatic Attribution for Deep Networks," in *Proceedings of the 34th International Conference on Machine Learning*, D. Precup, Y. W. Teh, Eds. (PMLR, 2017), vol. 70, pp. 3319–3328.

103. J. Ostmeyer, E. Lucas, S. Christley, J. Lea, N. Monson, J. Tiro, L. G. Cowell, Biophysicochemical motifs in T cell receptor sequences as a potential biomarker for high-grade serous ovarian carcinoma. *PLOS ONE* **15**, e0229569 (2020).

104. L. Scheffer, E. E. Reber, B. B. Mehta, M. Pavlović, M. Chernigovskaya, E. Richardson, R. Akbar, F. Lund-Johansen, V. Greiff, I. H. Haff, G. K. Sandve, Predictability of antigen binding based on short motifs in the antibody CDRH3. *Brief. Bioinform.* **25**, bbae537 (2024).

105. M. J. Redondo, S. Onengut-Gumuscu, K. J. Gaulton, "Genetics of type 1 diabetes," in *Diabetes in America* (National Institute of Diabetes and Digestive and Kidney Diseases, 2023).

106. P. Majumder, J. T. Lee, A. R. Rahmberg, G. Kumar, T. Mi, C. D. Scharer, J. M. Boss, A super enhancer controls expression and chromatin architecture within the MHC class II locus. *J. Exp. Med.* **217**, e20190668 (2020).

107. U. Võsa, A. Claringbould, H.-J. Westra, M. J. Bonder, P. Deelen, B. Zeng, H. Kirsten, A. Saha, R. Kreuzhuber, S. Yazar, H. Brugge, R. Oelen, D. H. de Vries, M. G. P. van der Wijst, S. Kasela, N. Pervjakova, I. Alves, M.-J. Favé, M. Agbessi, M. W. Christiansen, R. Jansen, I. Seppälä, L. Tong, A. Teumer, K. Schramm, G. Hemani, J. Verlouw, H. Yaghootkar, R. Sönmez Flitman, A. Brown, V. Kukushkina, A. Kalnapenkis, S. Rüeger, E. Porcu, J. Kronberg, J. Kettunen, B. Lee, F. Zhang, T. Qi, J. A. Hernandez, W. Arindrarto, F. Beutner, BIOS Consortium, i2QTL Consortium, J. Dmitrieva, M. Elansary, B. P. Fairfax, M. Georges, B. T. Heijmans, A. W. Hewitt, M. Kähönen, Y. Kim, J. C. Knight, P. Kovacs, K. Krohn, S. Li, M. Loeffler, U. M. Marigorta, H. Mei, Y. Momozawa, M. Müller-Nurasyid, M. Nauck, M. G. Nivard, B. W. J. H. Penninx, J. K. Pritchard, O. T. Raitakari, O. Rotzschke, E. P. Slagboom, C. D. A. Stehouwer, M. Stumvoll, P. Sullivan, P. A. C. 't Hoen, J. Thiery, A. Tönjes, J. van Dongen, M. van Iterson, J. H. Veldink, U. Völker, R. Warmerdam, C. Wijmenga, M. Swertz, A. Andiappan, G. W. Montgomery, S. Ripatti, M. Perola, Z. Kutalik, E. Dermitzakis, S. Bergmann, T. Frayling, J. van Meurs, H. Prokisch, H. Ahsan, B. L. Pierce, T. Lehtimäki, D. I. Boomsma, B. M. Psaty, S. A. Gharib, P. Awadalla, L. Milani, W. H. Ouwehand, K. Downes, O. Stegle, A. Battle, P. M. Visscher, J. Yang, M. Scholz, J. Powell, G. Gibson, T. Esko, L. Franke, Large-scale

cis- and trans-eQTL analyses identify thousands of genetic loci and polygenic scores that regulate blood gene expression. *Nat. Genet.* **53**, 1300–1310 (2021).

108. M. J. Machiela, S. J. Chanock, LDlink: A web-based application for exploring population-specific haplotype structure and linking correlated alleles of possible functional variants. *Bioinformatics* **31**, 3555–3557 (2015).

109. R. A. Insel, J. L. Dunne, M. A. Atkinson, J. L. Chiang, D. Dabelea, P. A. Gottlieb, C. J. Greenbaum, K. C. Herold, J. P. Krischer, Å. Lernmark, R. E. Ratner, M. J. Rewers, D. A. Schatz, J. S. Skyler, J. M. Sosenko, A.-G. Ziegler, Staging presymptomatic type 1 diabetes: A scientific statement of JDRF, the Endocrine Society, and the American Diabetes Association. *Diabetes Care* **38**, 1964–1974 (2015).

110. P. Leete, R. Mallone, S. J. Richardson, J. M. Sosenko, M. J. Redondo, C. Evans-Molina, The effect of age on the progression and severity of type 1 diabetes: Potential effects on disease mechanisms. *Curr. Diab. Rep.* **18**, 115 (2018).

111. P. Leete, A. Willcox, L. Krogvold, K. Dahl-Jørgensen, A. K. Foulis, S. J. Richardson, N. G. Morgan, Differential insulitic profiles determine the extent of β-cell destruction and the age at onset of type 1 diabetes. *Diabetes* **65**, 1362–1369 (2016).

112. A. L. J. Carr, J. R. J. Inshaw, C. S. Flaxman, P. Leete, R. C. Wyatt, L. A. Russell, M. Palmer, D. Prasolov, T. Worthington, B. Hull, L. S. Wicker, D. B. Dunger, R. A. Oram, N. G. Morgan, J. A. Todd, S. J. Richardson, R. E. J. Besser, Circulating C-peptide levels in living children and young people and pancreatic β-cell loss in pancreas donors across type 1 diabetes disease duration. *Diabetes* **71**, 1591–1596 (2022).

113. P. Achenbach, E. Bonifacio, K. Koczwara, A.-G. Ziegler, Natural history of type 1 diabetes. *Diabetes* **54**, S25–31 (2005).

114. M. A. Atkinson, G. S. Eisenbarth, Type 1 diabetes: New perspectives on disease pathogenesis and treatment. *Lancet* **358**, 221–229 (2001).

115. A. Skowera, K. Ladell, J. E. McLaren, G. Dolton, K. K. Matthews, E. Gostick, D. Kronenberg-Versteeg, M. Eichmann, R. R. Knight, S. Heck, J. Powrie, P. J. Bingley, C. M. Dayan, J. J. Miles, A. K. Sewell, D. A. Price, M. Peakman, β-Cell-specific CD8 T cell phenotype in type 1 diabetes reflects chronic autoantigen exposure. *Diabetes* **64**, 916–925 (2015).

116. L. Krogvold, B. Edwin, T. Buanes, G. Frisk, O. Skog, M. Anagandula, O. Korsgren, D. Undlien, M. C. Eike, S. J. Richardson, P. Leete, N. G. Morgan, S. Oikarinen, M. Oikarinen, J. E. Laiho, H. Hyöty, J. Ludvigsson, K. F. Hanssen, K. Dahl-Jørgensen, Detection of a low-grade enteroviral infection in the islets of langerhans of living patients newly diagnosed with type 1 diabetes. *Diabetes* **64**, 1682–1687 (2015).

117. T. B. Strom, Can childhood viral infection protect from type 1 diabetes? *J. Clin. Invest.* **119**, 1458–1461 (2009).

118. K. A. Lagattuta, J. B. Kang, A. Nathan, K. E. Pauken, A. H. Jonsson, D. A. Rao, A. H. Sharpe, K. Ishigaki, S. Raychaudhuri, Repertoire analyses reveal T cell antigen receptor sequence features that influence T cell fate. *Nat. Immunol.* **23**, 446–457 (2022).

119. J. Textor, F. Buytenhuijs, D. Rogers, È. M. Gauthier, *S. Sultan*, I. M. N. Wortel, K. Kalies, A. Fähnrich, R. Pagel, H. J. Melichar, J. Westermann, J. N. Mandl, Machine learning analysis of the T cell receptor repertoire identifies sequence features of self-reactivity. *Cell Syst.* **14**, 1059–1073.e5 (2023).

120. M. van Lummel, P. A. van Veelen, A. Zaldumbide, A. de Ru, G. M. C. Janssen, A. K. Moustakas, G. K. Papadopoulos, J. W. Drijfhout, B. O. Roep, F. Koning, Type 1 diabetes-associated HLA-DQ8 transdimer accommodates a unique peptide repertoire. *J. Biol. Chem.* **287**, 9514–9524 (2012).

121. I.-T. Chow, T. J. Gates, G. K. Papadopoulos, A. K. Moustakas, E. M. Kolawole, R. J. Notturno, J. W. McGinty, N. Torres-Chinn, E. A. James, C. Greenbaum, G. T. Nepom, B. D. Evavold, W. W. Kwok, Discriminative T cell recognition of cross-reactive islet-antigens is associated with HLA-DQ8 transdimer-mediated autoimmune diabetes. *Sci. Adv.* **5**, eaaw9336 (2019).

122. Z. Zhou, E. Reyes-Vargas, H. Escobar, B. Rudd, A. L. Rockwood, J. C. Delgado, X. He, P. E. Jensen, Type 1 diabetes associated HLA-DQ2 and DQ8 molecules are relatively resistant to HLA-DM mediated release of invariant chain-derived CLIP peptides. *Eur. J. Immunol.* **46**, 834–845 (2016).

123. D. A. Weber, B. D. Evavold, P. E. Jensen, Enhanced dissociation of HLA-DR-bound peptides in the presence of HLA-DM. *Science* **274**, 618–620 (1996).

124. L. K. Denzin, Inhibition of HLA-DM mediated MHC class II peptide loading by HLA-DO promotes self tolerance. *Front. Immunol.* **4**, 465 (2013).

125. R. G. Naik, C. Beckers, R. Wentwoord, A. Frenken, G. Duinkerken, B. Brooks-Worrell, N. C. Schloot, J. P. Palmer, B. O. Roep, Precursor frequencies of T-cells reactive to insulin in recent onset type 1 diabetes mellitus. *J. Autoimmun.* **23**, 55–61 (2004).

126. M. T. Tran, J. J. Lim, T. J. Loh, S. I. Mannering, J. Rossjohn, H. H. Reid, A structural basis of T cell cross-reactivity to native and spliced self-antigens presented by HLA-DQ8. *J. Biol. Chem.* **300**, 107612 (2024).

127. M. R. Shapiro, X. Dong, D. J. Perry, J. M. McNichols, P. Thirawatananond, A. L. Posgai, L. D. Peters, K. Motwani, R. S. Musca, A. Muir, P. Concannon, L. M. Jacobsen, C. E. Mathews, C. H. Wasserfall, M. J. Haller, D. A. Schatz, M. A. Atkinson, M. A. Brusko, R. Bacher, T. M. Brusko, Human immune phenotyping reveals accelerated aging in type 1 diabetes. *JCI Insight* **8**, e170767 (2023).

128. C. C. Robertson, J. R. J. Inshaw, S. Onengut-Gumuscu, W.-M. Chen, D. F. S. Cruz, H. Yang, A. J. Cutler, D. J. M. Crouch, E. Farber, S. L. Bridges Jr, J. C. Edberg, R. P. Kimberly, J. H. Buckner, P. Deloukas, J. Divers, D. Dabelea, J. M. Lawrence, S. Marcovina, A. S. Shah, C. J. Greenbaum, M. A. Atkinson, P. K. Gregersen, J. R. Oksenberg, F. Pociot, M. J. Rewers, A. K. Steck, D. B. Dunger, Type 1 Diabetes Genetics Consortium, L. S. Wicker, P. Concannon, J. A. Todd, S. S. Rich, Fine-mapping, trans-ancestral and genomic analyses identify causal variants, cells, genes and drug targets for type 1 diabetes. *Nat. Genet.* **53**, 962–971 (2021).

129. V. Pathiraja, J. P. Kuehlich, P. D. Campbell, B. Krishnamurthy, T. Loudovaris, P. T. H. Coates, T. C. Brodnicki, P. J. O'Connell, K. Kedzierska, C. Rodda, P. Bergman, E. Hill, A. W. Purcell, N. L. Dudek, H. E. Thomas, T. W. H. Kay, S. I. Mannering, Proinsulin-specific, HLA-DQ8, and HLA-DQ8-transdimer-restricted $CD4^+$ T cells infiltrate islets in type 1 diabetes. *Diabetes* **64**, 172–182 (2015).

130. B. D. Stadinski, S. B. Cleveland, M. A. Brehm, D. L. Greiner, P. G. Huseby, E. S. Huseby, I-A^{g7} β56/57 polymorphisms regulate non-cognate negative selection to $CD4^+$ T cell orchestrators of type 1 diabetes. *Nat. Immunol.* **24**, 652–663 (2023).

131. R. Zander, M. Y. Kasmani, Y. Chen, P. Topchyan, J. Shen, S. Zheng, R. Burns, J. Ingram, *C. Cui*, N. Joshi, J. Craft, A. Zajac, W. Cui, Tfh-cell-derived interleukin 21 sustains effector $CD8^+$ T cell responses during chronic viral infection. *Immunity* **55**, 475–493.e5 (2022).

132. E. Bettelli, D. J. Campbell, Circulating T_{FH} cells as a marker for early therapeutic intervention in T1D. *Nat. Immunol.* **21**, 1141–1142 (2020).

133. R. C. Ferreira, H. Z. Simons, W. S. Thompson, A. J. Cutler, X. C. Dopico, D. J. Smyth, M. Mashar, H. Schuilenburg, N. M. Walker, D. B. Dunger, C. Wallace, J. A. Todd, L. S. Wicker, M. L. Pekalski, IL-21 production by $CD4^+$ effector T cells and frequency of circulating follicular helper T cells are increased in type 1 diabetes patients. *Diabetologia* **58**, 781–790 (2015).

134. A.-K. Heninger, A. Eugster, D. Kuehn, F. Buettner, M. Kuhn, A. Lindner, S. Dietz, S. Jergens, C. Wilhelm, A. Beyerlein, A.-G. Ziegler, E. Bonifacio, A divergent population of autoantigen-responsive $CD4^+$ T cells in infants prior to β cell autoimmunity. *Sci. Transl. Med.* **9**, eaaf8848 (2017).

135. A. M. Mitchell, A. W. Michels, T cell receptor sequencing in autoimmunity. *J. Life Sci.* **2**, 38–58 (2020).

136. D. H. May, S. Woodhouse, H. J. Zahid, R. Elyanow, K. Doroschak, M. T. Noakes, R. Taniguchi, Z. Yang, J. R. Grino, R. Byron, J. Oaks, A. Sherwood, J. Greissl, H. Chen-Harris, B. Howie, H. S. Robins, Identifying immune signatures of common exposures through co-

occurrence of T-cell receptors in tens of thousands of donors. bioRxiv 583354 [Preprint] (2024). https://doi.org/10.1101/2024.03.26.583354.

137. A. R. García, A. Paterou, M. Lee, H. Sławiński, L. S. Wicker, J. A. Todd, M. Ł. Pękalski, Peripheral tolerance to insulin is encoded by mimicry in the microbiome. bioRxiv 881433 [Preprint] (2019). https://doi.org/10.1101/2019.12.18.881433.

138. A.-G. Ziegler, E. Bonifacio, BABYDIAB-BABYDIET Study Group, Age-related islet autoantibody incidence in offspring of patients with type 1 diabetes. *Diabetologia* **55**, 1937–1943 (2012).

139. L.-Y. Lo, T.-M. Chan, K.-H. Lee, K.-S. Leung, "Challenges rising from learning motif evaluation functions using genetic programming," in *Proceedings of the 12th Annual Conference on Genetic and Evolutionary Computation* (ACM, 2010); http://dx.doi.org/10.1145/1830483.1830515.

140. V. Mhanna, G. Fourcade, P. Barennes, V. Quiniou, H. P. Pham, P.-G. Ritvo, F. Brimaud, B. Gouritin, G. Churlaud, A. Six, E. Mariotti-Ferrandiz, D. Klatzmann, Impaired activated/memory regulatory T cell clonal expansion instigates diabetes in NOD mice. *Diabetes* **70**, 976–985 (2021).

141. A. Eugster, A. Lorenc, M. Kotrulev, Y. Kamra, M. Goel, K. Steinberg-Bains, S. Sabbah, S. Dietz, E. Bonifacio, M. Peakman, I. Gomez-Tourino, Physiological and pathogenic T cell autoreactivity converge in type 1 diabetes. *Nat. Commun.* **15**, 9204 (2024).

142. M. V. Pogorelyy, A. M. Kirk, S. Adhikari, A. A. Minervina, B. Sundararaman, K. Vegesana, D. C. Brice, Z. B. Scott, P. G. Thomas, TIRTL-seq: Deep, quantitative, and affordable paired TCR repertoire sequencing, *Immunology* **23**, 56–64 (2024).

143. R. R. T, O. N. A. Demerdash, J. C. Smith, TCR-H: Explainable machine learning prediction of T-cell receptor epitope binding on unseen datasets. *Front. Immunol.* **15**, 1426173 (2024).

144. P. M. Zdinak, N. Trivedi, S. Grebinoski, J. Torrey, E. Z. Martinez, S. Martinez, L. Hicks, R. Ranjan, V. K. K. Makani, M. M. Roland, L. Kublo, S. Arshad, M. S. Anderson, D. A. A.

Vignali, A. V. Joglekar, De novo identification of $CD4^+$ T cell epitopes. *Nat. Methods* **21**, 846–856 (2024).

145. A. V. Joglekar, G. Li, T cell antigen discovery. *Nat. Methods* **18**, 873–880 (2021).

146. K.-Y. Ma, A. A. Schonnesen, C. He, A. Y. Xia, E. Sun, E. Chen, K. R. Sebastian, Y.-W. Guo, R. Balderas, M. Kulkarni-Date, N. Jiang, High-throughput and high-dimensional single-cell analysis of antigen-specific $CD8^+$ T cells. *Nat. Immunol.* **22**, 1590–1598 (2021).

147. A. Weber, A. Pélissier, M. Rodríguez Martínez, T-cell receptor binding prediction: A machine learning revolution. *Immunoinformatics* **15**, 100040 (2024).

148. M. Nielsen, A. Eugster, M. F. Jensen, M. Goel, A. Tiffeau-Mayer, A. Pelissier, S. Valkiers, M. R. Martínez, B. Meynard-Piganeeau, V. Greiff, *T. Mora*, A. M. Walczak, G. Croce, D. L. Moreno, D. Gfeller, P. Meysman, J. Barton, Lessons learned from the IMMREP23 TCR-epitope prediction challenge. *Immunoinformatics* **16**, 100045 (2024).

149. A. Minervina, M. Pogorelyy, I. Mamedov, T-cell receptor and B-cell receptor repertoire profiling in adaptive immunity. *Transpl. Int.* **32**, 1111–1123 (2019).

150. J. Glanville, H. Huang, A. Nau, O. Hatton, L. E. Wagar, F. Rubelt, X. Ji, A. Han, S. M. Krams, C. Pettus, N. Haas, C. S. L. Arlehamn, A. Sette, S. D. Boyd, T. J. Scriba, O. M. Martinez, M. M. Davis, Identifying specificity groups in the T cell receptor repertoire. *Nature* **547**, 94–98 (2017).

151. A. S. Shomuradova, M. S. Vagida, S. A. Sheetikov, K. V. Zornikova, D. Kiryukhin, A. Titov, I. O. Peshkova, A. Khmelevskaya, D. V. Dianov, M. Malasheva, A. Shmelev, Y. Serdyuk, D. V. Bagaev, A. Pivnyuk, D. S. Shcherbinin, A. V. Maleeva, N. T. Shakirova, A. Pilunov, D. B. Malko, E. G. Khamaganova, B. Biderman, A. Ivanov, M. Shugay, G. A. Efimov, SARS-CoV-2 epitopes are recognized by a public and diverse repertoire of human T cell receptors. *Immunity* **53**, 1245–1257.e5 (2020).

152. L. M. Jacobsen, A. Posgai, H. R. Seay, M. J. Haller, T. M. Brusko, T cell receptor profiling in type 1 diabetes. *Curr. Diab. Rep.* **17**, 118 (2017).

153. S. E. Gitelman, J. A. Bluestone, Regulatory T cell therapy for type 1 diabetes: May the force be with you. *J. Autoimmun.* **71**, 78–87 (2016).

154. R. W. Beck, W. V. Tamborlane, R. M. Bergenstal, K. M. Miller, S. N. DuBose, C. A. Hall, T1D Exchange Clinic Network, The T1D Exchange clinic registry. *J. Clin. Endocrinol. Metab.* **97**, 4383–4389 (2012).

155. A. K. Davis, S. N. DuBose, M. J. Haller, K. M. Miller, L. A. DiMeglio, K. E. Bethin, R. S. Goland, E. M. Greenberg, D. R. Liljenquist, A. J. Ahmann, S. M. Marcovina, A. L. Peters, R. W. Beck, C. J. Greenbaum, T1D Exchange Clinic Network, Prevalence of detectable C-peptide according to age at diagnosis and duration of type 1 diabetes. *Diabetes Care* **38**, 476–481 (2015).

156. C. S. Carlson, R. O. Emerson, A. M. Sherwood, C. Desmarais, M.-W. Chung, J. M. Parsons, M. S. Steen, M. A. LaMadrid-Herrmannsfeldt, D. W. Williamson, R. J. Livingston, D. Wu, B. L. Wood, M. J. Rieder, H. Robins, Using synthetic templates to design an unbiased multiplex PCR assay. *Nat. Commun.* **4**, 2680 (2013).

157. H. S. Robins, P. V. Campregher, S. K. Srivastava, A. Wacher, C. J. Turtle, O. Kahsai, S. R. Riddell, E. H. Warren, C. S. Carlson, Comprehensive assessment of T-cell receptor β-chain diversity in αβ T cells. *Blood* **114**, 4099–4107 (2009).

158. A. Dilthey, S. Leslie, L. Moutsianas, J. Shen, C. Cox, M. R. Nelson, G. McVean, Multi-population classical HLA type imputation. *PLoS Comput. Biol.* **9**, e1002877 (2013).

159. C. Wasserfall, E. Montgomery, L. Yu, A. Michels, R. Gianani, A. Pugliese, C. Nierras, J. S. Kaddis, D. A. Schatz, E. Bonifacio, M. A. Atkinson, Validation of a rapid type 1 diabetes autoantibody screening assay for community-based screening of organ donors to identify subjects at increased risk for the disease. *Clin. Exp. Immunol.* **185**, 33–41 (2016).

160. I. Marzinotto, D. L. Pittman, A. J. K. Williams, A. E. Long, P. Achenbach, M. Schlosser, B. Akolkar, W. E. Winter, V. Lampasona, participating laboratories, Islet Autoantibody Standardization Program: Interlaboratory comparison of insulin autoantibody assay performance in 2018 and 2020 workshops. *Diabetologia* **66**, 897–912 (2023).

161. Y. M. Chang, A. Wieland, Z.-R. Li, S. J. Im, D. J. McGuire, H. T. Kissick, R. Antia, R. Ahmed, T cell receptor diversity and lineage relationship between virus-specific CD8 T cell subsets during chronic lymphocytic choriomeningitis virus infection. *J. Virol.* **94**, e00935-20 (2020).

162. J. Niu, Q. Jia, Q. Ni, Y. Yang, G. Chen, X. Yang, Z. Zhai, H. Yu, P. Guan, R. Lin, Z. Song, Q.-J. Li, F. Hao, H. Zhong, Y. Wan, Association of $CD8^+$ T lymphocyte repertoire spreading with the severity of DRESS syndrome. *Sci. Rep.* **5**, 9913 (2015).

163. R. Sokal, C. Michener, A statistical method for evaluating systematic relationships. *Univ. Kansas Sci. Bull.* **38**, 1409–1438 (1958).

164. A. Rényi, "On measures of entropy and information," in *Proceedings of the Fourth Berkeley Symposium on Mathematical Statistics and Probability, Volume 1: Contributions to the Theory of Statistics* (University of California Press, 1961), vol. 4, pp. 547–562.

165. M. O. Hill, Diversity and evenness: A unifying notation and its consequences. *Ecology* **54**, 427–432 (1973).

166. L. Jost, Entropy and diversity. *Oikos* **113**, 363–375 (2006).

167. H. S. Horn, Measurement of "overlap" in comparative ecological studies. *Am. Nat.* **100**, 419–424 (1966).

168. T. Rognes, L. Scheffer, V. Greiff, G. K. Sandve, CompAIRR: Ultra-fast comparison of adaptive immune receptor repertoires by exact and approximate sequence matching. *Bioinformatics* **38**, 4230–4232 (2022).

169. S. Valkiers, M. Van Houcke, K. Laukens, P. Meysman, ClusTCR: A python interface for rapid clustering of large sets of CDR3 sequences with unknown antigen specificity. *Bioinformatics* **37**, 4865–4867 (2021).

170. T. Chotisorayuth, A. Tiffeau-Mayer, Lightning-fast adaptive immune receptor similarity search by symmetric deletion lookup. arXiv:2403.09010 [q-bio.QM] (2024).

171. S. Liu, P. Bradley, W. Sun, Neural network models for sequence-based TCR and HLA association prediction. bioRxiv 542327 [Preprint] (2023). https://doi.org/10.1101/2023.05.25.542327.

172. Y. Benjamini, Y. Hochberg, Controlling the false discovery rate: A practical and powerful approach to multiple testing. *J. R. Stat. Soc.* **57**, 289–300 (1995).

173. K. Mayer-Blackwell, S. Schattgen, L. Cohen-Lavi, J. C. Crawford, A. Souquette, J. A. Gaevert, T. Hertz, P. G. Thomas, P. Bradley, A. Fiore-Gartland, TCR meta-clonotypes for biomarker discovery with tcrdist3 enabled identification of public, HLA-restricted clusters of SARS-CoV-2 TCRs. *eLife* **10**, e68605 (2021).

174. A. Hagberg, D. Schult, P. Swart, J. M. Hagberg, "Exploring network structure, dynamics, and function using NetworkX," in *Proceedings of the 7th Python in Science Conference* (SciPy, 2008), pp. 11–15.

175. M.-P. Lefranc, V. Giudicelli, C. Ginestoux, J. Jabado-Michaloud, G. Folch, F. Bellahcene, Y. Wu, E. Gemrot, X. Brochet, J. Lane, L. Regnier, F. Ehrenmann, G. Lefranc, P. Duroux, IMGT, the international ImMunoGeneTics information system. *Nucleic Acids Res.* **37**, D1006–D1012 (2009).

176. S. Sakaue, S. Gurajala, M. Curtis, Y. Luo, W. Choi, K. Ishigaki, J. B. Kang, L. Rumker, A. J. Deutsch, S. Schönherr, L. Forer, J. LeFaive, C. Fuchsberger, B. Han, T. L. Lenz, P. I. W. de Bakker, Y. Okada, A. V. Smith, S. Raychaudhuri, Tutorial: A statistical genetics guide to identifying HLA alleles driving complex disease. *Nat. Protoc.* **18**, 2625–2641 (2023).

177. H. Ramsauer, B. Schäfl, J. Lehner, P. Seidl, M. Widrich, T. Adler, L. Gruber, M. Holzleitner, M. Pavlović, G. K. Sandve, V. Greiff, D. Kreil, M. Kopp, G. Klambauer, J. Brandstetter, S. Hochreiter, Hopfield networks is all you need. arXiv:2008.02217 [cs.NE] (2020).

178. M. C. Frith, N. F. W. Saunders, B. Kobe, T. L. Bailey, Discovering sequence motifs with arbitrary insertions and deletions. *PLoS Comput. Biol.* **4**, e1000071 (2008).

179. D. H. Alexander, J. Novembre, K. Lange, Fast model-based estimation of ancestry in unrelated individuals. *Genome Res.* **19**, 1655–1664 (2009).

180. 1000 Genomes Project Consortium, A. Auton, L. D. Brooks, R. M. Durbin, E. P. Garrison, H. M. Kang, J. O. Korbel, J. L. Marchini, S. McCarthy, G. A. McVean, G. R. Abecasis, A global reference for human genetic variation. *Nature* **526**, 68–74 (2015).

181. S. Christley, A. Aguiar, G. Blanck, F. Breden, S. A. C. Bukhari, C. E. Busse, J. Jaglale, S. L. Harikrishnan, U. Laserson, B. Peters, A. Rocha, C. A. Schramm, S. Taylor, J. A. Vander Heiden, B. Zimonja, C. T. Watson, B. Corrie, L. G. Cowell, The ADC API: A web API for the programmatic query of the AIRR Data Commons. *Front. Big Data* **3**, 22 (2020).

182. B. D. Corrie, N. Marthandan, B. Zimonja, J. Jaglale, Y. Zhou, E. Barr, N. Knoetze, F. M. W. Breden, S. Christley, J. K. Scott, L. G. Cowell, F. Breden, iReceptor: A platform for querying and analyzing antibody/B-cell and T-cell receptor repertoire data across federated repositories. *Immunol. Rev.* **284**, 24–41 (2018).

183. C. T. Boughter, M. Meier-Schellersheim, Conserved biophysical compatibility among the highly variable germline-encoded regions shapes TCR-MHC interactions. *eLife* **12**, e90681 (2023).

184. T. A. Aly, A. Ide, M. M. Jahromi, J. M. Barker, M. S. Fernando, S. R. Babu, L. Yu, D. Miao, H. A. Erlich, P. R. Fain, K. J. Barriga, J. M. Norris, M. J. Rewers, G. S. Eisenbarth, Extreme genetic risk for type 1A diabetes. *Proc. Natl. Acad. Sci. U.S.A.* **103**, 14074–14079 (2006).

185. K. F. B. Gomes, A. S. Santos, C. Semzezem, M. R. Correia, L. A. Brito, M. O. Ruiz, R. T. Fukui, S. R. Matioli, M. R. Passos-Bueno, M. E. R. da Silva, The influence of population stratification on genetic markers associated with type 1 diabetes. *Sci. Rep.* **7**, 43513 (2017).
